# Identification of a New Prognostic Risk Signature of Clear Cell Renal Cell Carcinoma Based on N^6^-Methyladenosine RNA Methylation Regulators

**DOI:** 10.1155/2021/6617841

**Published:** 2021-02-12

**Authors:** Yan Zhang, Yao Yao, Xiaochen Qi, Jianyi Li, Meihong Liu, Xiangyu Che, Yingkun Xu, Guangzhen Wu

**Affiliations:** ^1^Department of Laboratory, The First Affiliated Hospital of Dalian Medical University, Dalian, China; ^2^Department of Clinical Laboratory, The First People's Hospital of Linhai, Taizhou, China; ^3^Department of Urology, The First Affiliated Hospital of Dalian Medical University, Dalian, China; ^4^Organ Transplant Center, The First Affiliated Hospital, Sun Yat-sen University, Guangzhou, China; ^5^Department of Respiratory Medicine, The First Affiliated Hospital of Dalian Medical University, Dalian, China; ^6^Department of Urology, Shandong Provincial Hospital, Cheeloo College of Medicine, Shandong University, Jinan, China

## Abstract

As the most prevalent internal eukaryotic modification, N^6^-methyladenosine (m^6^A) is installed by methyltransferases, removed by demethylases, and recognized by readers. However, there are few studies on the role of m^6^A in clear cell renal cell carcinoma (ccRCC). In this study, we researched the RNA-seq transcriptome data of ccRCC in the TCGA dataset and used bioinformatics analyses to detect the relationship between m^6^A RNA methylation regulators and ccRCC. First, we compared the expression of 18 m^6^A RNA methylation regulators in ccRCC patients and normal tissues. Then, data from ccRCC patients were divided into two clusters by consensus clustering. LASSO Cox regression analysis was used to build a risk signature to predict the prognosis of patients with ccRCC. An ROC curve, univariate Cox regression analysis, and multivariate Cox regression analysis were used to verify this risk signature's predictive ability. Then, we internally validated this signature by random sampling. Finally, we explored the role of the genes in the signature in some common pathways. Gene distribution between the two subgroups was different; cluster 2 was gender-related and had a worse prognosis. IGF2BP3, IGF2BP2, HNRNPA2B1, and METTL14 were chosen to build the risk signature. The overall survival of the high- and low-risk groups was significantly different (*p* = 7.47*e* − 12). The ROC curve also indicated that the risk signature had a decent predictive significance (AUC = 0.72). These results imply that the risk signature has a potential value for ccRCC treatment.

## 1. Introduction

As one of the most common types of kidney cancer in adults, renal cell carcinoma (RCC) accounts for nearly 3% of adult malignant tumors in the US [1]. Clear cell renal cell carcinoma (ccRCC) is the most common histological subtype of RCC [2]. The exact cause of ccRCC is uncertain, but smoking and several genetic predisposition conditions may be related to its development. ccRCC has the worst prognosis among all renal epithelial tumors. At present, surgery is considered an effective treatment, but there are still 20%–40% of patients with postoperative metastasis or recurrence [3]. Therefore, we aimed to find a way to evaluate the prognosis of ccRCC (to make specific judgments of prognoses), determine accurate biomarkers for patients, and reduce mortality.

N^6^-Methyladenosine (m^6^A) is a modified adenosine residue, methylated at position N^6^ [4]. It is involved in a series of mRNA metabolism processes, such as mRNA stability, splicing, transport, and translation, and plays an important role in the fate of mRNA. m^6^A is mainly located within the consensus sequence RRACH (R = G or A; H = A, C, or U) [5, 6]. This sequence is enriched near 3′-untranslated regions as well as in stop codon regions of protein-encoded mRNAs [7, 8]; in addition, when present in the 5′-UTR, mRNAs can be translated in a cap-independent manner [9]. m^6^A is the most common and abundant internal transcriptional modification found in RNAs in eukaryotic cells [4, 10, 11]. There have been many recent studies on m^6^A, and results indicate that m^6^A methylation contributes to the pathogenesis and progression of tumors [12, 13] [14, 15] and even those cancer responses to treatments are related to m^6^A [16–18].

**Figure 1:**
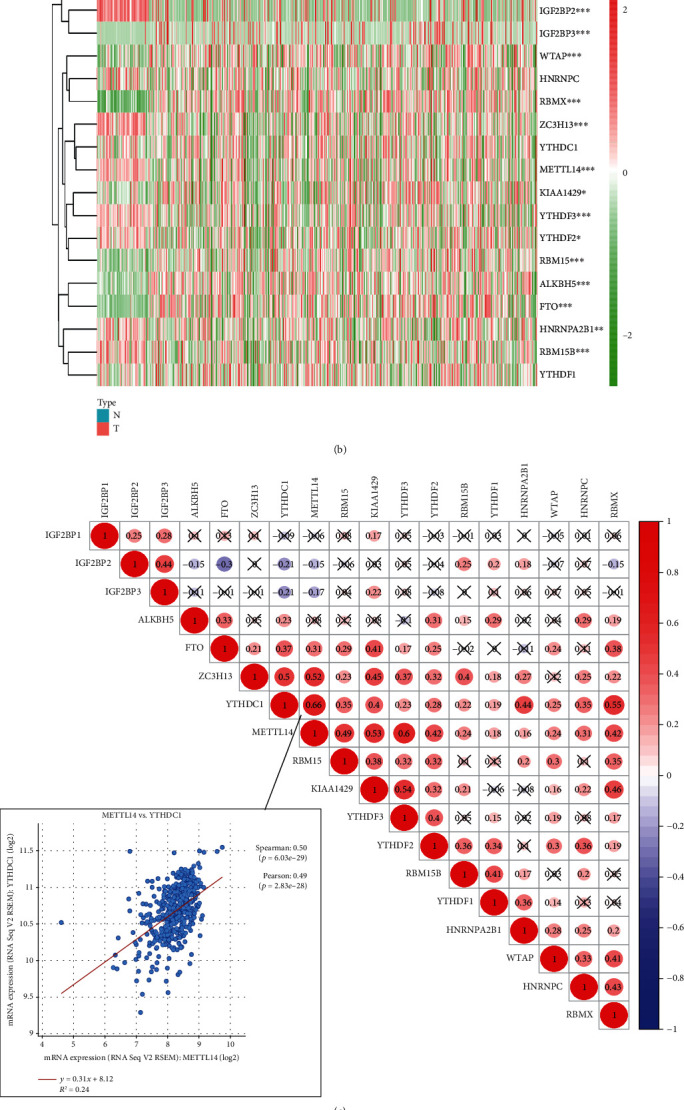
The panorama of m^6^A RNA methylation regulators in ccRCC. (a) The m^6^A RNA methylation process and the regulators involved. (b) Expression levels of 18 m^6^A RNA methylation regulators in ccRCC and normal tissues. The upper tree diagram represents grouping results for the samples, whereas the tree on the left represents cluster analysis results for regulators. Highly expressed genes are represented by a red-colored gradient: the highest the expression, the darker the red tone. In contrast, lowly expressed genes are represented by a green-colored gradient, being the genes with the lowest expression the darker ones. (c) Spearman correlation analysis of the 18 m^6^A RNA methylation regulators in ccRCC and verification of the correlation between YTHDC1 and RBM15. (d) Vioplot visualizing differentially expressed m^6^A RNA methylation regulators in ccRCC. The *x*-axis represents different genes, the *y*-axis represents gene expression, blue represents normal kidney tissue, and red represents ccRCC tissue. ^∗^*p* < 0.05, ^∗∗^*p* < 0.01, and ^∗∗∗^*p* < 0.001.

The dynamic process of m^6^A modification is orchestrated by writers (methyltransferase complexes), erasers (demethylases), and readers (Figure 1(a)). Writers, such as METTL14, WTAP, and KIAA1429, catalyze the adenylate mRNA m^6^A modification, whereas the complex composed of METTL3, METTL14, and KIAA1429 causes the m^6^A methylated group to be written into RNA [19]. Erasers, such as FTO and ALKHB5, cause the demethylation of the base [10, 20, 21]. Finally, readers play an important role in RNA metabolism; they recognize the base modified by m^6^A, bind to the methylation site, and activate the downstream physical process [22–24]; proteins from the YTH domain family, together with IGF2BP1-3 and HNRNPA2B1, belong to the group of reader proteins. Some studies have mentioned that m^6^A regulators could be used as prognostic biomarkers in ccRCC, but these studies only analyzed some m^6^A regulators and did not make a complete risk signature. In this study, we collected data from 539 patients with ccRCC from The Cancer Genome Atlas (TCGA) and used bioinformatics analysis to determine the connection between m^6^A regulators and ccRCC in an attempt to identify a risk signature to predict the prognosis of patients with ccRCC.

## 2. Materials and Methods

### 2.1. Ethics Statement

This study was approved by the Ethics Committee of the First Affiliated Hospital of Dalian Medical University and conducted in accordance with the principles expressed in the Declaration of Helsinki. All datasets were retrieved from published literature, and all written informed consent was verified.

### 2.2. Data Acquisition

We systematically searched for RNA-seq transcriptome data of ccRCC in the TCGA dataset (https://cancergenome.nih.gov/) and downloaded all the matching clinical information data. During the processing of the clinical data in TCGA, we discounted patient samples with missing clinical information. As the lymph node metastasis status of most data is unknown, this factor was later removed from the analysis.

We used the data from the cBioPortal (https://www.cbioportal.org/) to verify the correlation between METTL14 and YTHDC1. To further understand the biological functions of these regulators, we used KOBAS (http://kobas.cbi.pku.edu.cn/index.php) to analyze the data obtained from GO, KEGG, and Reactome. We also searched the data on GSCALite (http://bioinfo.life.hust.edu.cn/web/GSCALite/) to identify those pathways in which the 18 regulators used in this study are active and those drugs to which they are sensitive to, and to further detect the roles of the four chosen genes in cell signaling pathways.

### 2.3. Bioinformatics Analyses

First, we used the Perl package to merge all the data and extract the information of the 18 m^6^A RNA methylation regulators for further study.

We then used R (version 3.5) software to compare the expression levels of the regulators in 539 patients with ccRCC and 72 normal kidney tissues and construct a cluster analysis tree, followed by a vioplot to clearly visualize differential expression. We also analyzed the correlation between these 18 regulators.

The consistent clustering algorithm was used to determine the clustering number of samples under the following classification parameters: (1) the growth rate of the cumulative distribution function (CDF) value was slow; (2) no small clusters were allowed; (3) the data in a cluster needed to have good clustering, implying a high correlation within the cluster. Then, we performed principal component analysis (PCA) to verify the clustering results.

Aiming to build a proper risk signature using m^6^A RNA methylation regulators in ccRCC, we used the least absolute shrinkage and selection operator (LASSO) Cox regression algorithm to choose the appropriate risk factors. The association between regulators and survival was first identified. Then, the coefficient was determined using the minimum standard. The best penalty parameter *λ* was selected to obtain the final risk score. Then, we used the risk signature to divide the patients into two subgroups and compared the overall survival (OS) of these two subgroups. Then, the receiver operating characteristic (ROC) curve was estimated, and univariate and multivariate Cox regression analyses were performed to verify the predictive ability of the risk signature. Finally, we use the GSE22541 dataset in the GEO database for external verification and random internal verification on the ccRCC dataset in the TCGA database. All these were also executed using R software package.

### 2.4. Statistical Analyses

The expression of m^6^A RNA methylation regulators in tumor tissues and normal tissues was compared by one-way ANOVA. Kaplan-Meier analysis was used to obtain survival curves [25]. *t*-tests were used to compare the expression levels in ccRCC for different clinical characteristics. We obtained the optimal cut-off value of each risk score in the training group using R software to build the risk signature. Cox regression analysis was used to evaluate the association between the risk score, other clinical characteristics, and OS. The log-rank tests were used to perform survival analyses. In all our analyses, *p* < 0.05 was considered statistically significant.

## 3. Results

### 3.1. The Panorama of m^6^A RNA Methylation Regulators in ccRCC

First, we compared the expression of the 18 m^6^A RNA methylation regulators in 539 ccRCC cancer tissues against the expression in 72 normal kidney tissues obtained from the TCGA database. Compared with normal tissues, the expression of ALKBH5, KIAA1429, RBM15B, IGF2BP2, HNRNPA2B1, YTHDF2, METTL4, ZC3H13, YTHDF3, IGF2BP3, RBMX, FTO, WTAP, and RBM15 showed significant statistical differences (Figures 1(b) and 1(d)). Next, we further explored the interactions between the 18 m^6^A RNA methylation regulators and found that such interactions could be positive, negative, or irrelevant (Figure 1(c)). We found that the two most relevant regulators were YTHDC1 and RBM15, with a mutual reinforcement correlation. To verify this conclusion, we explored the cBioPortal data and found that these two regulators had a strong expression correlation.

### 3.2. Consensus Clustering of m^6^A RNA Methylation Regulators Identified Two Clusters of ccRCC

**Figure 2:**
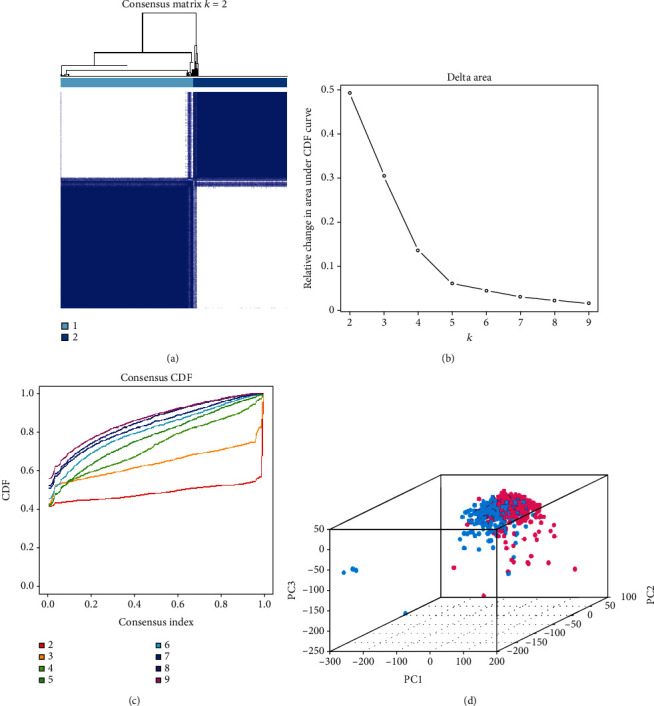
Identification of consensus clusters by m^6^A RNA methylation regulators. (a) When *k* = 2: correlation between groups. (b) Relative change in the area under the cumulative distribution function (CDF) curve for *k* values from 2 to 9. (c) Consensus clustering CDF when *k* value ranges from 2 to 9. (d) Principal component analysis of the total RNA expression profile in the TCGA dataset (cluster 1 is marked in red and cluster 2 in blue).

Next, we used consensus clustering to group the 539 ccRCC tissues. According to Figures 2(b) and 2(c), *k* = 2 or *k* = 3 values would be acceptable; however, after dividing the samples into 3 groups, some data could not be well clustered; therefore, we decided to separate our data into 2 groups. The matrix shown in Figure 2(a) represents the consensus for *k* = 2 and indicates a well-defined 2-block structure. Then, we used PCA to verify whether the grouping was appropriate (Figure 2(d)). As there were little overlapping area between clusters 1 and 2, and the data in each group gathered well, we concluded that grouping by m^6^A RNA methylation regulator expression was appropriate (*k* = 2).

### 3.3. Groups Determined by Consensus Clustering Are Closely Related to the Prognosis of ccRCC and Clinicopathological Features

**Figure 3:**
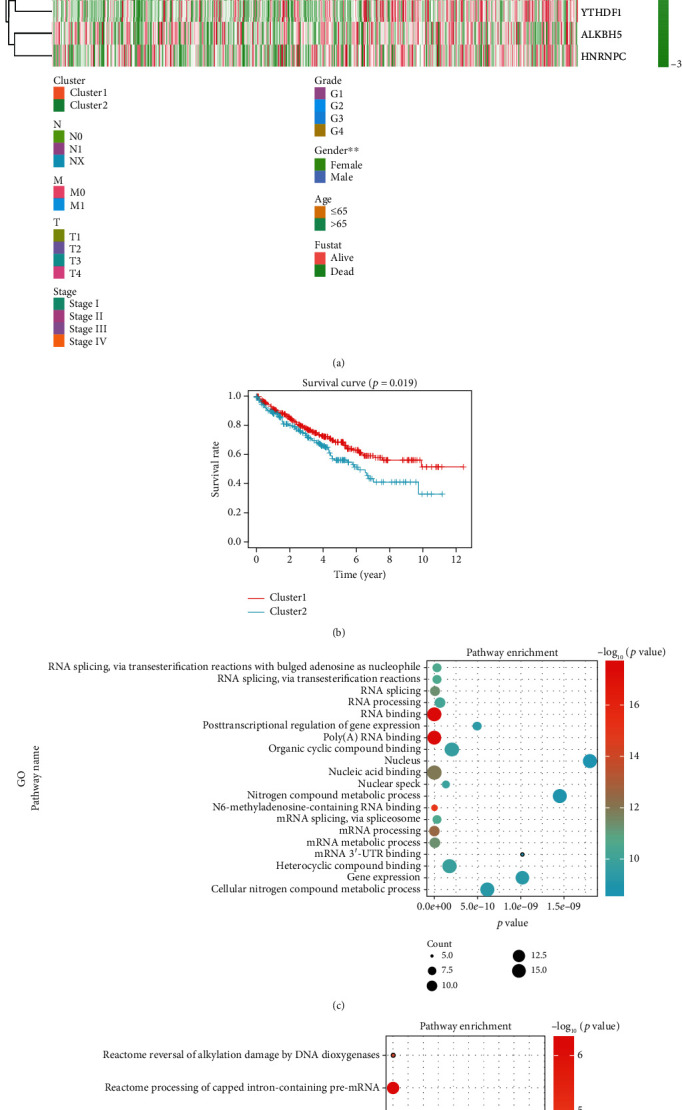
Prognosis and clinicopathological features of ccRCC. (a) The heat map and clinicopathological features of the two clusters were identified by m^6^A RNA methylation regulators. (b) Kaplan-Meier overall survival (OS) rate curve of patients with ccRCC (cluster 1 patients: red; cluster 2 patients: blue). (c, d) Results from pathway enrichment of the data using Gene Ontology (GO), KEGG, and Reactome. The size of each dot represents the pathway count. High *p* values are represented by a red-colored dot: the highest the value, the darker the red tone. In contrast, low *p* values are represented by a blue-colored dot, being the lowest values the darker ones. ^∗^*p* < 0.05, ^∗∗^*p* < 0.01, and ^∗∗∗^*p* < 0.001.

According to consensus clustering, we compared the expression levels of m^6^A RNA methylation regulators between clusters 1 and 2. Other factors such as gender, age, tumor grade, fustat, cancer stage status, and T, M, and N status were also taken into account for the comparison. We found that the expression levels of m^6^A RNA methylation regulators in clusters 1 and 2 were indeed different, and that cluster 2 was correlated with gender (Figure 3(a)). The detailed information of gene expression in clusters 1 and 2 is summarized in Supplementary Material Table S1. As shown in Figure 3(b), the OS of cluster 2 is shorter than that of cluster 1, indicating a worse clinical outcome.

### 3.4. The Role of m^6^A RNA Methylation Regulators in Various Physiological Processes or Signaling Pathways and Drug Sensitivities of m^6^A Methylation Regulators

To better understand the function of m^6^A RNA methylation regulators, we analyzed the 18 regulators using KOBAS and visualized the results using R language. Relevant data was obtained from Gene Ontology (GO), KEGG, and Reactome (Figures 3(c) and 3(d)) databases. According to the results from pathway enrichment, studied regulators are mainly involved in RNA regulation and metabolism processes, such as RNA binding, poly(A) RNA binding, and gene expression.

**Figure 4:**
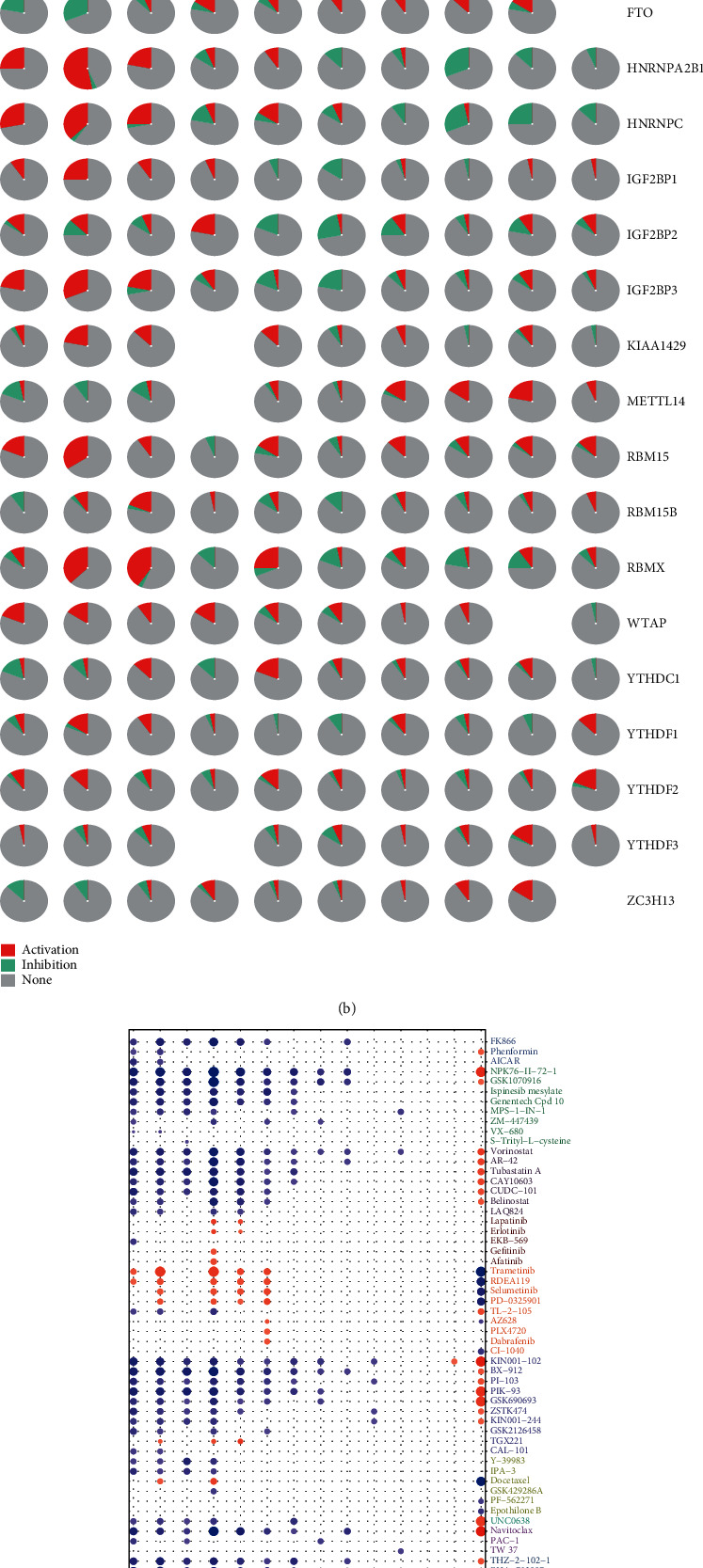
Physiological processes, signaling pathways, and drug sensitivities relevant to m^6^A methylation regulators. (a) Effect of m^6^A methylation regulators on physiological processes and signaling pathways. A: active; I: inhibited; the darker the color, the stronger the inhibition (blue) or activation (red). If a regulator activates a process or a pathway, the activation index is higher than the inhibition index. On the contrary, if the inhibition index has the highest value, then the process is inhibited. (b) Pie chart showing the results from (a) (red: activation; green: inhibition). (c) Drug sensitivities of m^6^A methylation regulators (ordinate axis: various drugs; abscissa axis: regulators).

Then, we analyzed the data on GSCALite and found that m^6^A RNA methylation regulators play important roles in many cell signaling pathways and physiological activities. HNRNPA2B1, for example, can activate apoptosis and DNA damage response, and it is also engaged in the cell cycle (Figures 4(a) and 4(b)). In addition, m^6^A RNA methylation regulators are sensitive targets for common chemotherapy drugs and targeted agents (Figure 4(c)).

### 3.5. A Risk Signature Built with Four Regulators to Evaluate Clinical Outcomes

**Figure 5:**
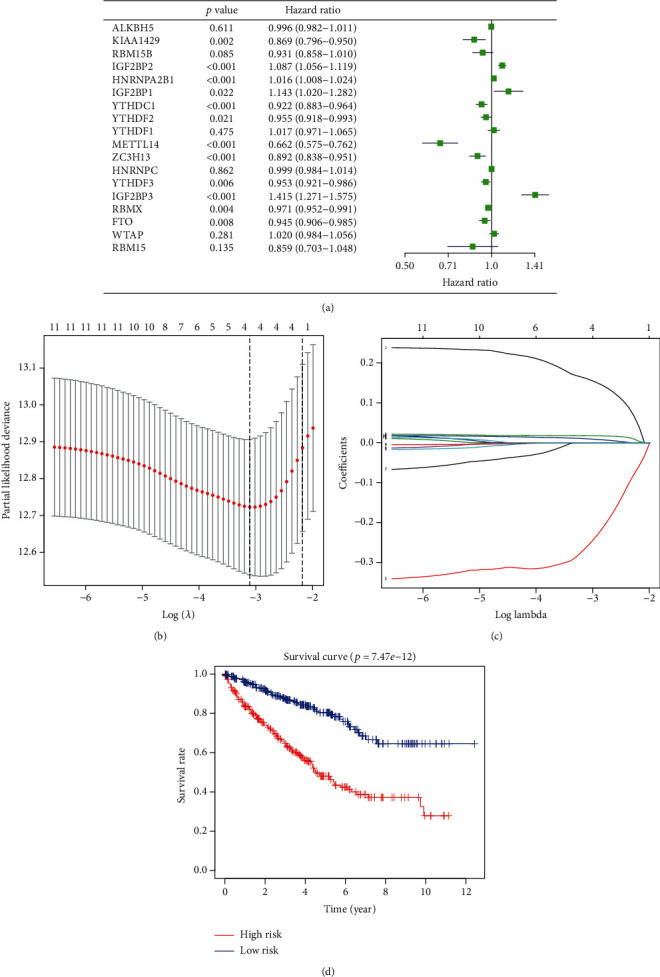
Risk signature for ccRCC. (a) Hazard ratios (HRs) and 95% confidence intervals (CIs) were calculated using univariate Cox regression. (b, c) Coefficients calculated by the least absolute shrinkage and selection operator (LASSO) multivariate Cox regression algorithm. (d) Kaplan-Meier overall survival (OS) rate curve for high-risk (red) and low-risk (blue) groups of patients.

We tried to determine whether m^6^A methylation regulators can play a prognostic role in ccRCC. Therefore, we performed a univariate Cox regression analysis on the expression levels of these regulators. As shown in Figure 5(a), patients with high expression of KIAA1429 (hazard ratio [HR] = 0.869, 95%confidence interval [CI] = 0.80 − 0.95), YTHDC1 (HR = 0.922, 95%CI = 0.88 − 0.96), YTHDF2 (HR = 0.955, 95%CI = 0.92 − 0.99), METTL14 (HR = 0.662, 95%CI = 0.58 − 0.76), ZC3H13 (HR = 0.892, 95%CI = 0.84 − 0.95), YTHDF3 (HR = 0.953, 95%CI = 0.92 − 0.99), RBMX (HR = 0.971, 95%CI = 0.95 − 0.99), and FTO (HR = 0.945, 95%CI = 0.91 − 0.99) have a better prognosis than patients with high expression of IGF2BP2 (HR = 1.087, 95%CI = 1.06 − 1.12), HNRNPA2B1 (HR = 1.016, 95%CI = 1.01 − 1.02), IGF2BP1 (HR = 1.14, 95%CI = 1.02 − 1.28), and IGF2BP3 (HR = 1.415, 95%CI = 1.27 − 1.58).

Next, we used the LASSO Cox regression algorithm to analyze the 18 regulators in the TCGA dataset and chose four of them, IGF2BP3, IGF2BP2, METTL14, and HNRNPA2B1, to build the risk signature. Selection was based on the minimum criteria and the coefficients obtained from the LASSO algorithm that were used to calculate the risk score for the TCGA dataset (Figures 5(b) and 5(c)).

To verify the prognostic ability of the four-regulator risk signature, we graded the data in the TCGA dataset and divided them into two groups according to the risk signature, the high-risk and low-risk groups, and drew the corresponding survival curves. We found that the clinical outcomes of the high-risk group were significantly worse than those of the low-risk group (Figure 5(d)).

### 3.6. The Prognostic Value of the Risk Signature Built with Four m^6^A RNA Methylation Regulators

**Figure 6:**
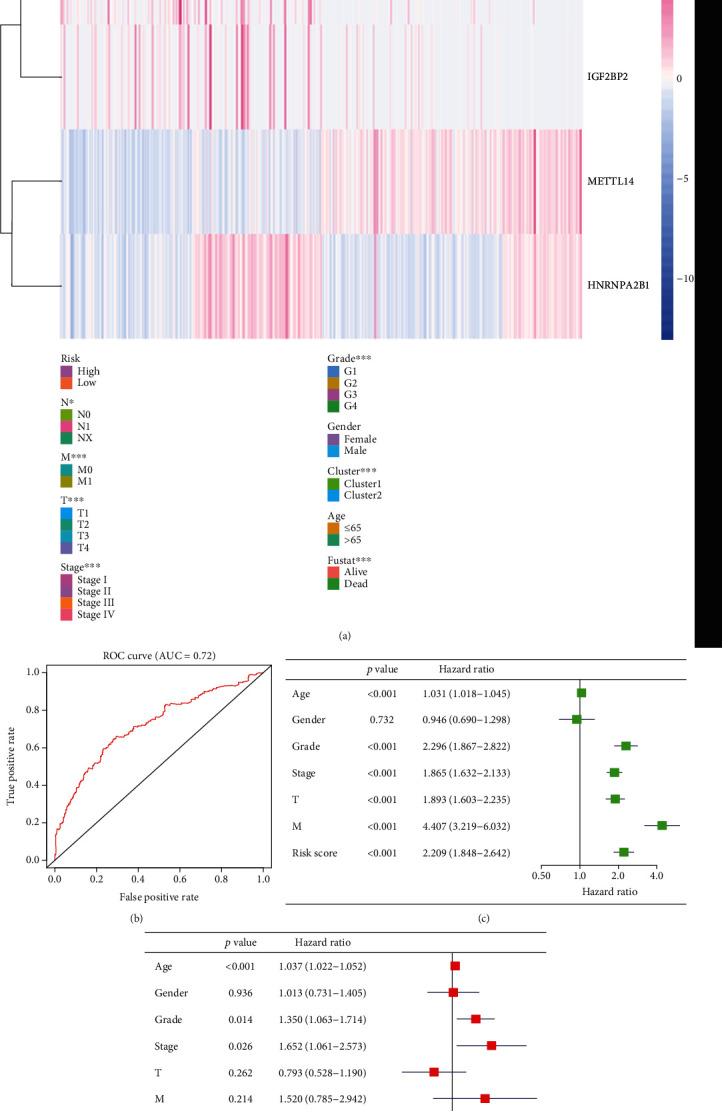
Prognosis value and accuracy of the risk signature. (a) Comparison of clinicopathological characteristics and expression of IGF2BP3, IGF2BP2, HNRNPA2B1, and METTL14 between the two groups defined by the risk signature. (b) ROC curve representing the efficiency and accuracy of the risk signature: the ROC curve for 5-year survival prediction by risk signature (date from TCGA). (c) Univariate Cox regression analysis of the association between clinicopathological factors, risk score, and overall survival of patients from TCGA datasets. (d) Multivariate Cox regression analysis of the association between clinicopathological factors, risk score, and overall survival of patients from TCGA datasets. ^∗^*p* < 0.05, ^∗∗^*p* < 0.01, and ^∗∗∗^*p* < 0.001.

We compared the expression of the four selected regulators between the low-risk and the high-risk groups. We also compared the expression considering several characteristics, such as T and M statuses, the clinical stage and grade of the tumor, the patients' age, gender, and fustat, and the cluster (1 or 2) to which the regulators belonged. After noticing that most of the data in the TCGA dataset were NX, we decided not to consider this factor in our analysis. We found that there was a high expression of IGF2BP3, IGF2BP2, and HNRNPA2B1 and a low expression of METTL14 in the high-risk group. The high-risk group also showed stronger correlations with M and T statuses, tumor stage and grade, fustat, and cluster of origin than the low-risk group (Figure 6(a)).

Receiver operating characteristic (ROC) curves were used to test the accuracy and specificity of the four-gene risk signature. The AUC = 0.72 indicated that the risk score could efficiently predict the 5-year survival of patients with ccRCC (Figure 6(b)). Then, we performed univariate and multivariate Cox regression analyses of the data from the TCGA dataset to determine whether the risk signature could be useful as an independent factor to predict the clinical outcome (Figures 6(c) and 6(d)). Results from the univariate Cox regression analysis showed that age (HR = 1.031, 95%CI = 1.02 − 1.05), grade (HR = 2.296, 95%CI = 1.87 − 2.82), stage (HR = 1.865, 95%CI = 1.63 − 2.13), T status (HR = 1.893, 95%CI = 1.60 − 2.24), M status (HR = 4.407, 95%CI = 3.22 − 6.03), and risk score (HR = 2.209, 5%CI = 1.85 − 2.64) correlated with OS. In addition, results from the multivariate Cox regression analysis indicated that age (HR = 1.037, 95%CI = 1.02 − 1.05) and risk score (HR = 1.88, 95%CI = 1.51 − 2.25) were associated with OS. Therefore, we can conclude that the risk signature can predict the prognosis of patients with ccRCC independently and in combination with other risk factors (Figures 6(c) and 6(d)).

### 3.7. Random Sampling Verification and External Verification Based on the Signature

**Figure 7:**
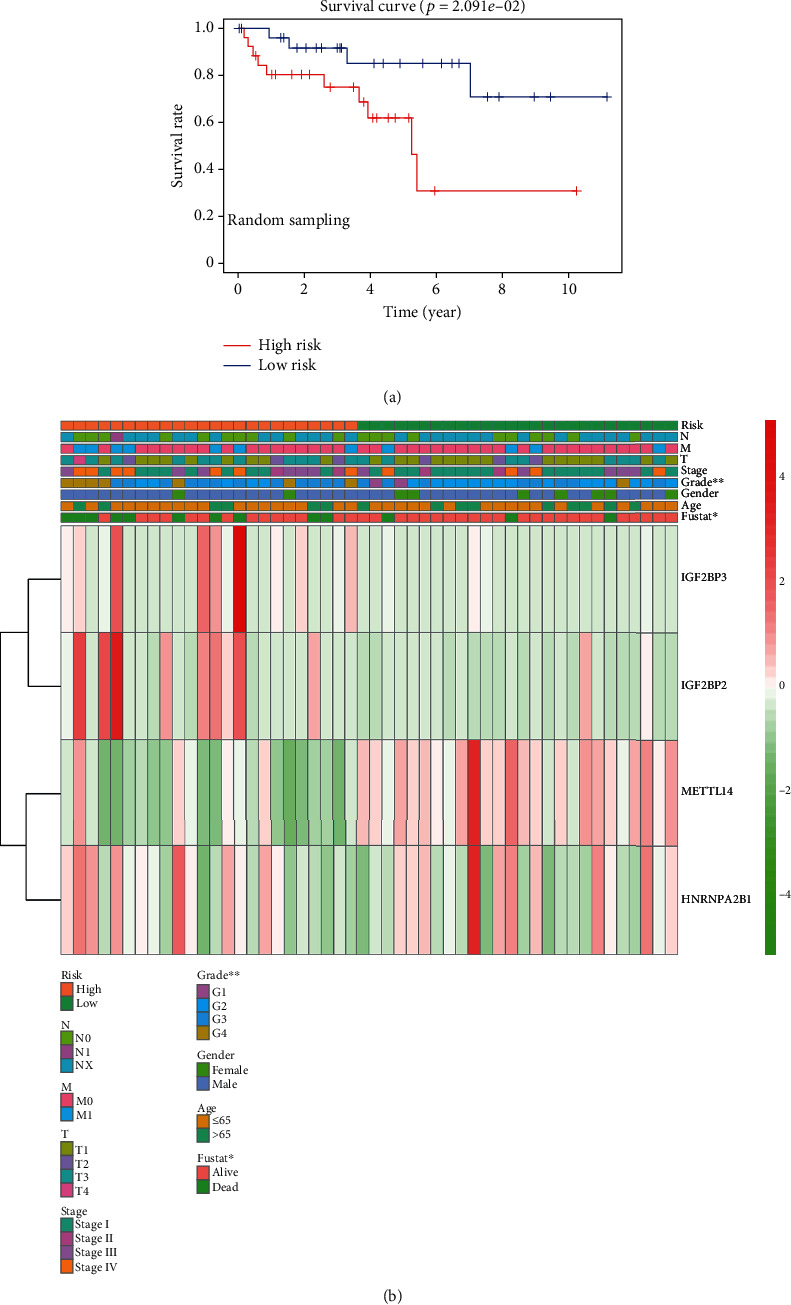
Random sampling of data in TCGA to validate the accuracy of the signature. (a) Kaplan-Meier overall survival (OS) rate curve of high-risk (red) and low-risk (blue) patients with ccRCC. Data was obtained by random sampling from TCGA. (b) Heat map of clinicopathological features and expression levels of IGF2BP3, IGF2BP2, HNRNPA2B1, and METTL14 genes in the randomly sampled data.

**Figure 8:**
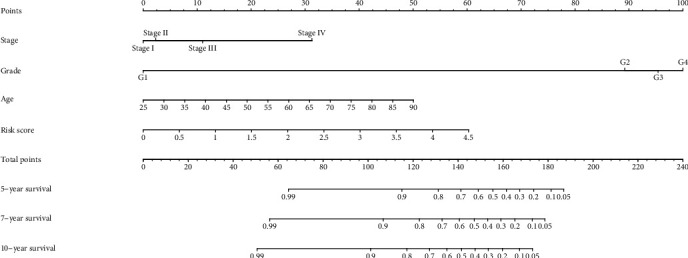
Nomogram to predict 5-year, 7-year, and 10-year OS of ccRCC patients.

To verify the accuracy of the signature, we tested it over randomly sampled data from TCGA. Survival curves indicated that the OS of the high-risk group was lower than the low-risk group (Figure 7(a)).  Figure 7(b) shows the expression levels of the four genes in the chosen samples. The high-risk group contained 24 samples, and the low-risk group contained 26 samples. Compared with the low-risk group, the high-risk group had lower expression of METTL14 and higher expression of IGF2BP3, IGF2BP2, and HNRNPA2B1. To further extend the performance of the risk signature, we made a nomogram to take other clinicopathological factors into account. By using this nomogram, we could calculate the 5-year survival, 7-year survival, and 10-year survival of the patients (Figure 8). Then, we used GSE22541 for external verification. Since this dataset only contained patient DFS information and not OS information, we verified the DFS of the risk model and the four genes in ccRCC and drew the corresponding survival curve (Supplementary Materials Figure S1(A–E)). Surprisingly, we found that the external verification results also support the results obtained through the TCGA database in the early stage.

### 3.8. GSEA Pathway Analysis of the Four Genes

**Figure 9:**
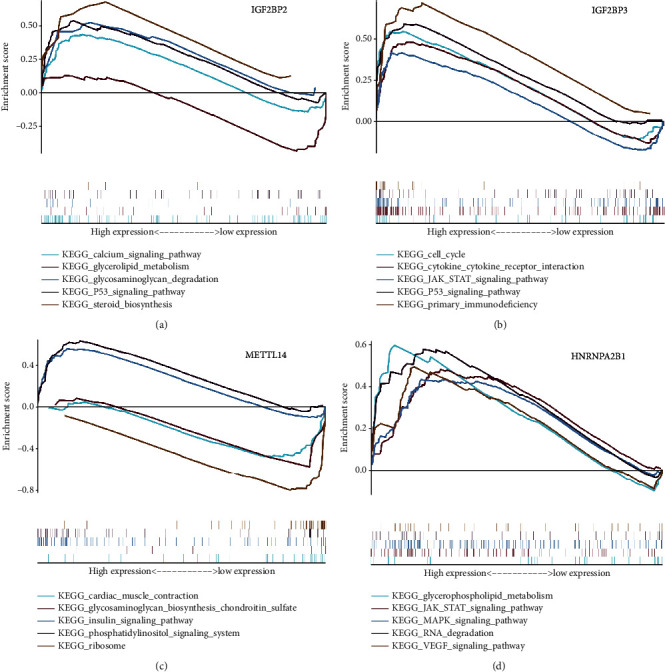
GSEA pathway analysis of IGF2BP3, IGF2BP2, HNRNPA2B1, and METTL14 genes. (a–d) An upward parabola indicates that the indicated gene promotes the pointed pathway; otherwise, the pathway is suppressed.

**Figure 10:**
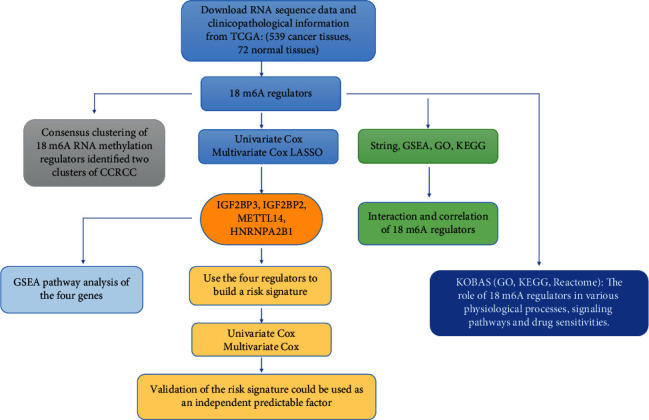
The flowchart of this study.

We selected five signaling pathways for evaluating changes in the expression of the four genes belonging to the newly described signature. An open-up parabola indicates that the gene activates the pathway, whereas an open-down parabola indicates that the gene can inhibit the pathway (Figures 9(a)–9(d)). For instance, IGF2BP3 has a positive regulatory effect on the calcium signaling pathway, glycosaminoglycan degradation, P53 signaling pathway, and steroid biosynthesis; however, high levels of IGF2BP3 can inhibit glycerolipid metabolism. Finally, in order to show the process of this research more clearly, we draw a corresponding flow chart (Figure 10).

## 4. Discussion

Evidence shows that m^6^A RNA methylation has various functions in the occurrence, development, and proliferation of cancer [26, 27]. It may also affect cancer stem cell pluripotency and cell differentiation [16, 28], promote cancer cell migration [29], and contribute to tumor immunity [30]. m^6^A RNA methylation regulators include three major elements: writers, erasers, and readers. Writers catalyze the formation of m^6^A, erasers remove m^6^A from RNAs, and readers recognize and bind m^6^A sites. As writers, the complex formed by METTL14 and METTL3 recognizes the substrate [31], WTAP ensures that the complex is located exactly on the nuclear speckle [32], RBM15 attaches to the WTAP-METTL3 complex and engages it to specific RNA sites [33], ZC3H13 mediates the combination of WTAP and Spenito [34], and KIAA1429 is related to the selectivity of m^6^A modified sites [35]. As erasers, FTO controls mRNA splicing and regulates adipogenesis [36], and ALKBH5 participates in the process of splicing and the production of longer 3′-UTR mRNAs [37]. Finally, as readers, YTH domain family members are the first to recognize m^6^A [38], IGF2BPs bind to m^6^A and enhance RNA stability of the target mRNA [23], HNRNPA2B1 mediates the splicing of RNAs and enhances primary miRNA processing [39], and HNRNPC and RBMX regulate the processing of m^6^A-containing RNA transcripts indirectly [40] [41].

In the United States, the estimated number of new patients with kidney and renal pelvis cancer in 2019 was 73,820 (44,120 males and 29,700 females), whereas the estimated death toll was 14,770 (9,820 males and 4,950 females) [1]. Compared with data from previous years, morbidity and mortality have increased. In China, the number of new patients with renal cancer in 2014 was about 6.8 × 10^4^ (4.3 × 10^4^ males and 2.6 × 10^4^ females) and the estimated death toll was 2.6 × 10^4^ (1.6 × 10^4^ males and 0.9 × 10^4^ females) [42]. At present, the main treatment for kidney cancer is surgery, and an adjuvant therapy, including immunotherapy and chemotherapy, can be chosen according to the disease stage. However, there is a possibility of recurrence to the surgical treatment and some patients are initially refractory to immunotherapy and chemotherapy [43]. Among renal cancers, ccRCC is the main histological subtype, accounting for 75% of all cases [44]. However, compared with other cancers, there are few studies on ccRCC. In addition, there are few articles on bioinformatics analyses of ccRCC. In this study, we analyzed a dataset of ccRCC patients from TCGA, grouped the data by consensus clustering, and built a risk signature with m^6^A RNA methylation regulators to predict the prognosis of patients with ccRCC. We hope that this can suggest ideas for future research.

Considering the close relationship between m^6^A and cancer, we wanted to explore the linkage between m^6^A RNA methylation regulators and ccRCC. In this study, 18 m^6^A RNA methylation regulators were chosen. To better understand the important role of m^6^A RNA methylation regulators in ccRCC, we first compared the expression of these regulators in normal and tumor tissues and found that most of them are differentially expressed among both kinds of tissues. Besides, correlation analyses revealed that these 18 regulators interact with each other. Therefore, it is suggested that these 18 m^6^A RNA methylation regulators could either act independently or interactively to play a role in the occurrence and development of ccRCC. To further determine the effects of m^6^A RNA methylation regulators on the clinicopathological characteristics and prognosis of the patients, we separated our data into two groups by consistent clustering. The expression levels of m^6^A RNA methylation regulators in the two clusters were different, and most of the regulators had a higher expression in cluster 2. Moreover, a survival curve showed that cluster 2 had a significantly worse prognosis that cluster 1.

Next, we tried to determine the function of these regulators in ccRCC by integrating their functions in GO, KEGG, and Reactome. We found that they can play roles in DNA repair, RNA splicing, and other physiological processes such as apoptosis, cell cycle, and epithelial mesenchymal transformation (EMT), and even inhibit or activate cell signaling pathways, including the PI3K/Akt pathway. Therefore, we proposed that these regulators affect the occurrence and development of ccRCC by intervening in the above processes. We also determined the drug sensitivities of these regulators, aiming to provide some ideas for future targeted drug research for ccRCC.

To build a risk signature, we used the LASSO Cox regression algorithm over the 18 regulators in the TCGA dataset. We then chose four regulators (IGF2BP3, IGF2BP2, METTL14, and HNRNPA2B1) to build the signature, and separated patients into high-risk and low-risk groups according to it. Characteristically, patients from the high-risk group had a worse prognosis, having increased expression levels of IGF2BP3, IGF2BP2, and HNRNPA2B1 and decreased expression levels of METTL14 compared to those in normal tissues.

The risk signature can be used independently or combined with other indicators to predict patient prognosis. To determine this, the signature was tested against randomly sampled data from TCGA. In these random samples, the prognosis predicted by the signature was found to be in accordance with the actual prognosis of the patients, and the expression levels of the four chosen genes were also consistent with previous results. All these results show that the risk signature can effectively judge the prognosis of patients with ccRCC. We believe that this risk signature can be used to predict the five-year survival rate of patients in the clinical practice. Finally, we enriched the function of the four signature genes in five different pathways; similar to previous results, we found that in patients with ccRCC, these genes play a positive or negative role in many physiological processes.

According to other studies, IGF2BP2 and IGF2BP3 belong to the IGF2BP protein family, formed by IGF2BP1-3. As readers, IGF2BPs recognize GGC sequences and target thousands of mRNA transcripts; they can regulate the stability, translation, and storage of RNA, thereby affecting the expression of genes (recognition of RNA N^6^-methyladenosine by IGF2BP proteins enhances mRNA stability and translation). Huang et al. found that in pancreatic ductal adenocarcinoma, the expression of IGF2BP2 was upregulated and led to a poor outcome [45]. In addition, in patients with acute myelocytic leukemia, the overexpression of IGF2BP2 indicates poor survival, and IGF2BP2 expression is associated with mutations in FLT3-ITD and IDH1, which are also indicators of poor prognosis [46]. These results are consistent with our results, that is, IGF2BP2 and IGF2BP3 play a positive regulatory role in the process of tumor occurrence and development. These conclusions are urging us to carry out relevant research to verify whether inhibiting the expression of IGF2BP2 and IGF2BP3 can inhibit the growth of the tumor.

HNRNPA2B1 binds m^6^A-containing sites on nuclear RNAs. HNRNPA2B1 can also regulate alternative splicing of exons in a set of transcripts, similar to METTL3; consequently, METTL3 depletion together with a diminishment in HNRNPA2B1 concentration may have a close correlated impact in the cell [47]. Previous studies have shown that HNRNPA2B1 is overexpressed in breast cancer tissue, and that its encoded protein can activate the STAT3 and ERK1/2 signaling pathways, thereby promoting the tumorigenic potential of cancer cells. Here, we found that the expression of HNRNPA2B1 in the high-risk group was also significantly increased, and that the prognosis of the group with high levels of this regulator is worse than that of the group with low levels. Therefore, studying the pathways related to this reader and finding possible inhibitors could also be a breach in the treatment of ccRCC.

As a writer, METTL14 plays a role by tightly combining with METTL3. Studies have revealed that METTL14 and ALKBH5 control the expression of each other and inhibit the expression of YTHDF3, thereby blocking RNA demethylation to degrade cancer cells [48]. Compared with studies focused in METTL3, research on METTL14 has only been gradually carried out in the last ten years. However, many articles have reported that METTL14 can mediate the self-renewal of HSCs (hematopoietic stem cells) by upregulating the expression of regulators such as BMI-1 and PRDM16 [49]. A study on leukemia also found that METTL14 can block myeloid differentiation and promote the self-renewal of normal HSPCs and LSCs/LICs (leukemia stem cells/leukemia-induced cells) [50]. In addition, downregulation of METTL14 can promote metastasis of liver cancer cells, whereas its overexpression significantly reduces tumor invasion and metastasis (METTL14 suppresses the metastatic potential of hepatocellular carcinoma by modulating N^6^-methyladenosine-dependent primary microRNA processing). These data are consistent with our results that suggest that a high expression of METTL14 can inhibit tumor growth or other harmful physiological processes. Therefore, improving the expression of METTL14 could be an effective therapeutic strategy to treat some diseases.

Although there are few studies on ccRCC and m^6^A, a high expression of IGF2BP2 and IGF2BP3 has been reported in many kinds of tumors. Consequently, it is thought that IGF2BP2 and IGF2BP3 are closely related to the occurrence and development of tumors. In addition, there are few studies about HNRNPA2B1, but according to our results and those from breast cancer studies, we believe that it is also an important regulator that promotes tumorigenesis. METTL14 may inhibit tumor development and metastasis. Compared with normal tissues, its expression is significantly reduced in tumor tissues; therefore, invasion and metastasis of the tumor are more likely to occur. In the future, we will further explore the relationship between these regulators and the occurrence and development of ccRCC, trying to identify the specific mechanisms that underlie this disease.

This study has some limitations. For example, it only discusses data at the gene and mRNA levels. Overcoming technical problems around the complexity of protein expression modification is needed to further analyze the relation between the selected m^6^A regulators and ccRCC at the protein level. Additionally, the AUC value of the ROC curve just exceeded 0.7; the sample size needs to be increased in the future to further confirm the sensitivity and specificity of this signature. However, we believe that the establishment of this signature will play a great role in predicting the five-year survival rate of patients with ccRCC and improving their treatment. This signature may also be a good starting point for new studies on ccRCC.

## 5. Conclusion

m^6^A RNA methylation regulators are closely related to the occurrence and development of ccRCC. The newly defined risk signature can predict the prognosis of patients with ccRCC. Regulators used to build the risk signature may also become targets for the diagnosis and treatment of ccRCC.

## Data Availability

All data included in this study are available upon request by contact with the corresponding author.

## Abbreviations

RCC: Renal cell carcinoma
ccRCC: Clear cell renal cell carcinoma
TCGA: The Cancer Genome Atlas
ALKBH5: ALKB homolog 5, RNA demethylase
RBM15B: RNA-binding motif protein 15B
IGF2BP2: Insulin-like growth factor 2 mRNA-binding protein 2
HNRNPA2B1: Heterogeneous nuclear ribonucleoprotein A2/B1
YTHDF2: YTH N^6^-methyladenosine RNA-binding protein 2
METTL4: Methyltransferase-like 4
ZC3H13: Zinc finger CCCH-type-containing 13
YTHDF3: YTH N^6^-methyladenosine RNA-binding protein 3
IGF2BP3: Insulin-like growth factor 2 mRNA-binding protein 3
RBMX: X-linked RNA-binding motif protein
FTO: FTO alpha-ketoglutarate-dependent dioxygenase
WTAP: WT1-associated protein
RBM15: RNA-binding motif protein 15
YTHDC1: YTH domain-containing 1
METTL14: Methyltransferase-like 14
IGF2BP1: Insulin-like growth factor 2 mRNA-binding protein 1
OS: Overall survival
DFS: Disease-free survival.

## Supplementary Materials

Supplementary Materials Figure S1: external verification based on the GSE22541 dataset. Table S1: expression of 18 m^6^A RNA methylation regulators between clusters 1 and 2.

## References

[1] Siegel R. L., Miller K. D., Jemal A. (2019). Cancer statistics, 2019. *CA: a Cancer Journal for Clinicians*.

[2] Leibovich B. C., Lohse C. M., Crispen P. L. (2010). Histological subtype is an independent predictor of outcome for patients with renal cell carcinoma. *The Journal of Urology*.

[3] Harshman L. C., Choueiri T. K., Drake C., Stephen Hodi F. (2014). Subverting the B7-H1/PD-1 pathway in advanced melanoma and kidney cancer. *Cancer Journal*.

[4] Desrosiers R., Friderici K., Rottman F. (1974). Identification of methylated nucleosides in messenger RNA from Novikoff hepatoma cells. *Proceedings of the National Academy of Sciences of the United States of America*.

[5] Ke S., Alemu E. A., Mertens C. (2015). A majority of m6A residues are in the last exons, allowing the potential for 3' UTR regulation. *Genes & Development*.

[6] Meyer K. D., Saletore Y., Zumbo P., Elemento O., Mason C. E., Jaffrey S. R. (2012). Comprehensive analysis of mRNA methylation reveals enrichment in 3′ UTRs and near stop codons. *Cell*.

[7] Csepany T., Lin A., Baldick C. J., Beemon K. (1990). Sequence specificity of mRNA N6-adenosine methyltransferase. *The Journal of Biological Chemistry*.

[8] Wei C. M., Moss B. (1977). Nucleotide sequences at the N^6^-methyladenosine sites of HeLa cell messenger ribonucleic acid. *Biochemistry*.

[9] Meyer K. D., Patil D. P., Zhou J. (2015). 5′ UTR m^6^A promotes cap-independent translation. *Cell*.

[10] Alarcon C. R., Lee H., Goodarzi H., Halberg N., Tavazoie S. F. (2015). *N*
^6^-methyladenosine marks primary microRNAs for processing. *Nature*.

[11] Patil D. P., Chen C. K., Pickering B. F. (2016). m^6^A RNA methylation promotes *XIST*-mediated transcriptional repression. *Nature*.

[12] Vu L. P., Pickering B. F., Cheng Y. (2017). The *N*^6^-methyladenosine (m^6^A)-forming enzyme METTL3 controls myeloid differentiation of normal hematopoietic and leukemia cells. *Nature Medicine*.

[13] Chen M., Wei L., Law C. T. (2018). RNA N6-methyladenosine methyltransferase-like 3 promotes liver cancer progression through YTHDF2-dependent posttranscriptional silencing of SOCS2. *Hepatology*.

[14] Liu J., Ren D., Du Z., Wang H., Zhang H., Jin Y. (2018). m^6^A demethylase FTO facilitates tumor progression in lung squamous cell carcinoma by regulating MZF1 expression. *Biochemical and Biophysical Research Communications*.

[15] Su R., Dong L., Li C. (2018). R-2HG exhibits anti-tumor activity by targeting FTO/m^6^A/MYC/CEBPA signaling. *Cell*.

[16] Zhang C., Samanta D., Lu H. (2016). Hypoxia induces the breast cancer stem cell phenotype by HIF-dependent and ALKBH5-mediated m6A-demethylation of NANOG mRNA. *Proceedings of the National Academy of Sciences of the United States of America*.

[17] Cui Q., Shi H., Ye P. (2017). m^6^A RNA methylation regulates the self-renewal and tumorigenesis of glioblastoma stem cells. *Cell Reports*.

[18] Zhang J., Tsoi H., Li X. (2016). Carbonic anhydrase IV inhibits colon cancer development by inhibiting the Wnt signalling pathway through targeting the WTAP-WT1-TBL1 axis. *Gut*.

[19] Bokar J. A., Shambaugh M. E., Polayes D., Matera A. G., Rottman F. M. (1997). Purification and cDNA cloning of the AdoMet-binding subunit of the human mRNA (N6-adenosine)-methyltransferase. *RNA*.

[20] Dai D., Wang H., Zhu L., Jin H., Wang X. (2018). N6-Methyladenosine links RNA metabolism to cancer progression. *Cell Death & Disease*.

[21] Batista P. J. (2017). The RNA modification *N*^6^-methyladenosine and its implications in human disease. *Genomics, Proteomics & Bioinformatics*.

[22] Xu C., Liu K., Ahmed H., Loppnau P., Schapira M., Min J. (2015). Structural basis for the discriminative recognition of *N*^6^-methyladenosine RNA by the human YT521-B homology domain family of proteins. *The Journal of Biological Chemistry*.

[23] Huang H., Weng H., Sun W. (2018). Recognition of RNA *N*^6^-methyladenosine by IGF2BP proteins enhances mRNA stability and translation. *Nature Cell Biology*.

[24] Zhao B. S., Roundtree I. A., He C. (2017). Post-transcriptional gene regulation by mRNA modifications. *Nature Reviews. Molecular Cell Biology*.

[25] Nagy A., Lanczky A., Menyhart O., Gyorffy B. (2018). Validation of miRNA prognostic power in hepatocellular carcinoma using expression data of independent datasets. *Scientific reports*.

[26] Chen J., Sun Y., Xu X. (2017). YTH domain family 2 orchestrates epithelial-mesenchymal transition/proliferation dichotomy in pancreatic cancer cells. *Cell Cycle*.

[27] Cai X., Wang X., Cao C. (2018). HBXIP-elevated methyltransferase METTL3 promotes the progression of breast cancer *via* inhibiting tumor suppressor let-7g. *Cancer Letters*.

[28] Bansal H., Yihua Q., Iyer S. P. (2014). WTAP is a novel oncogenic protein in acute myeloid leukemia. *Leukemia*.

[29] Yang L., Ma Y., Han W. (2015). Proteinase-activated receptor 2 promotes cancer cell migration through RNA methylation-mediated repression of miR-125b. *The Journal of Biological Chemistry*.

[30] Li H. B., Tong J., Zhu S. (2017). m^6^A mRNA methylation controls T cell homeostasis by targeting the IL-7/STAT5/SOCS pathways. *Nature*.

[31] Wang P., Doxtader K. A., Nam Y. (2016). Structural basis for cooperative function of Mettl3 and Mettl14 methyltransferases. *Molecular Cell*.

[32] Ping X. L., Sun B. F., Wang L. (2014). Mammalian WTAP is a regulatory subunit of the RNA N6-methyladenosine methyltransferase. *Cell Research*.

[33] Zhang S. (2018). Mechanism of *N*^6^-methyladenosine modification and its emerging role in cancer. *Pharmacology & Therapeutics*.

[34] Knuckles P., Lence T., Haussmann I. U. (2018). Zc3h13/Flacc is required for adenosine methylation by bridging the mRNA-binding factor Rbm15/Spenito to the m6A machinery component Wtap/Fl(2)d. *Genes & Development*.

[35] Schwartz S., Mumbach M. R., Jovanovic M. (2014). Perturbation of m6A writers reveals two distinct classes of mRNA methylation at internal and 5′ sites. *Cell Reports*.

[36] Zhao X., Yang Y., Sun B. F. (2014). FTO-dependent demethylation of N6-methyladenosine regulates mRNA splicing and is required for adipogenesis. *Cell Research*.

[37] Tang C., Klukovich R., Peng H. (2018). ALKBH5-dependent m6A demethylation controls splicing and stability of long 3′-UTR mRNAs in male germ cells. *Proceedings of the National Academy of Sciences of the United States of America*.

[38] Liao S., Sun H., Xu C. (2018). YTH domain: a family of *N*^6^-methyladenosine (m^6^A) readers. *Genomics, Proteomics & Bioinformatics*.

[39] Alarcon C. R., Goodarzi H., Lee H., Liu X., Tavazoie S., Tavazoie S. F. (2015). HNRNPA2B1 is a mediator of m^6^A-dependent nuclear RNA processing events. *Cell*.

[40] Liu N., Zhou K. I., Parisien M., Dai Q., Diatchenko L., Pan T. (2017). N6-Methyladenosine alters RNA structure to regulate binding of a low-complexity protein. *Nucleic Acids Research*.

[41] Liu N., Dai Q., Zheng G., He C., Parisien M., Pan T. (2015). *N*
^6^-Methyladenosine-dependent RNA structural switches regulate RNA-protein interactions. *Nature*.

[42] Chen W., Sun K., Zheng R. (2018). Cancer incidence and mortality in China, 2014. *Chinese Journal of Cancer Research*.

[43] Tamura K., Horikawa M., Sato S., Miyake H., Setou M. (2019). Discovery of lipid biomarkers correlated with disease progression in clear cell renal cell carcinoma using desorption electrospray ionization imaging mass spectrometry. *Oncotarget*.

[44] Lopez-Beltran A., Scarpelli M., Montironi R., Kirkali Z. (2006). 2004 WHO classification of the renal tumors of the adults. *European Urology*.

[45] Huang S., Wu Z., Cheng Y., Wei W., Hao L. (2019). Insulin-like growth factor 2 mRNA binding protein 2 promotes aerobic glycolysis and cell proliferation in pancreatic ductal adenocarcinoma via stabilizing *GLUT1* mRNA. *Acta Biochimica et Biophysica Sinica*.

[46] He X., Li W., Liang X. (2018). IGF2BP2 overexpression indicates poor survival in patients with acute myelocytic leukemia. *Cellular Physiology and Biochemistry*.

[47] Chen X. Y., Zhang J., Zhu J. S. (2019). The role of m^6^A RNA methylation in human cancer. *Molecular Cancer*.

[48] Panneerdoss S., Eedunuri V. K., Yadav P. (2018). Cross-talk among writers, readers, and erasers of m^6^A regulates cancer growth and progression. *Science Advances*.

[49] Yao Q. J., Sang L., Lin M. (2018). Mettl3-Mettl14 methyltransferase complex regulates the quiescence of adult hematopoietic stem cells. *Cell Research*.

[50] Weng H., Huang H., Wu H. (2018). METTL14 inhibits hematopoietic stem/progenitor differentiation and promotes leukemogenesis via mRNA m^6^A modification. *Cell Stem Cell*.

