# Supplementary material for: Identification of a New Prognostic Risk Signature of Clear Cell Renal Cell Carcinoma Based on N6-Methyladenosine RNA Methylation Regulators
Source: J Immunol Res. 2021 Feb 12;2021:6617841. doi: 10.1155/2021/6617841 (PMC7895564; doi:10.1155/2021/6617841)
Supplement: Supplementary Materials — Figure S1: external verification based on the GSE22541 dataset. Table S1: expression of 18 m6A RNA methylation regulators between clusters 1 and 2. [file 6617841.f1.pdf]

Supplementary materials

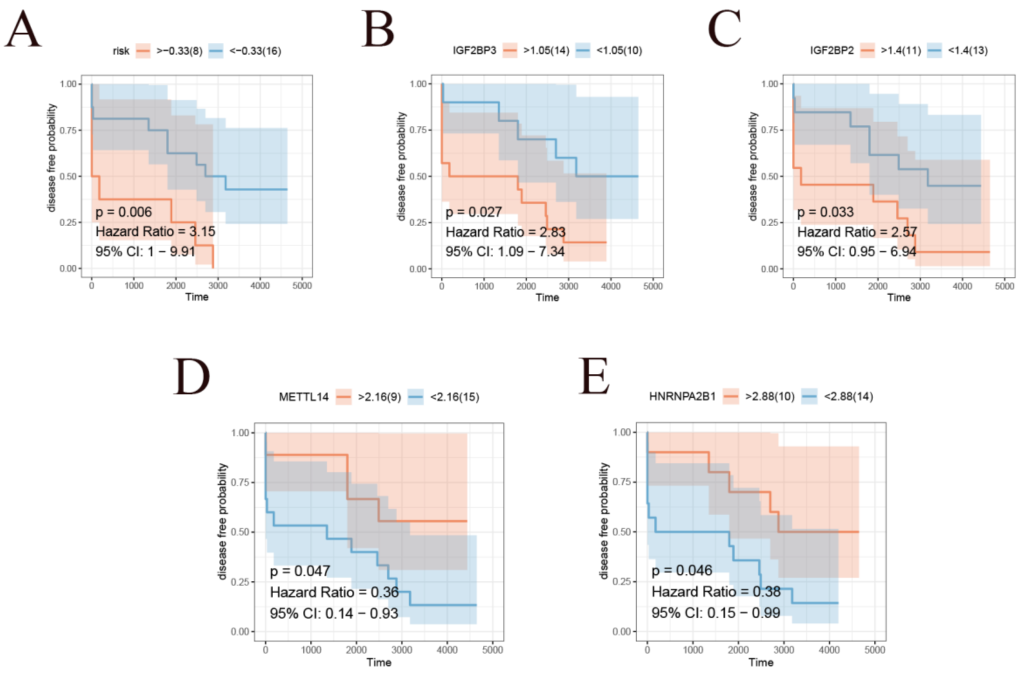

Figure S1: External verification based on the GSE22541 data set. DFS curve of ccRCC patients for the following factors: (A) Risk model; (B) IGF2BP3; (C) IGF2BP2; (D) METTL14; (E) HNRNPA2B1. The corresponding *P* values are shown in the graph.

Table S1

id	Cluster	ALKBH5	KIAA1429	RBM15B	IGF2BP2	HNRNPA2B1
TCGA-3Z-A93Z-01A-11R-A37O-07	1	48.524387	4.4757061	8.4600472	0.48213404	67.9260628
TCGA-6D-AA2E-01A-11R-A37O-07	2	40.263207	3.4361205	10.402739	1.0878436	79.5256459
TCGA-A3-3306-01A-01R-0864-07	1	47.080077	6.6158352	5.3507953	0.17545998	46.7410302
TCGA-A3-3307-01A-01R-0864-07	2	48.35366	5.9832176	7.421214	0.10389617	70.7815916
TCGA-A3-3308-01A-02R-1325-07	2	50.496035	8.0340453	7.495835	0.48138781	75.6415141
TCGA-A3-3311-01A-02R-1325-07	1	40.544402	6.2669107	5.8786208	1.49793096	60.0020406
TCGA-A3-3313-01A-02R-1325-07	1	42.569831	6.3162739	9.7066493	0.37871781	63.6191007
TCGA-A3-3316-01A-01R-0864-07	2	29.725711	8.6908327	6.6170721	1.06815266	86.2153214
TCGA-A3-3317-01A-02R-1325-07	2	49.054079	7.0918797	8.2658286	0.8584838	74.1318433
TCGA-A3-3319-01A-02R-1325-07	1	49.773011	6.2874933	3.9240507	3.1811983	55.84874
TCGA-A3-3320-01A-02R-1325-07	2	57.505406	7.4683539	7.131238	0.1541497	83.3458138
TCGA-A3-3322-01A-02R-1325-07	1	41.001485	6.8248979	5.8912402	0.26914223	68.527321
TCGA-A3-3323-01A-02R-1325-07	1	30.840947	8.5861485	6.4858211	0.76801314	60.2246435
TCGA-A3-3324-01A-02R-1325-07	1	28.443607	6.9382683	5.8144304	0.09108028	62.179157
TCGA-A3-3325-01A-01R-0864-07	2	68.394206	5.3332369	8.5560954	0.19744416	93.9488254
TCGA-A3-3326-01A-01R-0864-07	2	40.338364	6.0676736	6.0830256	0.36628036	85.5498267
TCGA-A3-3328-01A-01R-0864-07	2	49.39115	4.7491287	9.5704264	2.927084	82.2689818
TCGA-A3-3329-01A-01R-0864-07	1	41.657542	3.2400419	6.4164673	0.14049564	63.8097395
TCGA-A3-3331-01A-02R-1325-07	1	49.796715	6.6751708	6.4923003	0.14636343	66.0128088
TCGA-A3-3335-01A-01R-0864-07	1	45.98788	4.0348187	2.9431897	0.1864435	42.0500139
TCGA-A3-3343-01A-01R-0864-07	1	48.990735	5.7936356	5.7359169	0.41827488	54.3238896
TCGA-A3-3346-01A-01R-1766-07	1	41.922442	3.7135521	4.8088983	0.42044539	66.2194988
TCGA-A3-3347-01A-02R-1325-07	2	39.859096	6.2645216	7.7293959	1.84855412	86.6223954
TCGA-A3-3349-01A-01R-1188-07	2	39.732709	7.0640902	8.0062767	0.11504361	74.0625124
TCGA-A3-3351-01A-02R-1325-07	2	35.50008	8.1291496	6.5505382	0.18227156	83.8689224
TCGA-A3-3352-01A-01R-0864-07	2	44.072999	6.5778923	5.5112911	0.22692917	82.2013158
TCGA-A3-3357-01A-02R-1420-07	1	41.416401	5.8327685	4.4955679	0.109631	61.8865841
TCGA-A3-3358-01A-01R-1541-07	1	36.692735	4.8283323	4.6240833	1.24956453	46.1002283
TCGA-A3-3359-01A-01R-0864-07	2	54.863276	6.0721057	6.7776498	0.10984173	76.3149864
TCGA-A3-3362-01A-02R-1325-07	2	46.172201	6.0968357	7.1895037	0.86966403	71.5715825
TCGA-A3-3363-01A-01R-0864-07	1	36.992525	3.6547381	8.1353041	9.78633052	51.2630224
TCGA-A3-3365-01A-01R-0864-07	2	44.57009	5.4060241	8.1200316	0.13099782	65.1577332
TCGA-A3-3367-01A-02R-1420-07	1	41.126984	8.0136104	5.0549115	0.21890195	64.6352559
TCGA-A3-3370-01A-02R-1420-07	2	46.936783	6.8732397	6.6642667	0.2533866	78.8543497
TCGA-A3-3372-01A-02R-1325-07	2	43.959059	6.2947217	8.4063199	0.13344824	72.1325659
TCGA-A3-3373-01A-02R-1420-07	1	47.982574	4.2602063	7.660114	0.25453677	54.6381112
TCGA-A3-3374-01A-02R-1325-07	1	25.109021	2.4538897	9.0202683	1.2671243	34.699866
TCGA-A3-3376-01A-02R-1420-07	1	38.100693	8.7879327	10.606214	0.20978079	69.1449205
TCGA-A3-3378-01A-02R-1325-07	1	46.746504	4.27568	5.8630397	0.36234042	71.8796273
TCGA-A3-3380-01A-01R-0864-07	1	44.730027	6.9041224	5.931054	0.13815983	67.1470338
TCGA-A3-3382-01A-02R-1325-07	1	42.530448	8.6700563	5.5655579	0.75492127	63.2336913
TCGA-A3-3383-01A-02R-1325-07	1	70.253396	3.7630605	5.8806636	0.15371616	66.3896467
TCGA-A3-3385-01A-02R-1420-07	1	40.223298	7.0570144	7.3294684	0.05007368	66.320801
TCGA-A3-3387-01A-01R-1541-07	1	40.425426	7.8770162	6.8065164	1.11458311	64.4551766
TCGA-A3-A6NI-01A-11R-A33J-07	1	43.93513	5.0969659	6.9608168	0.15896923	70.1018498
TCGA-A3-A6NJ-01A-12R-A33J-07	2	49.942825	5.0332988	8.9574607	0.39995034	75.9296354
TCGA-A3-A6NL-01A-11R-A33J-07	2	35.694543	6.2772437	8.1194376	0.12590389	74.5326555
TCGA-A3-A6NN-01A-12R-A33J-07	1	42.583232	4.9547734	8.5322093	0.16145265	67.5479587
TCGA-A3-A8CQ-01A-11R-A37O-07	2	56.261145	6.269834	10.5531	0.538703	93.2438901
TCGA-A3-A8OU-01A-11R-A37O-07	2	51.757637	4.5363519	7.1639402	0.35927313	79.1688168

TCGA-A3-A8OV-01A-11R-A37O-07	2	61.353576	5.1109002	6.0634128	0.11691034	78.2802179
TCGA-A3-A8OW-01A-11R-A37O-07	2	40.11695	5.6604459	8.4098388	0.18152495	93.4202625
TCGA-A3-A8OX-01A-11R-A37O-07	2	42.104769	4.3042935	10.689303	0.45036391	73.5279939
TCGA-AK-3425-01A-02R-1277-07	1	35.805112	6.0758943	6.2353834	2.21616783	49.733945
TCGA-AK-3426-01A-02R-1325-07	1	31.567663	2.5880933	5.4658654	0.44918343	65.5241452
TCGA-AK-3427-01A-01R-0864-07	1	23.200307	3.1631571	3.3968138	1.57468394	25.2954011
TCGA-AK-3428-01A-02R-1277-07	1	52.845211	9.7936913	6.7258416	0.45176982	43.0453537
TCGA-AK-3429-01A-02R-1325-07	1	34.939599	5.6668632	5.0683173	0.0916291	51.1689094
TCGA-AK-3431-01A-02R-1277-07	1	42.381136	7.7724312	7.886533	0.18912289	48.6812507
TCGA-AK-3433-01A-02R-1277-07	1	15.868661	5.4117687	5.341471	3.37472849	68.7587033
TCGA-AK-3434-01A-02R-1277-07	1	46.227813	5.3502568	5.5349884	0.42390127	57.9604766
TCGA-AK-3436-01A-02R-1325-07	1	25.487664	7.5686594	4.3796698	1.30621485	35.2315868
TCGA-AK-3440-01A-02R-1277-07	1	17.622262	3.3345933	9.1670714	5.97284074	66.6698881
TCGA-AK-3443-01A-02R-1325-07	2	23.332174	5.0601003	7.70684	4.61371162	91.2742063
TCGA-AK-3445-01A-02R-1277-07	1	39.542833	9.9095007	5.4161676	0.18107605	49.7746767
TCGA-AK-3447-01A-01R-1766-07	1	5.2587717	1.40434	1.4705322	0.59124194	17.2958151
TCGA-AK-3450-01A-02R-1277-07	1	50.356956	4.7244018	4.7369989	0.13019772	63.735612
TCGA-AK-3451-01A-02R-1188-07	1	24.595542	3.0073022	1.7105214	0.4845636	32.4511909
TCGA-AK-3453-01A-02R-1277-07	2	35.389593	3.8553923	5.9465657	0.34664432	81.8264431
TCGA-AK-3454-01A-02R-1277-07	2	29.20091	3.7356845	6.0164661	0.76351247	82.0662925
TCGA-AK-3455-01A-01R-0864-07	1	43.481805	5.0307485	3.9291354	0.07677512	65.2403534
TCGA-AK-3456-01A-02R-1325-07	1	18.638048	5.3830263	9.0257415	1.77091285	53.2482414
TCGA-AK-3458-01A-01R-1503-07	1	24.792241	3.4345804	2.1857749	0.37788897	37.041873
TCGA-AK-3460-01A-02R-1277-07	1	32.57752	6.2873286	5.1400479	0.14915366	58.8561501
TCGA-AK-3461-01A-02R-1277-07	2	40.367938	6.0372317	7.5087008	0.49335401	89.5614062
TCGA-AK-3465-01A-02R-1325-07	1	22.231156	4.9959145	8.8233538	3.77095384	74.4514305
TCGA-AS-3777-01A-01R-0864-07	1	13.007075	2.8532843	5.3285245	1.19781338	37.7853295
TCGA-AS-3778-01A-01R-A32Z-07	1	43.967954	5.7484018	7.1239196	0.18695192	60.6000881
TCGA-B0-4688-01A-01R-1277-07	2	34.47691	8.0710679	11.620751	6.21571068	111.585415
TCGA-B0-4690-01A-01R-1277-07	2	44.204573	6.8443663	7.4281248	7.03416116	93.0067603
TCGA-B0-4691-01A-01R-1277-07	2	50.055811	3.6753176	4.9143125	0.22186133	74.9865868
TCGA-B0-4693-01A-01R-1277-07	2	50.769556	5.5884665	6.6831936	0.08045844	72.1677056
TCGA-B0-4694-01A-01R-1277-07	1	31.951206	4.7990474	5.5145256	0.98911852	53.5884333
TCGA-B0-4696-01A-01R-1277-07	2	77.744234	11.892678	9.0004642	0.05764137	107.375768
TCGA-B0-4697-01A-01R-1277-07	2	45.342852	4.2906131	7.4994196	6.12376801	107.968261
TCGA-B0-4698-01A-01R-1503-07	2	23.739293	8.8032019	10.850325	26.6604948	134.90236
TCGA-B0-4699-01A-01R-1277-07	2	35.260237	6.1976781	9.8389221	11.5271104	95.3073281
TCGA-B0-4700-01A-02R-1541-07	1	45.219072	3.7681464	7.5116323	2.21304104	69.3855488
TCGA-B0-4701-01A-01R-1277-07	2	46.502114	4.9689026	5.1481079	1.09032856	83.1963232
TCGA-B0-4703-01A-01R-1277-07	2	42.364107	6.8302247	7.6207552	1.86923511	80.8128195
TCGA-B0-4706-01A-01R-1503-07	2	44.721653	4.7701266	6.405423	1.3072968	74.9012032
TCGA-B0-4707-01A-01R-1277-07	1	29.944977	2.9414556	5.9324726	0.94067523	57.5111
TCGA-B0-4710-01A-01R-1503-07	2	47.189071	5.908345	6.962738	0.54491659	97.8941882
TCGA-B0-4712-01A-01R-1503-07	1	30.08748	4.7947006	5.8944585	7.28713871	58.6315792
TCGA-B0-4713-01A-01R-1277-07	1	43.380923	4.634002	4.1598766	0.20678779	63.4742956
TCGA-B0-4714-01A-01R-1277-07	2	32.81752	3.9066937	5.0113647	0.36460919	106.823212
TCGA-B0-4718-01A-01R-1277-07	2	44.545782	3.4972797	5.4831441	0.4062373	86.1379941
TCGA-B0-4810-01A-01R-1503-07	2	62.338795	4.8943397	6.7273617	1.86369379	80.8487978
TCGA-B0-4811-01A-01R-1503-07	2	24.466491	3.2261411	4.7145383	0.78750206	109.225289
TCGA-B0-4813-01A-01R-1277-07	2	40.226134	2.4334158	6.7932536	0.2031859	96.1164586
TCGA-B0-4814-01A-01R-1277-07	1	38.732198	8.1712271	5.1058473	0.09237571	66.5745887
TCGA-B0-4815-01A-01R-1503-07	2	36.626008	6.0923964	6.3984373	0.7444364	105.15304

TCGA-B0-4816-01A-01R-1503-07	2	35.780929	4.8211334	7.5100487	0.29016031	78.3425145
TCGA-B0-4817-01A-01R-1277-07	1	26.830423	3.467028	3.7879383	0.15511572	62.2642601
TCGA-B0-4818-01A-01R-1503-07	2	57.381921	5.3692097	5.847698	0.10843458	90.1357581
TCGA-B0-4819-01A-01R-1277-07	2	28.881105	3.553405	6.474829	1.33861116	80.0851676
TCGA-B0-4821-01A-01R-1503-07	2	43.051937	2.809069	4.2947452	4.78113138	86.7709605
TCGA-B0-4822-01A-01R-1277-07	2	29.775909	2.7946123	5.5529864	0.66566273	83.934381
TCGA-B0-4823-01A-02R-1420-07	1	46.615256	7.0613805	6.9808553	0.09269075	43.7086464
TCGA-B0-4824-01A-01R-1277-07	2	50.275797	4.910497	4.8279052	0.27280069	85.7896492
TCGA-B0-4827-01A-02R-1420-07	1	85.814191	4.9764234	6.3461153	0.34233248	50.378648
TCGA-B0-4828-01A-01R-1277-07	2	50.922982	5.1225636	5.8778774	1.59672861	85.1224807
TCGA-B0-4833-01A-01R-1305-07	2	43.389099	4.1811914	6.2236862	0.23637344	77.2379931
TCGA-B0-4834-01A-01R-1305-07	1	29.480868	6.2485508	5.6413783	4.60498066	53.5633167
TCGA-B0-4836-01A-01R-1305-07	1	40.823418	5.6622511	5.6846285	0.57505093	58.4154099
TCGA-B0-4837-01A-01R-1305-07	1	42.714862	4.9129784	6.1368364	0.29782435	51.122259
TCGA-B0-4838-01A-01R-1305-07	2	53.77271	5.3210939	6.5427802	0.10511871	83.9939107
TCGA-B0-4839-01A-01R-1305-07	1	30.390804	4.1874799	6.558427	0.15815961	41.2153035
TCGA-B0-4841-01A-01R-1277-07	1	26.214835	3.2400665	4.159418	0.14601699	41.890942
TCGA-B0-4842-01A-02R-1420-07	1	66.065278	2.7323778	7.2939341	0.17703917	47.2322726
TCGA-B0-4843-01A-01R-1277-07	1	35.316077	3.8410499	6.5254391	0.83910991	68.2613066
TCGA-B0-4844-01A-01R-1277-07	1	49.607367	4.0344851	5.5562422	0.56583052	61.7012382
TCGA-B0-4845-01A-01R-1277-07	2	45.238917	3.9082753	7.5560771	0.10117095	102.762799
TCGA-B0-4846-01A-01R-1277-07	2	52.932713	5.613843	6.1348943	0.31778594	88.338965
TCGA-B0-4847-01A-01R-1277-07	2	36.665178	3.192325	4.4252524	0.20624609	87.907823
TCGA-B0-4848-01A-01R-1277-07	1	49.983353	5.6191899	6.8377658	1.47612524	63.7071587
TCGA-B0-4849-01A-01R-1277-07	2	49.681949	4.7412312	6.9547237	0.39651377	109.998847
TCGA-B0-4852-01A-01R-1503-07	2	39.255528	6.6692575	5.5622821	0.42729065	122.917576
TCGA-B0-4945-01A-01R-1420-07	2	52.064653	4.5551848	8.4514566	0.26166574	102.890217
TCGA-B0-5075-01A-01R-1334-07	1	43.484748	4.5766383	4.9895385	0.14633605	71.4648299
TCGA-B0-5077-01A-01R-1334-07	2	51.118773	6.3558963	6.1903706	0.28696861	79.491095
TCGA-B0-5080-01A-01R-1503-07	1	45.931091	6.4423821	10.471861	0.74188358	57.8055903
TCGA-B0-5081-01A-01R-1334-07	2	29.742126	3.9584886	6.4643755	2.37985297	86.1013164
TCGA-B0-5083-01A-02R-1420-07	1	23.326364	5.0293496	6.0835441	2.85805461	55.4324216
TCGA-B0-5084-01A-01R-1334-07	2	24.097239	5.1924867	8.3863073	16.9070269	85.4179024
TCGA-B0-5085-01A-01R-1334-07	1	36.833809	2.7399935	3.7503358	0.15609311	73.5424315
TCGA-B0-5088-01A-01R-1334-07	2	37.825776	4.1917856	5.7431476	0.22586857	77.0003377
TCGA-B0-5092-01A-01R-1420-07	2	39.281936	4.3005427	5.5280788	3.00072575	99.9692914
TCGA-B0-5094-01A-01R-1420-07	1	45.861171	6.0821479	5.0982689	0.23933346	62.7913652
TCGA-B0-5095-01A-01R-1420-07	2	42.443208	6.4873025	7.998285	0.48722503	74.9942097
TCGA-B0-5096-01A-01R-1420-07	1	41.384214	8.4617101	5.8248919	5.8389783	53.3235066
TCGA-B0-5097-01A-01R-1420-07	2	34.942413	8.1190018	8.524946	4.89350639	71.988488
TCGA-B0-5098-01A-01R-1420-07	2	48.705685	5.7581562	8.3483174	28.6563805	89.9611931
TCGA-B0-5099-01A-01R-1420-07	2	56.732148	5.1304406	7.8267913	0.16504526	71.810102
TCGA-B0-5100-01A-01R-1420-07	1	47.800773	4.5759657	9.9994377	5.29386111	58.6472481
TCGA-B0-5102-01A-01R-1420-07	1	72.037633	4.9996273	6.1704745	0.06307801	62.3964432
TCGA-B0-5104-01A-01R-1420-07	2	50.501475	4.6425723	6.4528771	0.27192525	83.2015038
TCGA-B0-5106-01A-01R-1420-07	1	28.638761	5.8237117	7.0793296	0.3200446	71.382536
TCGA-B0-5107-01A-01R-1420-07	2	52.711063	3.6095563	7.6043546	2.75222998	95.4592666
TCGA-B0-5108-01A-01R-1420-07	1	34.74856	7.8095445	7.4335058	0.72489174	69.8765152
TCGA-B0-5109-01A-02R-1420-07	2	51.570499	6.9619475	8.1394867	16.9439917	120.85056
TCGA-B0-5110-01A-01R-1420-07	2	43.083559	7.1497394	8.3534371	0.20534341	84.6507943
TCGA-B0-5113-01A-01R-1420-07	2	38.543469	8.4496625	6.955495	0.36691077	73.4317307
TCGA-B0-5115-01A-01R-1420-07	2	38.088323	6.9551575	5.5311456	0.22446428	77.5155815

TCGA-B0-5116-01A-02R-1420-07	1	45.248925	7.033518	7.0269983	7.09917502	44.7557085
TCGA-B0-5117-01A-01R-1420-07	1	16.151947	3.2590168	8.6047595	4.22997886	54.6810192
TCGA-B0-5119-01A-02R-1420-07	1	65.036032	7.2429436	6.9606134	0.17583752	61.307644
TCGA-B0-5120-01A-01R-1420-07	1	50.178694	8.1994464	7.3656701	0.24098839	60.0972485
TCGA-B0-5121-01A-02R-1420-07	1	35.889002	4.1094756	5.5058883	0.19462692	57.4746099
TCGA-B0-5399-01A-01R-1503-07	2	50.06798	5.9882536	6.5855301	0.0650453	72.7933301
TCGA-B0-5400-01A-01R-1503-07	1	59.038222	2.5482654	5.4404256	0.29916172	51.438286
TCGA-B0-5402-01A-01R-1503-07	1	40.728547	8.8323324	7.3890984	3.02113246	60.2093107
TCGA-B0-5690-01A-11R-1541-07	1	42.826631	6.2456481	10.977518	0.20382511	66.4463348
TCGA-B0-5691-01A-11R-1541-07	1	43.171535	8.0146624	9.6662752	1.46136232	60.1554515
TCGA-B0-5692-01A-11R-1541-07	1	53.443172	5.465523	6.7974171	0.05470105	60.3621014
TCGA-B0-5693-01A-11R-1541-07	2	53.889522	7.8194504	7.7806693	0.19754989	75.6555338
TCGA-B0-5694-01A-11R-1541-07	1	39.633807	6.3952188	5.8892443	0.57206856	58.9028778
TCGA-B0-5695-01A-11R-1541-07	2	59.410372	6.1867547	8.6630171	0.21336614	70.1815455
TCGA-B0-5696-01A-11R-1541-07	1	60.108236	4.0395321	5.6212625	0.15953083	61.009258
TCGA-B0-5697-01A-11R-1541-07	2	40.362285	4.3937068	10.440655	6.33013338	77.2223999
TCGA-B0-5698-01A-11R-1672-07	2	49.580838	5.4872458	7.4436567	0.56779961	74.1171473
TCGA-B0-5699-01A-11R-1541-07	1	55.839474	6.7597965	6.9001814	0.26523676	63.8519195
TCGA-B0-5700-01A-11R-1541-07	1	48.325549	4.0742313	8.4014676	0.15303048	47.6055784
TCGA-B0-5701-01A-11R-1541-07	1	46.605308	6.4977	6.0619826	0.33299595	52.6093466
TCGA-B0-5702-01A-11R-1541-07	1	11.580707	1.4125277	4.8902064	1.08088116	18.1024107
TCGA-B0-5703-01A-11R-1541-07	1	48.784413	4.9950325	7.7706174	0.07440782	57.384052
TCGA-B0-5705-01A-11R-1541-07	1	44.283232	7.2316714	6.1770189	0.09708581	60.3219699
TCGA-B0-5706-01A-11R-1541-07	1	52.43163	6.1771384	6.1763009	0.22103518	58.8924462
TCGA-B0-5707-01A-11R-1541-07	2	41.090158	3.3858903	16.34929	12.5598676	83.1972769
TCGA-B0-5709-01A-11R-1541-07	1	41.353001	7.1513433	7.9816396	1.84366211	64.3774759
TCGA-B0-5710-01A-11R-1672-07	1	39.034941	7.7082763	7.1369328	0.28139981	57.5324797
TCGA-B0-5711-01A-11R-1672-07	1	46.46593	7.9537246	5.9467684	0.09229294	55.7679954
TCGA-B0-5712-01A-11R-1672-07	1	54.187098	4.5149474	7.2383759	0.15556138	71.9243303
TCGA-B0-5713-01A-11R-1672-07	2	47.424634	6.9441764	7.0040852	0.10926003	80.7827915
TCGA-B0-5812-01A-11R-1672-07	2	46.742785	8.5486571	8.8888186	0.33008014	70.4237372
TCGA-B2-3923-01A-02R-1325-07	1	19.272851	5.9554555	8.4235046	6.06987005	72.1656884
TCGA-B2-3923-01A-02R-A277-07	1	24.96253	8.5486965	10.051004	4.1521343	32.0532852
TCGA-B2-3923-01B-10R-A277-07	2	18.013524	8.6566797	4.6356999	8.02831637	88.4344854
TCGA-B2-3924-01A-02R-1325-07	1	42.568119	7.0302166	6.9184105	0.10506289	59.6431097
TCGA-B2-3924-01A-02R-A277-07	1	54.347222	10.647769	7.3207102	0.20535095	49.5516516
TCGA-B2-3924-01B-03R-A277-07	2	33.323664	10.327444	6.6423605	0.16170732	129.323222
TCGA-B2-4098-01A-02R-1325-07	1	26.959592	4.6318321	6.6891223	3.73819227	62.0516242
TCGA-B2-4099-01A-02R-1188-07	1	35.946152	5.1188043	5.6887744	0.24982235	40.7232757
TCGA-B2-4101-01A-02R-1277-07	2	41.455597	7.3652372	7.6447541	0.41299671	77.2128182
TCGA-B2-4102-01A-02R-1325-07	2	38.783478	6.5647552	6.7103777	0.49572483	78.9865936
TCGA-B2-5633-01A-01R-1541-07	1	38.638254	8.5063057	8.3892559	0.3535914	53.6033029
TCGA-B2-5633-01A-01R-A277-07	1	36.255003	8.7496366	6.9635129	0.14544074	9.63589224
TCGA-B2-5633-01B-04R-A277-07	2	30.185193	10.814745	7.0523815	0.90745019	91.4339939
TCGA-B2-5635-01A-01R-1541-07	1	35.096748	6.0019984	8.2126772	0.19038945	67.2306563
TCGA-B2-5635-01A-01R-A277-07	1	32.401201	11.207261	7.3960277	0.13912064	17.4434942
TCGA-B2-5635-01B-04R-A277-07	2	19.002286	11.135287	5.0459875	0.38165281	91.4435399
TCGA-B2-5636-01A-02R-1541-07	1	63.865885	9.0981479	14.862433	0.33990683	57.5103919
TCGA-B2-5639-01A-01R-1541-07	1	50.392706	6.363525	5.715521	2.17454291	58.3969902
TCGA-B2-5641-01A-01R-1541-07	1	38.241537	7.9057691	6.9730005	0.60435539	46.021331
TCGA-B2-A4SR-01A-11R-A266-07	2	55.92329	6.7183921	7.3895952	0.09581659	100.816165
TCGA-B4-5377-01A-01R-1503-07	1	50.968623	6.3265698	7.8953835	0.140633	68.3665876

TCGA-B4-5378-01A-01R-1503-07	1	33.517196	8.450473	11.268907	0.27833132	64.2231216
TCGA-B4-5832-01A-11R-1672-07	2	13.020694	6.9408283	2.114629	0.64877745	86.3380292
TCGA-B4-5834-01A-11R-1672-07	1	38.706586	8.2880733	7.086954	0.12157743	61.8863726
TCGA-B4-5835-01A-11R-1672-07	1	20.52431	5.1208967	5.6834414	5.22161284	62.2047993
TCGA-B4-5836-01A-11R-1672-07	1	47.080669	5.9795859	5.3119568	0.13509875	60.1662453
TCGA-B4-5838-01A-11R-1672-07	1	48.311689	6.5349295	5.6596973	0.1899604	37.9019445
TCGA-B4-5843-01A-11R-1672-07	1	44.257269	7.8164052	6.9861042	0.26718707	61.1930057
TCGA-B4-5844-01A-11R-1672-07	1	59.585415	5.9509166	7.8786078	0.17074263	70.4249476
TCGA-B8-4143-01A-01R-1188-07	2	25.022934	6.0997831	7.9158323	8.99218019	94.6213208
TCGA-B8-4146-01B-11R-1672-07	2	21.4765	10.82007	2.6204921	0.3086155	105.184748
TCGA-B8-4148-01A-02R-1325-07	2	51.94107	5.85117	9.5611473	0.30712481	96.2917899
TCGA-B8-4151-01A-01R-1188-07	1	60.948366	4.9692371	5.1101943	0.10885818	45.0003161
TCGA-B8-4153-01B-11R-1672-07	1	48.76386	3.8564	6.5586327	0.08350475	53.8373441
TCGA-B8-4154-01A-01R-1188-07	1	38.067728	4.9392127	3.5249751	0.14134816	27.5917467
TCGA-B8-4619-01A-02R-1325-07	1	26.745694	5.9925117	7.2505493	3.98871054	68.771468
TCGA-B8-4620-01A-02R-1325-07	1	41.710872	7.316636	7.579544	2.17274871	57.9648663
TCGA-B8-4621-01A-01R-1503-07	1	30.939274	3.2034468	8.1620553	6.24488102	53.425667
TCGA-B8-4622-01A-02R-1277-07	1	46.390581	6.4572913	6.809064	0.41240241	68.0042633
TCGA-B8-5158-01A-01R-1420-07	1	49.821146	4.9205184	4.9524038	0.70737553	63.8754421
TCGA-B8-5159-01A-01R-1420-07	1	32.647035	8.4030443	4.8764307	0.12755952	51.1294044
TCGA-B8-5162-01A-01R-1420-07	1	38.79852	8.3268819	9.7293443	0.61147531	58.4855437
TCGA-B8-5163-01A-01R-1420-07	1	28.347786	8.4533454	6.2111306	0.22195976	65.9678568
TCGA-B8-5164-01A-01R-1420-07	1	43.926083	5.4840255	6.8887275	0.10982378	69.8213052
TCGA-B8-5165-01A-01R-1420-07	1	36.524807	10.065088	8.2272885	0.57912444	58.5430472
TCGA-B8-5545-01A-01R-1672-07	1	45.221229	6.02561	11.612947	0.79621848	64.657519
TCGA-B8-5546-01A-01R-1541-07	2	54.041164	5.5247723	13.653287	0.26270343	74.8969056
TCGA-B8-5549-01A-01R-1541-07	1	48.5782	7.2284939	6.910307	0.29227378	41.4412955
TCGA-B8-5550-01A-01R-1541-07	1	32.025459	6.3334784	7.282677	0.15391267	55.1327337
TCGA-B8-5551-01A-01R-1541-07	1	41.824547	5.9837603	7.8093774	2.57307983	71.3083098
TCGA-B8-5552-01B-11R-1672-07	1	45.400937	7.6253801	9.3225172	0.39735441	62.474982
TCGA-B8-5553-01A-01R-1541-07	1	43.65767	6.729882	8.2429396	0.48497741	53.6197469
TCGA-B8-A54D-01A-21R-A266-07	2	34.641788	4.0971231	5.2502994	0.10251125	99.0982056
TCGA-B8-A54E-01A-11R-A266-07	2	44.368878	3.9494636	7.3662898	4.28209384	81.128377
TCGA-B8-A54F-01A-11R-A266-07	2	28.609347	6.2160772	4.8008668	0.40400282	96.9773414
TCGA-B8-A54G-01A-11R-A266-07	2	42.749887	4.4281137	5.6672594	0.9404562	111.235303
TCGA-B8-A54H-01A-11R-A33J-07	2	40.136568	5.6701601	6.5278112	0.08697415	89.9318079
TCGA-B8-A54I-01A-21R-A33J-07	1	32.608587	3.2446122	4.114379	0.80026115	69.1945543
TCGA-B8-A54J-01A-11R-A33J-07	2	45.118085	4.4261827	7.8563913	0.18713346	95.7566076
TCGA-B8-A54K-01A-11R-A33J-07	1	35.914073	7.6127498	10.766029	0.52717246	65.1279275
TCGA-B8-A7U6-01A-12R-A37O-07	2	41.164432	4.4977184	10.552048	0.856569	102.48183
TCGA-B8-A8YJ-01A-13R-A39I-07	2	32.887492	4.8929927	11.763259	0.65286233	81.5737771
TCGA-BP-4158-01A-02R-1289-07	2	36.21581	5.88515	5.1782971	0.143265	79.4363281
TCGA-BP-4159-01A-02R-1289-07	1	31.648098	4.9614212	5.7047283	0.24219001	55.3677789
TCGA-BP-4160-01A-02R-1289-07	1	41.71419	7.6846119	6.0974066	0.15559648	54.711528
TCGA-BP-4161-01A-02R-1325-07	2	45.522532	5.6766388	5.3900177	0.48602969	70.7904189
TCGA-BP-4162-01A-02R-1325-07	2	43.753133	6.0100864	5.9955014	0.23466688	83.8543837
TCGA-BP-4163-01A-02R-1325-07	1	40.621094	7.0214399	6.0256558	2.50533212	67.5722197
TCGA-BP-4164-01A-02R-1325-07	2	51.730575	7.1822343	4.7234445	0.09292201	79.8891396
TCGA-BP-4165-01A-02R-1289-07	2	59.143405	4.2723475	5.6435747	0.21673897	72.7567437
TCGA-BP-4166-01A-02R-1289-07	1	27.992697	7.8770848	7.1545605	0.13490453	47.0445874
TCGA-BP-4167-01A-02R-1325-07	1	35.395938	3.5736418	5.4194625	0.47237742	43.1912505
TCGA-BP-4169-01A-02R-1289-07	2	33.631529	5.9564661	8.158805	0.59287458	87.3228323

TCGA-BP-4170-01A-02R-1289-07	1	46.033135	6.2058391	7.3951517	0.09715427	58.6407536
TCGA-BP-4173-01A-02R-1289-07	2	37.618097	5.4397603	6.9105775	0.41996756	91.3350097
TCGA-BP-4174-01A-02R-1289-07	1	39.270759	7.523357	4.3516996	0.48994377	59.9389567
TCGA-BP-4176-01A-02R-1289-07	1	39.627571	6.9500527	4.3635309	0.53888967	67.6329033
TCGA-BP-4177-01A-02R-1420-07	1	58.945649	7.4980157	10.727258	5.29536005	56.3647837
TCGA-BP-4325-01A-02R-1289-07	2	54.725675	5.9218895	4.9057824	0.1704717	88.3939824
TCGA-BP-4326-01A-01R-1289-07	2	55.436931	3.8309407	5.9126257	0.34058025	79.5916675
TCGA-BP-4327-01A-01R-1289-07	1	61.999635	4.7666821	5.0014913	0.20508782	68.8110581
TCGA-BP-4329-01A-02R-1289-07	1	50.023212	6.2425267	5.9524321	0.209887	49.5386005
TCGA-BP-4330-01A-01R-1289-07	2	45.438244	7.4092942	6.7583533	0.22635994	76.5477309
TCGA-BP-4331-01A-01R-1289-07	2	47.395019	5.3437899	8.4576005	0.26439958	78.9112268
TCGA-BP-4332-01A-01R-1289-07	2	45.952891	5.9393926	7.4504557	0.17165536	91.6780294
TCGA-BP-4334-01A-01R-1289-07	1	21.565043	2.9970858	5.7315754	5.50048206	39.262084
TCGA-BP-4335-01A-01R-1289-07	2	32.939426	4.0369522	3.8717206	0.41631694	93.999991
TCGA-BP-4337-01A-01R-1289-07	2	40.673502	3.5874932	6.0925378	0.66310354	77.0130074
TCGA-BP-4338-01A-01R-1289-07	1	62.000625	7.1701332	3.6440217	0.79649852	51.105422
TCGA-BP-4340-01A-01R-1289-07	2	45.35698	3.2522704	4.720803	0.09676524	86.5557593
TCGA-BP-4341-01A-01R-1289-07	2	45.188369	4.2274686	4.8146414	0.28820275	76.5082974
TCGA-BP-4342-01A-01R-1289-07	1	34.034982	6.6462364	6.9960768	0.62463566	62.7192066
TCGA-BP-4343-01A-02R-1289-07	1	20.386826	7.86483	4.5341766	0.33470201	41.2263211
TCGA-BP-4344-01A-01R-1289-07	2	36.801062	6.3537174	9.0516309	0.18902953	81.1875598
TCGA-BP-4345-01A-01R-1289-07	2	42.359435	4.8452454	9.2272482	0.52021201	77.7027544
TCGA-BP-4346-01A-01R-1289-07	2	44.571586	5.0348011	6.2138492	0.45949754	94.1364971
TCGA-BP-4347-01A-01R-1289-07	2	45.688874	3.9935106	7.4656547	0.13085401	79.7031762
TCGA-BP-4349-01A-01R-1289-07	2	45.1396	4.5173577	6.6346731	0.11006336	85.3117613
TCGA-BP-4351-01A-01R-1289-07	2	36.481945	4.1941922	5.0616965	0.39571923	99.1777692
TCGA-BP-4352-01A-01R-1289-07	2	48.484403	5.952599	7.4428558	12.4523668	94.4734301
TCGA-BP-4353-01A-02R-1289-07	1	25.603935	7.5878019	5.6398825	1.61839417	58.3042445
TCGA-BP-4354-01A-02R-1289-07	2	36.102641	6.2476596	8.3062906	7.29775801	125.326887
TCGA-BP-4355-01A-01R-1289-07	2	39.014781	5.2578431	7.6690691	0.25273567	102.023218
TCGA-BP-4756-01A-01R-1289-07	2	55.716649	3.6215004	10.801618	4.01264328	80.049209
TCGA-BP-4758-01A-01R-1289-07	1	39.089222	3.8200966	6.2680347	0.24436372	51.6855374
TCGA-BP-4759-01A-01R-1289-07	1	34.151162	5.7561279	7.5634401	0.14660912	43.9372987
TCGA-BP-4760-01A-02R-1420-07	1	34.958802	7.7599004	11.287404	5.0635703	56.5422326
TCGA-BP-4761-01A-01R-1289-07	1	61.503669	4.9830176	4.5425295	0.47139269	25.5710737
TCGA-BP-4762-01A-02R-1289-07	1	33.378453	9.2478218	7.4614138	0.50997812	46.145675
TCGA-BP-4763-01A-01R-1289-07	1	30.641622	8.509137	5.2731559	0.39806272	46.6811123
TCGA-BP-4765-01A-01R-1289-07	2	52.717737	6.3000791	8.103166	0.19574005	91.6684718
TCGA-BP-4766-01A-01R-1289-07	2	60.304563	6.5740565	8.4207626	0.27946739	75.5779241
TCGA-BP-4768-01A-01R-1289-07	1	55.994792	8.7136446	5.9883943	0.07869266	57.1307346
TCGA-BP-4769-01A-01R-1289-07	1	79.833647	6.0197409	12.682667	3.89346739	59.3276927
TCGA-BP-4770-01A-01R-1503-07	2	48.218662	5.3376187	14.931926	12.9811351	75.3641539
TCGA-BP-4771-01A-01R-1289-07	2	40.275999	4.6999767	7.9757758	0.35967747	87.1790686
TCGA-BP-4774-01A-01R-1289-07	2	52.095014	4.0817116	9.4715498	0.14997185	100.562465
TCGA-BP-4775-01A-01R-1289-07	2	52.259045	5.7325114	6.2484035	0.21399489	79.3728412
TCGA-BP-4776-01A-01R-1289-07	1	39.527843	3.7304278	5.8197128	0.15019181	67.1455561
TCGA-BP-4777-01A-01R-1289-07	1	35.731118	5.180824	6.1038836	0.17519367	71.8073895
TCGA-BP-4781-01A-01R-1305-07	1	54.724747	7.8680821	8.6476419	1.57282358	55.2257658
TCGA-BP-4782-01A-02R-1420-07	1	44.866518	5.2151516	7.1887981	1.48333606	62.1902253
TCGA-BP-4784-01A-01R-1305-07	1	46.717936	7.5149481	12.212992	4.97926788	62.4384774
TCGA-BP-4787-01A-01R-1305-07	1	26.048555	6.6962408	5.2683817	3.09532482	53.1925898
TCGA-BP-4789-01A-01R-1305-07	1	45.131366	8.376158	9.3134413	0.11213754	57.0311005

TCGA-BP-4790-01A-01R-1305-07	1	47.542726	7.8304461	10.6368	0.19235858	46.6218936
TCGA-BP-4795-01A-02R-1420-07	1	41.506484	6.8872785	11.910581	3.72904263	62.1721096
TCGA-BP-4797-01A-01R-1305-07	1	37.626547	7.4254932	6.3016009	0.34989179	47.060208
TCGA-BP-4798-01A-01R-1305-07	1	33.828439	5.6473977	7.1279819	3.08017285	72.5567283
TCGA-BP-4799-01A-01R-1305-07	1	18.569483	5.3404026	4.2927234	3.16477153	35.1703839
TCGA-BP-4801-01A-02R-1420-07	1	43.735529	7.6741649	8.2726987	0.2145503	48.6176176
TCGA-BP-4803-01A-01R-1305-07	1	43.317436	9.2261029	7.7308037	0.17954822	33.7422074
TCGA-BP-4804-01A-02R-1305-07	1	33.756245	5.0463482	8.077967	0.99056839	57.2123216
TCGA-BP-4807-01A-01R-1305-07	1	44.047619	8.0243089	6.0488512	0.19646513	41.0566545
TCGA-BP-4959-01A-01R-1334-07	1	42.722905	5.4967523	5.7000062	0.85930408	47.1252726
TCGA-BP-4960-01A-01R-1334-07	1	28.184083	2.7138676	5.6045018	1.07734429	62.3617735
TCGA-BP-4961-01A-01R-1334-07	2	38.671722	8.5403318	9.8523903	0.28299502	72.8028225
TCGA-BP-4962-01A-01R-1334-07	1	37.35086	4.449897	5.2511594	0.14119	43.4419327
TCGA-BP-4963-01A-01R-1334-07	2	55.690693	5.8030823	7.7428889	0.77808612	87.3113085
TCGA-BP-4964-01A-01R-1334-07	1	45.173814	7.3419088	5.8790685	0.25834764	61.9397392
TCGA-BP-4965-01A-01R-1334-07	2	45.727473	8.0416071	6.2056958	0.05424895	73.5977917
TCGA-BP-4967-01A-01R-1334-07	2	40.779257	4.0316888	8.6709149	0.17935376	77.9019544
TCGA-BP-4968-01A-01R-1334-07	2	52.114742	3.4963216	5.840114	0.19068025	90.0686377
TCGA-BP-4969-01A-01R-1334-07	2	32.516476	3.1674655	3.4827203	0.16440195	97.0304582
TCGA-BP-4970-01A-01R-1334-07	1	41.346528	7.0604585	6.0869397	1.87301363	65.8711022
TCGA-BP-4971-01A-01R-1334-07	2	25.408846	3.7960898	4.7513312	0.33333893	100.900663
TCGA-BP-4972-01A-01R-1334-07	1	49.698804	7.6114111	6.5118648	0.10436627	53.2799205
TCGA-BP-4973-01A-01R-1334-07	2	40.861194	6.5088811	4.9153438	0.3535353	86.7695987
TCGA-BP-4974-01A-01R-1334-07	2	36.387551	5.6726769	9.1535324	0.22811869	108.342459
TCGA-BP-4975-01A-01R-1334-07	1	50.214207	8.5124857	6.0238205	0.28140065	49.171367
TCGA-BP-4976-01A-01R-1334-07	1	53.753124	9.262578	8.3580971	0.13048745	58.1487192
TCGA-BP-4977-01A-01R-1334-07	2	45.126053	6.1874662	5.6295306	0.92905277	79.8403457
TCGA-BP-4981-01A-01R-1334-07	1	38.47075	3.1198577	4.1898584	0.28359022	67.4759446
TCGA-BP-4982-01A-01R-1334-07	2	45.078159	7.1707465	7.2749299	0.44191806	76.505864
TCGA-BP-4983-01A-01R-1334-07	2	48.731286	11.640945	6.8260842	20.4728668	112.969525
TCGA-BP-4985-01A-01R-1334-07	1	55.416154	6.7212638	5.0905235	17.033842	59.0422342
TCGA-BP-4986-01A-01R-1334-07	2	41.103733	8.4748936	7.3562411	0.26412494	81.1168485
TCGA-BP-4987-01A-01R-1334-07	2	59.443929	6.9937186	7.6310715	0.11616907	79.5900311
TCGA-BP-4989-01A-01R-1334-07	2	27.699192	3.8509348	5.6434003	0.30216881	131.675878
TCGA-BP-4991-01A-01R-1334-07	2	39.795766	7.8515093	8.9435286	0.25738238	87.7941676
TCGA-BP-4992-01A-01R-1334-07	2	25.231113	2.0777698	3.2530306	0.38787138	138.687438
TCGA-BP-4993-01A-02R-1420-07	1	49.223905	5.4972143	7.9169796	0.78993729	67.9593293
TCGA-BP-4994-01A-01R-1334-07	1	35.489973	4.858865	10.787061	4.13667966	71.4146306
TCGA-BP-4995-01A-01R-1334-07	1	43.737861	5.9378265	9.5141917	2.82581191	67.8862954
TCGA-BP-4998-01A-01R-1334-07	2	42.486226	6.180323	5.8526115	0.80913764	95.6770483
TCGA-BP-4999-01A-01R-1334-07	2	38.819832	7.1443453	8.337803	0.11369892	82.2325406
TCGA-BP-5000-01A-01R-1334-07	1	35.306548	3.8047344	5.1603383	0.20335394	51.7031983
TCGA-BP-5001-01A-01R-1334-07	2	37.63349	3.0567473	4.4830456	0.21082038	98.1659837
TCGA-BP-5004-01A-01R-1334-07	1	41.970821	5.1533696	7.419531	0.23757196	69.9537233
TCGA-BP-5006-01A-01R-1334-07	2	50.958472	4.7869368	8.132162	0.1854807	71.0911009
TCGA-BP-5007-01A-01R-1334-07	2	44.023244	6.1876948	5.5574744	0.11778691	81.2711408
TCGA-BP-5008-01A-01R-1334-07	2	42.171102	6.3359763	8.2933109	0.40118496	76.863169
TCGA-BP-5009-01A-01R-1334-07	2	38.922613	3.9818203	6.1992146	0.27439162	77.8197151
TCGA-BP-5010-01A-02R-1420-07	1	33.071136	3.2680676	7.6966445	0.54106006	61.5743629
TCGA-BP-5168-01A-01R-1420-07	1	43.274471	5.5875311	7.6750128	0.05405102	57.6879246
TCGA-BP-5169-01A-01R-1426-07	1	37.103607	4.5214114	5.6771729	10.7027466	58.6917974
TCGA-BP-5170-01A-01R-1426-07	1	41.391089	8.0261835	6.8674071	0.22752222	45.4524787

TCGA-BP-5173-01A-01R-1426-07	1	48.486477	6.0215353	7.5364308	0.09682537	63.615669
TCGA-BP-5174-01A-01R-1426-07	1	43.27881	3.5963743	5.3721297	0.58267544	70.9920204
TCGA-BP-5175-01A-01R-1426-07	1	31.901777	2.7736503	4.6236615	0.18557046	48.7201227
TCGA-BP-5176-01A-01R-1426-07	1	52.919932	4.2980568	5.8159046	0.07944214	59.7619429
TCGA-BP-5177-01A-01R-1426-07	2	40.540975	5.0245135	8.1892555	0.34805188	75.6601994
TCGA-BP-5178-01A-01R-1426-07	1	35.322318	3.5071091	5.9866642	3.10798501	54.8594161
TCGA-BP-5180-01A-01R-1426-07	1	48.5175	6.2297297	5.9351043	0.28233021	37.8492438
TCGA-BP-5181-01A-01R-1426-07	1	58.00915	6.3283067	4.795727	0.23922982	54.366075
TCGA-BP-5182-01A-01R-1426-07	1	42.648062	6.0521455	7.4985688	0.3236	53.7416298
TCGA-BP-5183-01A-01R-1426-07	1	46.05578	5.3865741	6.1838156	0.25943207	47.8843777
TCGA-BP-5184-01A-01R-1426-07	1	45.883446	6.7002208	8.0112041	0.41181285	61.451653
TCGA-BP-5185-01A-01R-1426-07	1	27.297961	2.4288076	3.458781	0.12860708	30.7957692
TCGA-BP-5186-01A-01R-1426-07	1	61.303102	6.7392739	7.1590435	0.35533685	68.5179519
TCGA-BP-5187-01A-01R-1426-07	1	33.293684	8.0920237	5.4111251	0.45026224	38.7103236
TCGA-BP-5189-01A-02R-1426-07	1	34.302148	8.0608455	6.6029492	0.53223349	64.0468148
TCGA-BP-5190-01A-01R-1426-07	1	36.118107	3.9592479	4.3490049	0.11723424	60.0496434
TCGA-BP-5191-01A-01R-1426-07	1	34.427929	3.4938355	3.1406488	0.67735617	40.9434155
TCGA-BP-5192-01A-01R-1426-07	1	71.278337	7.1922391	7.7013257	0.05794074	64.2144453
TCGA-BP-5194-01A-02R-1426-07	1	42.585734	8.4907068	7.572342	0.31304044	65.7735361
TCGA-BP-5195-01A-02R-1426-07	1	42.360448	6.4101968	9.5034997	0.30340938	57.4569852
TCGA-BP-5196-01A-01R-1426-07	2	49.943282	7.185758	8.3576	0.82092703	77.1822637
TCGA-BP-5198-01A-01R-1426-07	1	33.836163	5.4604701	8.4914473	1.99287042	67.7970653
TCGA-BP-5199-01A-01R-1426-07	2	45.421257	5.4050368	8.5662911	0.26572864	87.7075031
TCGA-BP-5200-01A-01R-1426-07	1	50.358067	4.7860046	8.6457361	0.18595847	69.6046795
TCGA-BP-5201-01A-01R-1426-07	1	48.608993	3.8460005	7.4888979	0.19187185	70.925675
TCGA-BP-5202-01A-02R-1426-07	1	58.591762	6.1683642	9.4352503	0.31877531	60.4891601
TCGA-CJ-4634-01A-02R-1325-07	2	49.372493	7.2859645	7.789961	0.56703874	78.7764989
TCGA-CJ-4635-01A-02R-1305-07	2	42.804481	7.7893833	7.7822154	0.63091469	80.9399162
TCGA-CJ-4636-01A-02R-1325-07	2	30.887114	4.7582639	4.9137626	0.83438224	85.1801241
TCGA-CJ-4637-01A-02R-1325-07	2	48.161625	6.1352473	5.9270932	1.26090718	89.1554154
TCGA-CJ-4638-01A-02R-1325-07	1	46.619038	3.0496917	3.61737	0.45394345	69.1519848
TCGA-CJ-4639-01A-02R-1325-07	1	41.589868	7.9919994	5.6654282	0.20287551	64.404085
TCGA-CJ-4640-01A-02R-1325-07	1	43.465885	4.2530775	7.5864072	0.56178084	59.4888852
TCGA-CJ-4641-01A-02R-1325-07	2	35.954027	5.8453365	5.8719677	1.59119897	75.0943826
TCGA-CJ-4642-01B-01R-1305-07	1	32.13002	6.3796724	7.9781088	4.34878541	58.4165571
TCGA-CJ-4643-01A-02R-1325-07	2	48.735584	6.4468904	7.0184287	0.16989277	82.195844
TCGA-CJ-4644-01A-02R-1325-07	1	43.098378	6.5604483	5.778789	0.06567405	53.1517924
TCGA-CJ-4868-01A-01R-1305-07	1	51.507665	5.7540543	7.4590938	8.1587594	65.7465301
TCGA-CJ-4869-01A-02R-1426-07	1	44.571296	4.441261	5.7721868	0.30092244	56.9233124
TCGA-CJ-4870-01A-01R-1305-07	1	40.585445	4.9308053	6.3883646	0.08559176	43.0409207
TCGA-CJ-4871-01A-01R-1305-07	1	36.218988	6.634693	5.4872805	0.35981762	51.3446338
TCGA-CJ-4872-01A-01R-1305-07	1	36.068093	5.3440503	5.546706	0.32937853	56.6657383
TCGA-CJ-4873-01A-01R-1305-07	1	38.791063	3.2452091	4.6911204	0.47543865	75.3928436
TCGA-CJ-4874-01A-01R-1305-07	1	40.60613	6.151564	4.8849213	0.16271333	54.209817
TCGA-CJ-4875-01A-01R-1305-07	2	25.524412	4.0558	4.275305	0.10849187	76.0504985
TCGA-CJ-4876-01A-01R-1305-07	2	66.366362	2.8590654	7.7146775	0.09307613	89.8871177
TCGA-CJ-4878-01A-01R-1305-07	2	48.728316	4.7063512	5.2214215	0.19258644	125.935824
TCGA-CJ-4881-01A-01R-1305-07	2	33.887703	5.0055589	5.641707	0.2489471	91.2200889
TCGA-CJ-4882-01A-02R-1426-07	1	52.375186	3.3085089	7.2240927	0.66557096	61.619597
TCGA-CJ-4884-01A-01R-1305-07	2	34.118452	4.7485458	5.1445561	0.4018713	89.3515527
TCGA-CJ-4885-01A-01R-1305-07	1	48.356409	3.8409736	4.9869384	0.12242533	67.1852208
TCGA-CJ-4886-01A-01R-1305-07	1	41.797193	6.6771773	6.9617483	0.1754685	69.6202687

TCGA-CJ-4887-01A-01R-1305-07	2	34.56068	3.6221352	5.6807563	0.30558062	81.0809386
TCGA-CJ-4888-01A-01R-1305-07	2	43.463907	3.567146	7.640063	0.43699882	76.5233939
TCGA-CJ-4889-01A-01R-1305-07	1	43.811501	5.3744066	11.222929	0.37379504	68.366257
TCGA-CJ-4890-01A-01R-1305-07	1	30.047824	5.4480153	8.5714092	0.80587314	69.5259326
TCGA-CJ-4891-01A-01R-1305-07	1	50.797334	2.8930472	3.7998031	2.93476802	59.6813029
TCGA-CJ-4892-01A-01R-1305-07	1	51.039743	5.0494307	5.5415097	0.3717572	67.2465078
TCGA-CJ-4893-01A-01R-1305-07	1	45.530482	6.1235059	6.2904109	0.10523877	60.6040896
TCGA-CJ-4894-01A-01R-1305-07	2	40.121673	5.1247561	7.7368988	0.12405178	86.1508358
TCGA-CJ-4895-01A-01R-1305-07	1	62.005259	5.5877987	7.2285776	1.63710029	67.7189944
TCGA-CJ-4897-01A-03R-1426-07	1	35.496023	7.2094971	5.9731337	0.11838564	46.566882
TCGA-CJ-4899-01A-01R-1334-07	2	47.782445	6.6837664	8.6616622	0.53767578	92.5344178
TCGA-CJ-4900-01A-01R-1334-07	2	46.947366	4.1524617	6.6855821	3.53733997	96.1472645
TCGA-CJ-4901-01A-01R-1426-07	1	36.861748	5.5286333	4.5061495	2.97059078	62.5799025
TCGA-CJ-4902-01A-01R-1426-07	2	34.813844	5.9327277	7.5831992	0.50453009	73.6333114
TCGA-CJ-4903-01A-01R-1426-07	1	41.41436	5.1280218	5.7881256	0.28155069	63.6793221
TCGA-CJ-4904-01A-02R-1426-07	1	47.201679	7.0409705	7.9203807	0.16284605	66.298082
TCGA-CJ-4905-01A-02R-1426-07	1	48.178736	8.2759782	9.4393095	0.15069637	65.2193485
TCGA-CJ-4907-01A-01R-1426-07	1	44.709743	7.0503518	8.53936	0.31283615	56.7809053
TCGA-CJ-4908-01A-01R-1426-07	2	44.59521	7.2891789	8.8889617	0.4558292	84.0817354
TCGA-CJ-4912-01A-01R-1426-07	1	26.864066	6.6760142	6.4709156	0.81945471	72.2342536
TCGA-CJ-4916-01A-01R-1426-07	2	47.407644	4.5454167	5.805622	0.13677679	81.6612337
TCGA-CJ-4918-01A-01R-1426-07	1	33.352764	6.9899671	7.7707942	0.75435608	57.3026592
TCGA-CJ-4920-01A-01R-1426-07	1	48.691973	5.3011576	7.4526139	3.83310799	66.3271389
TCGA-CJ-5671-01A-11R-1541-07	1	31.987844	5.2398426	7.9851579	3.48215356	52.5254693
TCGA-CJ-5672-01A-11R-1541-07	1	39.618869	4.6629447	6.1944722	0.31639735	52.675841
TCGA-CJ-5675-01A-11R-1541-07	1	50.985762	5.1195386	6.6744069	0.20588409	62.1040252
TCGA-CJ-5676-01A-11R-1541-07	1	32.884469	3.9173554	7.6894725	1.50423499	55.6764005
TCGA-CJ-5677-01A-11R-1541-07	1	27.63148	5.8945802	6.9283137	0.37222253	60.9346235
TCGA-CJ-5678-01A-11R-1541-07	2	43.42505	4.3246304	5.4262946	0.19037479	71.9331657
TCGA-CJ-5679-01A-11R-1541-07	1	48.45556	3.2971298	5.9790806	3.11067663	56.2683321
TCGA-CJ-5680-01A-11R-1541-07	1	61.941601	6.2180873	6.8381885	3.71030721	61.629486
TCGA-CJ-5681-01A-11R-1541-07	2	68.435063	5.7546374	7.0991207	5.3709856	76.7963183
TCGA-CJ-5682-01A-11R-1541-07	1	42.633528	4.918733	7.6597506	0.41337337	55.8456866
TCGA-CJ-5683-01A-11R-1541-07	1	50.525313	5.4123124	7.7175936	1.59830874	58.5206
TCGA-CJ-5684-01A-11R-1541-07	1	47.488207	5.9884846	8.2899267	0.24397205	56.6072257
TCGA-CJ-5686-01A-11R-1672-07	1	46.977142	6.1289627	6.1553458	0.40567905	57.4654985
TCGA-CJ-5689-01A-11R-1541-07	1	48.687167	4.3471832	8.2724486	0.84251224	60.583324
TCGA-CJ-6027-01A-11R-1672-07	1	47.503082	5.5412656	5.3995695	0.72985489	69.302755
TCGA-CJ-6028-01A-11R-1672-07	1	44.425115	8.5417114	5.9880348	0.94334875	54.8124119
TCGA-CJ-6030-01A-11R-1672-07	1	39.953929	5.0457152	5.8906975	1.20557327	71.0215428
TCGA-CJ-6031-01A-11R-1672-07	1	34.256382	5.9605529	7.3748415	0.29660952	51.6027258
TCGA-CJ-6032-01A-11R-1672-07	1	46.64541	6.2893545	8.0088563	0.10265982	60.2541595
TCGA-CJ-6033-01A-11R-1672-07	1	47.541024	5.1601666	6.7859918	0.34820438	60.2059492
TCGA-CW-5580-01A-01R-1672-07	1	42.990533	8.5746168	7.3841651	3.03397154	49.4982423
TCGA-CW-5581-01A-02R-1541-07	1	46.761174	7.5158232	6.3519575	0.1460986	47.2337742
TCGA-CW-5583-01A-02R-1541-07	1	49.107212	9.282507	6.6864698	0.15889839	58.7584317
TCGA-CW-5584-01A-01R-1541-07	1	43.293632	7.2733989	7.8516502	0.17511769	46.4418369
TCGA-CW-5585-01A-01R-1541-07	1	61.835775	7.488104	7.4453959	0.06399257	50.3047652
TCGA-CW-5587-01A-01R-1541-07	1	38.28519	7.0941219	5.9860932	0.26607224	54.0813915
TCGA-CW-5588-01A-01R-1541-07	1	34.892246	10.441887	7.7764323	0.67748184	36.6841646
TCGA-CW-5589-01A-01R-1541-07	1	48.067952	7.6543985	7.6896368	0.41328645	52.387994
TCGA-CW-5590-01A-01R-1541-07	1	50.761708	4.8976477	8.865829	0.21180579	64.9374272

TCGA-CW-5591-01A-01R-1541-07	1	28.284755	6.5478598	10.916867	5.21131297	66.6310691
TCGA-CW-6087-01A-11R-1672-07	2	31.167782	7.9193487	10.278083	14.5354263	86.036821
TCGA-CW-6088-01A-11R-1672-07	2	52.434551	6.0790855	7.4725006	0.22244196	70.786584
TCGA-CW-6090-01A-11R-1672-07	1	47.438551	7.8938526	6.5684202	0.96572538	55.4120836
TCGA-CW-6093-01A-11R-1672-07	2	61.191562	8.0993494	10.706428	0.26696811	92.32453
TCGA-CW-6097-01A-11R-1672-07	1	39.28879	8.4575286	8.1571645	1.91135335	64.4436001
TCGA-CZ-4853-01A-01R-1426-07	1	41.020662	7.4526645	4.4214322	0.14564029	51.0903625
TCGA-CZ-4854-01A-01R-1305-07	2	38.304033	5.9781483	5.8484436	1.35064877	88.5656708
TCGA-CZ-4856-01A-02R-1426-07	1	45.065759	6.7509418	5.9754221	0.13415489	47.115981
TCGA-CZ-4857-01A-01R-1305-07	1	36.062873	8.0920215	6.4620269	10.0362204	70.4068581
TCGA-CZ-4858-01A-01R-1305-07	2	40.097954	7.5368466	8.9019809	4.20322988	88.3381029
TCGA-CZ-4859-01A-02R-1426-07	1	69.281558	6.6018549	5.8562773	0.04932331	53.728319
TCGA-CZ-4860-01A-01R-1305-07	1	30.848697	11.956571	7.442825	4.80602147	47.2608628
TCGA-CZ-4861-01A-01R-1305-07	1	32.231438	8.9447836	5.1253477	7.12820197	54.7653535
TCGA-CZ-4862-01A-01R-1305-07	2	41.771911	5.0742329	9.269045	2.02453727	92.7952843
TCGA-CZ-4863-01A-01R-1503-07	2	42.461008	6.355898	6.4420427	0.21084168	77.9432341
TCGA-CZ-4864-01A-01R-1503-07	2	33.116657	6.1427239	6.418015	0.22035462	92.7311673
TCGA-CZ-4865-01A-02R-1503-07	2	50.829081	5.6196368	7.9940751	0.2615231	76.2975618
TCGA-CZ-4866-01A-01R-1503-07	2	36.613581	6.4069983	4.9192638	0.17539149	89.7095807
TCGA-CZ-5451-01A-01R-1503-07	1	36.346976	6.2378373	5.2426422	0.13656154	54.1224999
TCGA-CZ-5452-01A-01R-1503-07	2	50.674618	4.6063807	6.0248137	0.20249816	74.8216566
TCGA-CZ-5453-01A-01R-1503-07	2	54.233898	3.6075911	6.3629226	0.07031557	77.8955966
TCGA-CZ-5454-01A-01R-1503-07	1	38.76002	7.1796178	4.5988992	0.28535973	65.8168777
TCGA-CZ-5455-01A-01R-1503-07	2	41.188119	7.9976688	5.8516249	0.20574914	78.8446893
TCGA-CZ-5456-01A-01R-1503-07	1	52.116313	8.00331	6.8372892	6.69280003	49.0415645
TCGA-CZ-5457-01A-01R-1503-07	1	38.573554	5.2712111	6.678934	0.18581841	59.5903118
TCGA-CZ-5458-01A-01R-1503-07	1	44.653591	6.8438281	6.5640564	0.52486852	59.0092469
TCGA-CZ-5459-01A-01R-1503-07	1	39.313782	5.184773	6.1800031	0.27960214	58.0446572
TCGA-CZ-5460-01A-01R-1503-07	1	45.152616	4.0327829	6.4784547	0.4181175	67.8832715
TCGA-CZ-5461-01A-01R-1503-07	1	38.521431	7.3765368	6.72139	0.14035206	52.2022942
TCGA-CZ-5462-01A-01R-1503-07	1	54.795832	8.351463	5.1928728	0.66815533	60.4475866
TCGA-CZ-5463-01A-01R-1503-07	2	39.577003	6.2979398	4.9165043	0.07889176	71.7511753
TCGA-CZ-5464-01A-01R-1503-07	2	37.19323	7.2906224	5.9437525	0.33857396	75.3589953
TCGA-CZ-5465-01A-01R-1503-07	2	57.792673	8.9088637	6.8904392	0.12052198	69.2055653
TCGA-CZ-5466-01A-01R-1503-07	1	38.228199	9.5759369	7.6029244	1.29489894	46.2412015
TCGA-CZ-5467-01A-01R-1503-07	1	48.151276	7.5324345	7.84511	0.10887935	60.3471437
TCGA-CZ-5468-01A-01R-1503-07	1	47.718843	5.9754686	5.8876853	9.68208253	70.9564026
TCGA-CZ-5469-01A-01R-1503-07	1	46.553285	5.181802	6.8809423	13.3512437	61.1376939
TCGA-CZ-5470-01A-01R-1503-07	2	42.025404	5.8071134	5.2822828	1.08383776	86.7469715
TCGA-CZ-5982-01A-11R-1672-07	2	57.518754	7.4930561	8.2857616	0.27396721	90.5261208
TCGA-CZ-5984-01A-11R-1672-07	1	50.088666	6.1131352	3.7889687	0.17345031	57.275178
TCGA-CZ-5985-01A-11R-1672-07	1	52.156744	7.3538436	4.8542044	0.36683389	54.0058186
TCGA-CZ-5986-01A-11R-1672-07	2	53.038059	5.6570702	6.7090557	0.10741645	69.7468136
TCGA-CZ-5987-01A-11R-1672-07	1	51.137014	4.1177721	5.9253992	0.18318683	56.7792645
TCGA-CZ-5988-01A-11R-1672-07	1	45.69891	5.2592459	6.8830149	0.69030605	63.6862202
TCGA-CZ-5989-01A-11R-1672-07	2	13.620815	10.134819	2.6395641	0.12799742	113.905482
TCGA-DV-5565-01A-01R-1541-07	2	31.86553	4.5642449	10.047331	0.23683573	83.0014018
TCGA-DV-5566-01A-01R-1541-07	2	59.240117	4.7558198	9.3992244	0.5433656	73.8239103
TCGA-DV-5567-01A-01R-1541-07	1	39.028383	8.4535396	14.748198	0.30469197	54.6130041
TCGA-DV-5568-01A-01R-1541-07	2	46.997538	4.5205006	7.41203	0.3812577	71.8791627
TCGA-DV-5569-01A-01R-1541-07	2	44.988692	6.6840391	7.850238	0.25869897	72.9170183
TCGA-DV-5573-01A-01R-1541-07	2	44.227744	5.916008	7.6620012	1.55553013	86.7191937

TCGA-DV-5574-01A-01R-1541-07	2	40.520239	5.7691842	7.6420571	0.78877471	74.0483015
TCGA-DV-5575-01A-01R-1541-07	1	57.457689	4.6884078	6.9118047	0.3562835	68.0523024
TCGA-DV-5576-01A-01R-1541-07	1	46.347081	7.1019585	10.017656	1.66126197	70.3506488
TCGA-DV-A4VX-01A-11R-A266-07	2	20.368589	4.0224035	3.8755195	3.20584372	78.663191
TCGA-DV-A4VZ-01A-11R-A266-07	2	37.838759	3.4311103	10.907323	12.40416	119.630689
TCGA-DV-A4W0-01A-11R-A266-07	2	54.245719	4.9382895	6.4190265	0.42635891	91.390935
TCGA-DV-A4W0-05A-11R-A266-07	2	38.929807	6.5494936	10.934251	6.33461229	99.8562693
TCGA-EU-5904-01A-11R-1672-07	1	39.343463	6.6652272	5.9970931	0.17353057	64.0342884
TCGA-EU-5905-01A-11R-1672-07	1	28.15349	6.9435287	6.3379076	0.43890094	42.5170286
TCGA-EU-5906-01A-11R-1672-07	1	47.95905	8.1187756	7.4905474	0.14931285	55.3592289
TCGA-EU-5907-01A-11R-1672-07	1	58.293447	7.0730369	5.6061723	0.17149638	51.77907
TCGA-G6-A5PC-01A-11R-A33J-07	1	43.277544	2.9102459	3.3673067	0.15467565	46.8867652
TCGA-G6-A8L6-01A-11R-A37O-07	1	44.87267	4.0729638	7.1373279	0.26661942	68.2388228
TCGA-G6-A8L7-01A-11R-A37O-07	1	31.229159	2.7241334	5.3812775	0.2745868	69.282177
TCGA-G6-A8L8-01A-21R-A37O-07	2	51.323763	4.2293818	6.3148385	0.17180069	92.569908
TCGA-GK-A6C7-01A-11R-A33J-07	2	41.839754	5.4191266	10.745004	0.16327451	75.6168473
TCGA-MM-A563-01A-11R-A266-07	2	48.997948	3.851311	7.7754908	0.62359986	81.842168
TCGA-MM-A564-01A-11R-A266-07	2	47.208153	5.0194379	6.0070916	0.10762056	72.3245938
TCGA-MM-A84U-01A-11R-A37O-07	1	45.940519	3.3994126	5.0732217	3.6574319	60.9826317
TCGA-MW-A4EC-01A-11R-A266-07	2	56.207554	4.9521417	7.4604375	0.0858671	71.9907265
TCGA-T7-A92I-01A-11R-A37O-07	2	35.929667	4.3256089	11.222906	0.08569596	89.0281879

IGF2BP1	YTHDC1	YTHDF2	YTHDF1	METTL14	ZC3H13	HNRNPC	YTHDF3	IGF2BP3
0.1952889	14.7776784	18.3082281	22.8490755	3.858316	4.7223248	43.43576	9.414466	0.02090103
0.2329979	10.4550799	16.6230303	17.1790451	3.0581913	5.3246167	45.30886	9.545742	0.00840569
0.0052118	10.1673113	21.6765986	15.8415979	4.6734182	5.4296165	47.2731	16.9161	0.01952304
1.1365986	11.8102715	24.8529596	22.0632294	4.8750349	5.9513924	54.11193	21.9658	0.02101872
0.0290449	12.5344813	22.9356828	21.6462341	3.395876	7.7619324	45.12476	27.28617	0.42085174
1.3840795	11.9582775	21.4792573	15.9737773	3.9001651	8.1670871	37.91165	19.44502	1.1246791
0.2918414	11.5380746	11.8780633	20.6668727	3.9772202	8.7362078	42.45119	23.41773	0.01848206
2.0055476	9.1418285	14.3739065	14.6937896	3.5604408	7.8537975	32.84839	20.78995	0.02597835
0.1654856	12.548728	17.1522312	19.0562403	4.7439873	9.9956527	37.10003	20.4109	1.61988677
2.2038598	11.1156644	14.5001059	19.1529726	2.8211835	3.9639838	29.19426	18.96532	2.78886203
0.3596506	21.7731809	16.6005059	15.1746223	6.003621	9.8522574	46.26213	19.49728	0.01994406
0.097933	14.0813403	14.5800081	13.6923088	4.1848179	8.2021041	41.67757	12.97893	0.017469
1.5060591	14.1641155	13.5935905	11.1286304	4.3570444	8.574514	35.54305	13.45126	0.06908048
0.0270543	13.6960675	17.3540594	15.2063562	4.4599858	7.9704976	34.67789	18.4713	0.02895514
0.8109396	13.6625722	17.8497976	18.7797103	3.9342211	7.8955272	53.75401	14.80434	0.01491734
0.594414	18.1968523	13.066258	13.1605692	3.6925456	7.940951	49.80153	12.15559	0.01988058
0.0967456	9.54336288	11.2520998	20.7222375	3.8722214	7.4182631	42.72607	18.33755	0.16786533
1.3086513	13.2674032	18.8742013	16.6833243	4.1267766	5.1180247	50.95996	17.06943	0.0232377
0.0841758	14.3595095	18.0161352	15.8084669	4.4502468	8.7825614	39.72102	18.57822	0.03118506
0.0612103	9.72618638	11.1453442	10.9273876	2.8732279	3.4370603	39.09162	11.07248	0.03457526
0.5834259	14.1433012	15.6661638	13.4445355	3.7929883	5.9377359	53.81496	13.42207	0.03684457
0.4698912	4.73968424	14.8704502	16.3237085	1.5086282	2.2238619	46.93069	8.982813	1.32354136
1.6121888	10.7350586	13.53851	19.515714	3.7586549	6.8821879	36.25121	14.95349	2.59391005
0.1772686	16.1873471	16.8987148	13.107314	4.1336267	7.5222297	45.29226	13.20444	0.01600081
0.2932664	19.1258347	15.6340278	12.954366	4.6266578	9.3946631	40.8533	15.57092	0.04345918
1.0038999	15.7832607	17.7594109	14.9972106	4.519859	7.6250667	59.03598	15.92928	0.01578119
0.018996	15.8126397	18.5220168	14.4837017	3.6253284	6.606539	52.43093	16.34905	0.09226993
1.0171816	9.69580417	15.0440579	17.695366	2.9898688	3.7175502	44.85612	14.32963	1.60653754
0.5852488	15.4734612	21.7101679	17.7712855	4.7624095	7.1947672	50.32419	18.00337	0.02036974
0.4354099	12.2326381	18.9715475	15.3371691	4.4122853	6.977991	47.66031	14.43925	0.02291816
0.0588859	11.2405096	16.7067352	27.2899093	3.3872976	3.8900512	71.32685	15.10768	0.01750648
0.0170237	15.5134826	21.598707	22.4370414	5.5563261	6.2390025	68.84633	15.9019	0.03643959
1.6285999	16.3979956	14.920346	14.4948393	2.9397982	8.0123071	50.06125	15.0375	0.01826755
0.0120962	16.6474388	16.6429787	15.3951796	4.9259868	7.4112224	41.23058	16.49975	0.03452297
0.0086711	16.5472451	19.4033363	21.0562001	4.9126582	11.67265	45.08902	12.73136	0.03712122
0.027565	10.8905609	14.5325039	20.2408441	5.7283775	10.120648	40.9975	16.14382	0.02802672
0.0242668	4.99357445	9.17310654	16.2769126	2.6990001	1.2674297	31.97555	13.22969	0.07235018
0.0416585	17.3395678	20.9449547	16.9998633	4.5638634	11.850137	41.94962	18.66171	0.06163295
0.0313917	11.078593	17.3596846	15.5298209	3.1392217	7.9979108	34.46265	15.20029	0.09705899
0.5194252	16.1612537	14.3029562	16.2091467	5.3077046	6.5078539	42.1024	13.75474	0.02860044
0.9555423	12.2354198	14.9132653	17.060609	3.8001105	9.4021344	41.99818	15.25985	0.06509877
0.3920293	11.521382	17.1811925	16.2613277	3.5234821	2.6079967	64.49501	7.060124	0.01068978
0.6384779	15.1647746	14.6409074	16.2158596	4.6889695	7.830152	39.076	15.79362	0.01638702
1.9476254	11.5607448	16.5270439	15.0483418	3.6477844	7.1295687	40.14583	15.14782	1.53094653
0.2838322	13.8968435	17.9875128	15.6298092	3.3242011	5.4135725	38.36201	9.539822	0.01730363
0.6196248	13.3359633	24.2723753	21.7610121	4.5563519	7.75933	51.00722	14.47227	0.02399595
0.5213176	14.5261751	17.3448584	13.4950509	4.0802571	7.774236	41.14226	12.46833	0.00724606
0.5495128	15.7169677	16.428373	20.7548667	4.8992775	10.052252	47.83437	8.869089	0.04491119
1.3634063	16.876138	22.1052244	20.7938498	4.8293435	8.7725421	43.3833	15.73772	0.01228284
0.3071095	12.8027697	16.6339395	17.6191189	3.0658201	5.8113931	40.23445	10.49518	0.01998776

0.2048235	14.3010947	15.747534	17.7387009	4.562443	5.3188941	54.72505	10.43926	0.00481791
0.0503251	16.4956033	16.5068743	16.5834333	4.6735164	8.8999018	44.53812	12.11793	0.00897683
0.02948	10.7531168	19.0994012	16.8297103	3.6647314	7.6326003	40.76426	14.39411	0.02412751
1.7279978	7.77982702	17.2881036	11.1139296	3.1731457	5.0916682	44.19757	14.99332	0.03319455
0.1899073	6.56535148	12.9042324	11.8725761	1.3720496	3.4781573	31.40808	7.886805	0.06663952
0.0378956	3.70373591	9.13293977	12.9825747	2.0121896	2.2194461	38.42489	18.21733	0.01419538
0.003505	10.8467514	24.8121321	16.1425898	4.573674	7.8659698	81.5896	21.0483	0.0112539
0.0726988	9.31647197	20.3851656	15.0249865	3.9001085	4.4001464	54.17713	13.56091	0.02146393
0.066425	8.87980016	21.1670993	14.1285808	3.9114986	6.5475568	59.71348	19.96922	0.08246692
0.0312791	9.54532464	10.9457349	19.3774143	4.8822505	6.203597	29.18797	24.62569	0.10322025
0.5256554	11.087223	15.0398351	12.9741719	3.1646977	5.8071705	53.87117	14.96563	0.01136543
0.5958491	5.02338025	13.3778724	7.78958787	1.9130272	4.0349905	34.29236	11.56286	0.81780016
0.0069419	10.3546554	15.3947477	20.8082154	4.3633479	3.4004054	33.14831	13.14083	0.16159549
0.0014526	10.3153395	20.0210593	14.6910153	4.9942745	10.036419	42.11445	23.29705	0.21143733
0.0964219	9.44493041	15.8181776	21.9320219	3.2852254	4.3413183	40.37355	29.42213	0.01965652
0.0023825	2.28992124	6.73717056	3.40549101	1.4073587	1.5496746	25.53204	15.27189	0
1.1157334	10.1720723	25.7841077	15.6556065	3.634694	5.6093337	46.37353	17.06515	0.00620863
0.0148167	4.85583043	19.1133689	7.43446685	1.707881	2.3841883	46.42257	14.77836	0
0.3135325	12.1281444	13.6446895	15.3743951	2.6742079	4.1509721	37.80812	7.616895	0.03085627
0.1597851	6.6033273	13.0304702	13.2276538	1.6352817	4.1302296	27.57066	7.831525	0.20411106
0.8136981	11.5082276	21.1747875	15.4728839	4.3411947	4.283191	57.24491	21.38567	0.10678245
0.3238109	8.71862959	22.2368738	14.5328773	3.6383271	7.7973035	46.52716	22.07358	0.04950893
0.4788044	7.47769901	11.8293924	8.47629374	1.9263313	3.1855368	57.4045	15.73226	0.02890722
0.0660461	12.0035647	18.4638584	14.2949008	3.8004461	6.7475974	49.22938	14.08657	0.04302665
0.1553514	12.9142795	21.1056302	19.9305439	4.2586494	7.6696293	53.2274	17.92428	0.02375238
0.0637736	9.82842668	12.5379719	18.394673	4.2729346	8.7538477	31.75825	22.87749	0.02873874
0.010427	4.02309286	9.47230498	11.0241753	2.2482397	1.6760739	22.44776	6.919665	0.19130817
1.4757042	14.6398606	15.6537673	13.8205028	3.4926319	5.6804282	42.47714	14.02561	0.0269652
5.3131152	10.9984916	18.6923513	20.9004603	2.8348789	53.806695	68.70498	19.42635	2.87972737
2.0055594	9.25469353	16.9395575	15.3501503	2.6246282	7.7545282	55.45248	13.51863	5.21910146
0.430554	7.79319995	15.7254877	12.9683189	2.1356255	2.5653063	39.86796	10.69604	0.31680387
0.381059	13.3485426	21.5434766	18.0104807	4.6142378	7.7526108	50.24127	18.71645	0.05222252
1.1074198	8.00787271	17.3645339	10.5959604	2.9971684	5.6383576	53.21706	14.69291	0.11778677
1.2334719	11.1878009	24.5301569	23.3437652	3.1090317	5.6035455	60.52809	8.056281	0.22714928
0.0923568	9.70643802	19.8529062	17.3981312	1.4793674	5.3162091	44.6978	18.40082	0.09884594
0.1565674	10.5241243	29.4302506	18.9884839	2.9035344	5.7940212	66.63633	18.2011	2.0804995
1.6751147	10.1936751	18.2851026	23.5191623	4.8952723	5.481794	55.47239	16.03435	0.43534586
0.5589842	7.37467863	18.1903476	15.6932428	2.3174889	4.6907541	42.54085	9.381406	0.31213539
0.4953988	10.4279782	24.363464	19.4918541	3.4636502	6.0558014	52.77998	14.01741	0.40888804
0.0255699	11.4035601	17.7103824	13.3911635	3.3724747	8.7860311	42.21023	15.20077	0.19938472
0.5535786	13.0201642	12.6439783	14.0322098	3.0147733	4.7265814	46.07135	11.9163	0.31332768
0.4133025	5.6893256	13.3283799	10.2162614	1.7927415	2.8694953	29.79889	8.879079	0.41742131
3.736372	16.7624416	20.942563	18.3787281	3.4385118	5.9201199	40.96357	14.93667	0.0159557
2.4602267	5.57360719	15.41479	13.2594447	1.9981484	2.8589087	50.05327	12.25463	0.92139055
1.4460167	8.23177389	10.6324528	16.52593	2.5412445	2.6004117	43.56051	15.99709	0.46565461
0.0137009	13.1835775	11.4431271	13.087614	2.6341383	5.1590407	42.57102	8.754822	0.01221965
0.6364383	11.6862387	11.2013669	17.0524307	3.1769233	5.0672727	45.70443	6.948781	0.0376676
0.4185657	12.5579347	18.7004026	15.9606741	2.8341476	5.5938949	51.34853	12.25069	0.09573658
1.1310219	9.66133366	12.7155667	15.3908522	2.054152	4.7113669	30.16152	7.336033	0.59863536
0.0942009	19.7753583	22.2559235	17.8416291	1.712772	4.1922857	36.18408	10.13849	0.07332338
3.8539933	12.7647729	13.3008092	10.594639	3.6333057	6.5972505	43.37641	12.12383	0.00893777
0.7439005	11.2018196	14.614797	20.912887	2.2400418	6.4413211	52.5101	11.45199	1.63583308

0.3353401	14.4865409	17.8393837	15.3593597	3.5417543	7.397807	52.44867	12.58573	0.00470177
0.9071051	7.78458291	10.9438182	11.3753195	2.4015938	3.9420233	33.01687	9.51907	0.17798736
0.0170806	14.467927	15.6474185	16.1855924	3.4554776	7.1632251	53.3454	12.1001	0.02376493
0.0741122	10.5549129	15.5209342	17.2924628	2.7491915	4.9835996	35.86285	11.84541	0.14982561
1.4313189	8.16358739	12.6107413	27.9430882	1.839809	3.8377563	26.24849	8.864225	0.34947974
0.1433519	6.26233333	13.2958387	13.8724004	1.4862562	4.1079487	26.58886	6.27957	0.21690994
0.7298695	11.4609405	22.7463427	16.0877833	4.2758168	7.3191995	49.97993	16.90932	0.0305585
0.3950311	14.5607485	18.8283903	15.5615234	4.6085485	6.9524644	51.20719	14.11747	0.04065258
0.0693702	9.6247572	24.1951642	20.873635	3.5973562	6.6964469	46.7811	16.93977	0.05810426
2.7736197	13.4040839	16.6684598	18.5670712	3.5126158	5.409707	41.8596	13.10978	0.08354127
0.0760627	9.78847223	16.904643	14.740921	2.7999735	4.6910339	33.64853	12.08534	0.02504833
0.0336357	10.4036615	10.4228769	14.4329809	3.8721382	8.7155554	24.25726	18.18705	0.1019972
0.0606831	9.98596195	17.2789612	13.8985976	2.4375077	7.574791	37.15277	14.21428	0.46665486
0.2360909	7.61300516	14.3614388	11.0485468	2.9053743	4.7584747	46.43824	9.996436	0.08698791
0.8702299	13.0159724	22.0461701	19.3952547	3.9625754	7.1224594	42.43337	15.37472	0.45918902
0.3083018	6.44305851	14.7531321	10.7916187	1.9569926	3.2431929	34.08451	9.795619	0.01399846
0.0216863	5.08926154	15.8431383	14.2425178	1.1706935	3.1079134	25.72517	10.03246	0.02707831
0.6417068	5.55129147	14.4602982	13.6925707	1.3470914	2.8494097	29.36103	9.348497	0.00266199
0.0684705	7.82480066	13.4505078	12.3232205	2.5516957	4.7752577	37.41371	8.909378	0.01357062
0.8728174	7.04079685	15.025693	13.408476	2.1775826	3.7983529	50.76448	11.08724	0.3992503
0.118328	13.4458763	16.9343482	17.0033455	3.4149313	6.8601595	44.28436	11.23186	0.00938089
0.7828573	16.9766243	22.3400981	18.1046384	5.8610396	8.978459	56.05305	22.08525	0.00960851
0.3316028	13.509717	12.835658	15.7965995	2.1843915	4.5842474	35.31104	8.707807	0.00266844
0.2541724	10.6601086	24.8305344	18.4542415	3.189409	7.3856525	46.32879	16.78821	1.49873713
0.9151463	16.6496651	19.1171149	18.2459757	3.8961667	6.6527684	56.9233	13.95249	0.02647152
2.1044583	15.8534605	21.2971494	17.811583	3.90892	7.6281295	53.11441	17.92304	0.04682332
0.0272036	15.6488508	18.3488266	18.8231479	6.1028519	9.1470613	44.41866	14.3741	0.01455748
0.950847	12.7216289	18.1446336	15.3156744	3.6081091	6.1968636	36.68802	13.61196	0.05669794
3.2159459	13.8481808	16.8253829	15.9244985	3.2595241	6.9417322	46.86322	11.48207	0.01565214
0.1602187	9.33695495	19.4151757	16.0344935	3.3460504	6.905433	47.34875	13.15647	0.14356127
0.9548656	10.923727	14.4968705	12.8760018	2.4283224	6.333585	36.89979	9.317204	0.93431536
0.0152041	6.77504251	11.3305737	13.3657626	4.3277721	5.9422234	31.89842	18.67096	0.47422449
2.2265365	12.2713103	10.2032462	18.076499	1.7130667	7.0825479	52.61409	9.855887	1.77430083
0.0377394	9.41304018	10.4805097	14.4026334	2.0013881	3.8479226	34.10164	7.123017	0.0121173
0.1491424	13.0899037	14.819561	14.5970178	2.7443391	5.5641912	48.55807	9.802304	0.06282973
0.7325763	12.4239844	13.7911478	13.5037532	3.144794	4.8105676	42.06211	9.27174	0.0675488
1.7059407	11.0225799	13.9147796	16.6525579	3.1423321	3.4314375	25.96094	11.7909	1.31508924
1.1001925	11.653159	17.5808671	14.6821198	3.5126269	6.5607649	40.54076	14.94934	0.05854944
0.6508585	6.94791286	24.662294	13.3545836	4.0005678	7.6036088	44.87006	26.69727	1.5403127
0.971063	10.3136145	21.3827604	12.5973985	3.0634029	6.4559294	44.79217	22.52934	2.48643326
9.2973986	8.72727297	13.2923336	24.3098412	2.0105825	8.0601317	80.92567	12.99777	3.23375427
1.3555309	17.3145665	26.4299133	21.2911048	4.780703	4.5915721	39.23544	15.48128	0.01632375
0.1243654	10.8217264	16.8353805	18.4664718	2.4370362	5.9471731	34.30268	13.40674	0.00211276
0.4721606	15.3563301	13.2542066	14.9382417	4.0304933	6.014222	43.51803	11.32569	0.01754637
2.1095097	11.0875601	21.7563368	18.8497513	4.5012518	8.4298915	42.93368	19.22733	0.01808813
0.9526329	14.8645181	14.4876519	15.7397622	3.1259523	6.2496256	38.82347	10.16047	0.02967554
0.9881618	10.2145312	19.1565796	15.5668513	1.9544413	3.5673846	43.73868	10.42891	1.27420733
0.8196052	13.5432583	15.8829192	13.3256641	3.6120328	7.9387166	48.18348	15.69158	0.08734754
5.5186095	8.73658989	13.9288691	14.6894622	2.4051273	5.6235839	62.90132	11.15695	3.33344534
0.1956913	16.7483595	21.6496156	17.1382962	5.7805714	10.296417	46.08925	21.23682	0.02750235
0.0585562	18.3907632	15.816243	14.8934196	4.0353369	9.1794335	38.93424	15.85786	0.0483458
0.0530364	17.6539736	14.6806183	12.7716617	4.1819639	8.1671118	56.51577	11.80792	0.02459722

1.0622137	8.40043711	17.8274262	11.9888174	1.8996719	7.1583474	38.19371	12.34481	1.3702897
0.0208418	6.03938676	10.9412194	13.49812	3.1318535	5.2154991	23.95112	10.58749	0.28328844
0.0255929	13.4905262	18.4641381	15.4613171	5.9137961	10.534523	52.22914	17.08682	0.02934757
0.0313174	14.4921094	17.2458703	16.180737	4.5289492	8.7164245	41.89293	14.36002	0.03746107
0.7735622	9.62527368	12.943371	11.862813	2.6983731	5.5744463	41.15235	8.111472	0.00499747
1.598041	10.7882068	16.8064522	14.5452705	4.2639172	6.9561547	50.64591	10.96534	0.00952295
0.0878168	5.91998564	15.373838	13.0150089	1.4717329	3.3524953	28.68562	9.228927	0.02741287
2.7224486	15.7875162	17.1019333	8.61773386	4.6462198	12.775469	46.47499	15.39954	0.01914992
1.2871141	13.0968001	19.414688	16.5676084	3.7323245	9.6735794	41.94363	13.20641	0.01679938
0.128029	15.4198249	16.5206433	15.5919469	3.4670379	9.9876648	39.64863	18.54441	0.02131494
1.4542193	11.9207279	20.4566935	18.2820969	3.3674356	6.2089455	44.88356	11.086	0.01086868
0.5730074	16.3481942	23.8564242	19.4050602	4.9973356	10.266149	55.58897	17.40877	0.01099047
2.7702377	11.6270363	15.3316107	11.0562769	3.1545025	6.9442527	38.68261	10.43661	0.01395895
0.1488943	16.7444639	18.3762534	16.7659632	4.0599033	7.2898744	60.45747	9.66951	0.02439121
0.0762925	9.52816707	17.0767604	14.6361731	3.0734723	4.8170555	50.27726	8.148233	0.05325192
0.1004966	11.7713976	17.3420573	22.1097279	3.0602242	7.7599324	40.52469	11.534	0.26563487
0.4989847	9.99756286	22.1901907	21.072455	3.5781508	6.6983021	45.20552	15.21047	0.01745243
0.7618433	16.8339382	18.8716946	15.5207871	5.5422776	8.136102	58.60901	16.58194	0.03026903
0.0671183	9.0420348	20.1088511	14.6522276	3.2668742	4.3919788	52.38511	8.362344	0
2.0213202	8.94721933	13.4569987	12.2726261	3.239127	8.3911888	34.8953	15.32558	0.76792673
0.2094201	7.7015717	33.4213272	23.4819802	1.0713013	0.5847039	18.91891	5.654509	0.00546669
2.19784	9.16581706	10.966565	16.9012642	3.2255977	7.1209209	35.57435	14.59107	0.02922068
0.0178118	12.6188673	18.3661899	15.3637903	4.583063	8.1097419	41.89933	15.79564	0.0508354
0.5471311	11.2759569	14.0512904	14.0259498	2.9850609	7.4526636	48.84263	13.15702	0.1288262
0.0708412	11.346313	16.8566026	29.6378802	2.7938541	5.5846992	42.0732	7.886595	0.03913219
0.1409359	14.3007938	14.9518055	16.7187228	3.4652516	10.708286	40.03132	19.8214	0.07039124
0.5167152	14.5998549	16.6450937	13.6085614	3.6893229	8.7962624	40.06804	13.48008	0.04472961
0.1492565	16.0703621	16.5503424	14.5232633	4.5122062	9.7270354	44.77184	16.301	0.03042735
0.3199371	12.9112451	17.8010836	16.4249992	3.4839589	7.0228026	43.51838	10.68378	0.04515383
0.0051632	16.2434269	21.5190229	19.1440856	5.412298	10.428021	55.90498	11.831	0.00736795
0.4833959	15.6479304	24.1720236	17.4228413	5.8386908	10.111988	50.88276	20.53419	0.01942308
0.041155	9.09538829	11.8462266	19.1105875	5.2355062	11.096072	27.50582	25.64559	0.35971375
0.0317793	12.233092	17.5469404	15.9968327	9.7413597	7.8326754	29.97157	28.89062	0.67740918
0.005709	14.2874704	11.6295551	18.4475398	9.8291587	16.439281	37.13269	41.01668	0.59878729
0.2730666	10.2781126	19.3368532	14.5644321	4.5336368	7.0530985	57.77823	16.68871	0.05845057
0.2348382	12.7487836	24.2517259	16.00924	7.1061916	8.3925021	53.84503	20.6521	0.18279154
0.0466989	18.1234183	20.9107553	17.8347791	8.3997382	14.126846	60.45181	25.75196	0.31487424
0.0834325	9.72679498	18.5186021	13.6800764	3.0332024	6.2381906	40.85057	11.53587	0.06337041
1.7267528	7.38582065	15.9540575	14.2387589	2.9197782	2.8791269	65.1316	14.43428	0.04126143
1.1188241	13.653223	14.6177225	14.8785594	4.4774895	8.3162998	43.70106	14.49526	0.04860437
0.0244519	15.5519938	19.3176546	14.2889652	4.496795	8.5908336	37.59519	16.45216	0.0221438
1.0471895	13.4584022	20.665308	14.8221473	4.0900329	10.621029	36.44888	18.4087	0.07247455
1.0217773	10.9803193	19.4895635	8.62937288	7.0616081	3.7447496	41.81417	19.58182	0.13485722
1.7122954	18.9847626	19.3516398	13.74552	7.2760922	12.678944	32.23317	25.45438	0.42407383
1.3646082	14.1561072	16.4019911	13.3397506	3.9134539	8.0137683	37.0258	11.91299	0.01018472
1.7762881	13.7002521	18.1903547	9.70967415	7.5088202	5.7434868	34.79173	21.39717	0.08223561
2.3159768	18.910823	17.5811863	9.79455995	6.990296	11.071848	33.26005	27.6011	0.21232829
0.0258085	14.6763777	23.5103552	18.7247662	4.585518	12.856856	43.52831	17.0979	0.00849903
2.013247	11.0207084	23.0346583	13.6672393	3.5337321	10.018778	43.44162	18.37264	1.15967337
1.8016086	10.8233332	20.2716322	13.5733837	3.6541492	8.9069301	35.09624	15.56123	0.09473782
0.2345077	20.2533187	17.0864225	17.9130964	5.2176952	11.368071	51.55162	10.71717	0.01332662
0.0013538	14.5462477	24.5414316	19.6309197	5.2617936	10.303622	53.92154	24.49078	0.02752875

0.0497827	19.1807665	17.1270626	15.2184295	5.6564042	10.65065	48.41856	18.87545	0.01165512
0.5611297	13.1203209	23.506921	23.3826879	6.3916272	9.0358088	32.18638	83.22058	1.71674033
0.9710888	18.1159509	14.9939798	12.7944433	4.2068932	8.4613575	57.39037	14.68773	0.00824857
1.6794151	10.0138531	14.0628024	37.5407851	3.9241649	3.7232775	54.45759	16.4532	4.63326899
0.0068514	13.4345379	20.3286219	15.4037437	3.7470884	8.6393797	39.14973	17.28324	0.5371239
0.0147066	8.70875998	20.9327255	14.0589026	3.1124235	6.3667377	32.31302	16.16269	0.05621402
0.8441786	16.1385361	21.1302496	15.2164938	4.2894285	8.5598916	45.89918	18.93693	0.02554863
0.115767	14.6936174	19.5474029	18.4165877	4.2824242	10.812311	48.84261	17.66661	0.0247802
0.0925599	9.81371469	11.1808978	10.5039826	2.8321342	4.5031176	48.78066	10.45531	2.07042404
0.4312674	23.4227465	22.9957043	12.8435369	11.494017	7.9183759	49.77499	46.57164	0.07581329
0.0339253	16.125434	25.0647503	21.9996698	5.5895474	9.8832088	52.95208	19.68221	0.02562982
0.0067903	10.071379	21.1680451	18.0819017	3.9841334	3.8202573	67.27775	17.61691	0.01695737
0.2558732	11.1479322	18.1301121	15.5525349	3.1812832	4.8898542	45.89134	12.05125	0
0	7.76023277	16.6618076	10.6138188	3.1369902	2.6974829	65.23815	15.99407	0.00914389
0.0449463	9.30284107	11.2459473	12.4567164	4.0078817	8.0202869	24.94388	18.01444	0.07320227
1.3240943	11.6684863	16.3859411	14.032395	4.9049167	6.35891	51.63168	16.27239	2.1404331
1.3471745	8.54623324	12.6483641	17.8319929	2.3391534	5.6819585	31.41154	12.5985	0.02929921
1.6393244	11.1212653	15.7213003	16.9753291	3.7245307	7.5906062	39.30764	15.25834	0.44312539
0.6719104	8.61951822	16.3586116	12.2371554	2.3856709	9.1284109	37.5534	16.51372	0.07428274
1.7084742	11.2098526	16.8870514	12.9404829	4.732488	10.790285	40.88425	19.2712	0.02868851
0.5315841	9.47232068	18.8044646	14.3412303	3.1863561	8.858922	48.74939	17.23655	0.18377944
0.1898156	10.293227	17.4066541	14.7825171	3.5078432	8.6289427	46.77895	17.58848	0.52447861
0.0512329	13.2635758	15.2617518	16.318777	5.5709434	11.526892	42.55521	16.34534	0.06266588
0.6773353	12.8645716	19.985252	13.1625064	4.7014638	10.794978	46.5142	20.90987	0.01933137
0.2995233	12.540363	21.9559245	17.1967218	5.2122255	11.156833	49.75582	14.42664	0.07577072
0.9363931	12.028619	22.1722207	22.515319	4.0236306	9.9409921	53.56045	17.60638	0.00782958
2.819117	10.503431	17.5768296	15.6258051	3.7298032	7.784545	35.1736	15.78243	0.03412661
0.7564223	8.19047593	14.2210619	12.5908343	3.2054502	8.0634527	33.20325	15.13604	0.05449029
0.2586027	9.14343233	18.3776524	18.4367586	2.6702959	7.2229757	44.49176	15.04437	0.89157081
0.8505998	13.7887562	22.2432384	17.7453857	4.5467592	9.8413809	45.84079	16.77386	0.03481318
1.0584443	9.19889954	20.4423416	15.815996	3.344039	7.6225391	34.54865	16.90424	0.02975865
0.0222029	15.4675748	14.9051071	17.0693065	3.2420691	6.194466	38.86646	13.69748	0.08079391
0.4029645	7.20526692	26.1687724	22.2194858	3.9450161	5.5297867	55.65787	19.38494	0.92673336
0.3281359	22.000868	13.9522329	11.65051	4.4255515	10.404926	49.24261	10.91549	0.01404765
0.1488925	14.9924294	14.5853515	16.3934724	3.903552	6.3948445	57.37223	11.37737	0.0159354
0.0060281	16.8684076	16.1997082	15.7983857	2.9347313	8.1187697	40.65826	12.41994	0.01129032
0.8695657	7.65760055	10.7005824	14.3695659	1.6433114	2.8463916	23.81363	6.31874	0.55249743
0.0782014	11.6592971	17.8783059	18.56882	3.2433937	6.4757081	44.99807	11.20589	0.00612409
0.0108743	21.9321854	13.9738313	13.5632907	4.6798772	8.3276795	49.62053	11.03208	0.00232767
0.0989462	14.1878565	23.1027946	16.6401954	4.1994453	6.702319	55.76869	12.77603	0.20591348
0.0966256	11.9273414	18.5357138	18.6579585	3.0533613	8.9048335	34.44063	12.78359	0.09332548
0.0103432	14.7327374	15.3959597	11.9761584	4.047663	7.0930944	53.30736	12.83535	0.01992597
0.3113019	7.97899795	16.34962	11.0094438	2.6526323	4.9155252	54.34672	12.3891	0.00489963
0.3608558	11.2346699	23.9894762	14.5588387	4.5465609	7.7625207	63.99656	21.51791	0.03662339
1.4177246	12.5115371	19.4181455	14.2793988	3.9545422	7.4161412	50.20176	17.09377	0.27623311
0.8900277	15.0785403	21.515269	15.1598906	4.0871743	8.9563742	45.84104	19.1979	0.03626508
0.153514	13.3598457	19.8454197	12.8779524	3.8141393	8.9205284	55.78897	16.12851	0.37241385
0.0284131	16.4372642	17.3169394	18.2213386	4.2507237	9.4096259	49.83316	19.73345	0.03649138
0.47319	13.3798959	14.7968522	13.3808509	3.5175769	4.5429333	77.78451	7.889689	0.01929285
0.0453753	10.7616338	16.689128	13.0504697	4.487411	6.5907036	65.42317	14.66902	0.01765944
0.1986716	5.65017796	14.4743913	12.8741093	2.1572332	3.3357966	33.13929	9.894657	0.12901185
0.0068183	13.940636	19.0169157	15.8796637	3.8701817	8.6012751	57.87754	14.81189	0.74286785

0.4300347	10.5145956	20.9048293	14.5736172	4.5688475	7.4039728	64.30519	19.02494	0.0278443
0.1134502	11.9482365	17.2848981	13.4474378	3.2926656	8.4520333	46.87928	14.7483	0.06806959
2.3217376	11.2192876	18.7711635	12.6228383	3.0060016	6.1124951	51.02036	20.37343	0.05916351
0.9527759	11.9374525	13.4812787	14.5391198	4.334316	8.4721998	50.61201	17.95607	0.27116145
0.2168822	16.2903792	17.887141	16.1364426	3.5933574	8.4363121	64.57186	12.65108	0.00174527
2.7338314	15.6232693	15.3672526	15.3583747	5.0255943	7.826646	47.7252	17.94881	0.03228597
0.0324842	10.7069688	13.1616995	12.1774698	2.7188004	4.7649335	55.04427	11.32606	0.43979762
0.1460922	16.3183401	14.7351033	15.2756108	3.5431707	5.4518856	58.78666	9.074743	0.01901639
0.0553655	10.0764394	19.5877864	12.4470079	3.9838717	5.2873027	65.66404	15.92093	0.01917091
0.857977	18.6332906	17.1584106	13.2763697	4.7140704	9.2102245	54.08075	15.78485	0.03148321
0.7397952	10.1215377	19.3810173	19.1923485	4.6466958	6.2877789	37.03169	15.13494	0.03268795
0.2342265	17.8457192	22.6198643	19.9647286	4.6663458	9.470321	49.90268	14.64164	0.03581195
0.0352629	5.45475469	9.13521751	16.8584197	1.7670766	2.4694425	14.98599	14.2372	0.01258019
0.1066358	13.3419487	13.3702013	12.7656072	2.9310075	5.0541936	39.43139	9.929383	0.05035072
0.4876602	10.0031454	12.2691593	13.6081431	2.526994	4.2770204	38.37901	7.12953	0.0335373
5.0148339	8.01561622	10.6438552	15.4604833	2.9523896	3.80948	46.2859	18.42505	0.9515784
0.4923311	19.8353439	10.5958534	17.2450221	5.3133623	9.1096999	54.89531	10.40143	0.00652537
0.466504	14.3605463	15.210364	16.6721365	2.7740986	5.3344705	46.14258	8.108626	0.0182664
2.6196294	15.0496737	10.7560509	13.9573743	4.1867392	7.4438129	48.30563	15.07272	0.49764651
0.7125031	7.35677735	13.6404422	9.6477478	4.3084128	4.5177474	56.7941	21.02812	2.09262027
0.0157217	16.9770208	20.1555488	16.9032768	4.6383196	9.0107125	54.52132	14.8745	0.02734276
0.0191907	8.79780235	16.7811413	14.2548136	2.9041262	6.3783291	52.81051	12.75869	0.06908596
0.0278663	15.1530047	15.8429013	15.6263906	4.4812282	8.1655768	54.01596	12.66788	0.03834545
0.0209292	13.1217012	21.4869968	15.4490545	4.4122246	8.5118977	41.84118	9.74604	0.01119987
0.520115	12.7179322	16.2221224	14.3284796	3.470559	6.6338952	55.78449	10.08327	0.00927766
1.4954464	11.7268035	14.8480645	13.2824282	2.3756129	5.7900374	39.39731	8.254387	0.04182926
0.0748797	9.38063658	9.8862303	22.8071449	2.3723341	2.056906	26.09655	12.42738	0.4612554
0.8204711	14.6883116	15.346229	11.5029001	3.7492479	10.307101	41.61508	13.31699	0.20233159
1.4724051	13.0398438	14.8857223	16.209889	3.4747598	7.2406819	56.74216	23.1225	4.00804487
0.1217159	15.0784186	15.9659564	16.1058188	4.1906264	6.8885622	57.48131	9.354689	0.00827098
0.7776516	12.1082502	19.624737	14.8019678	3.8630883	8.0048798	52.08294	14.09395	0.01664582
0.1750454	10.6551242	13.9203756	12.7597354	2.8048298	4.4771502	58.74136	7.294516	0.00497375
0.2083065	7.67378095	14.0633735	8.16891466	2.9967319	4.4874467	61.86656	7.484236	0.02175049
0.0232615	11.5846134	21.5660291	14.4228201	5.1068359	9.1681557	42.34114	20.34547	0.02872598
0.0320433	5.2763717	13.5034081	21.861167	1.1034365	1.3180344	23.72356	8.98735	0.00403468
0.9546987	11.0651746	19.7870425	12.7768806	4.6412922	5.747504	52.48798	21.38825	0.0670961
0.4795884	13.7750306	21.444995	12.2735452	4.6617915	8.4240666	41.32548	19.28155	0.01655759
0.1033141	16.8401942	24.9299985	20.7339494	5.1782575	7.035692	62.60732	18.4489	0.01340281
6.8307871	13.3038973	20.0743473	17.208261	4.7989401	8.6706514	47.05733	18.50113	0.04372835
1.1201861	16.7176307	14.1528873	13.5974386	3.6924313	8.1044612	39.66996	13.70795	0.01515454
0.0118241	13.6086769	20.458158	19.2070759	4.5648951	8.3572647	49.47609	14.33334	0.00632742
2.2614236	6.30213647	14.8947168	17.482196	1.5512502	3.5964677	68.10542	12.26866	0.27176002
0.8027529	11.0251528	11.8590886	13.7880128	3.2423956	6.8023366	45.88167	10.61494	0.06608892
0.8506052	14.862543	24.0242769	20.6516832	3.8519561	6.1614934	51.97656	13.06624	0.00741646
1.0329231	14.9537081	17.1505442	17.6086259	5.3135363	10.605921	36.63422	16.88737	0.03212559
0.0150139	9.79810705	17.025156	17.6078482	2.1245618	4.0768558	43.33921	9.71952	0
0.4273209	11.7785243	18.2677174	15.7110968	3.8754304	7.3182088	47.32345	15.92685	0.05060789
1.5942775	10.9685062	19.202081	18.4126882	4.7013166	7.3193668	42.01443	20.31746	0.64035765
1.7164648	11.5852058	20.2709271	17.3937205	3.2639059	8.1995665	47.12695	13.76068	0.05193802
0.0178915	12.6978628	21.721354	16.7751514	4.1483589	7.0227582	57.575	15.16996	0.02340381
0.6031263	8.20074489	10.6539826	13.0899929	1.9883901	4.413679	28.29601	16.18647	1.74965322
0.4846467	12.9787215	20.3401707	14.6329026	4.7842018	9.0181239	44.98046	18.4051	0.00891235

1.113233	13.1913738	21.9555073	16.8986776	4.5630858	6.5147854	39.76356	19.42592	0.00951258
0.0144538	13.651093	23.0141805	16.228668	3.9567382	12.140588	46.41641	14.87571	0.02234461
0.3336839	8.61887059	23.987713	13.124216	3.5493075	5.0859039	33.82037	22.62321	0.47974865
2.3028822	11.1356814	15.4880379	16.3123347	3.2114159	6.009356	42.75566	13.60658	1.23728491
0.5410983	4.47766782	12.4963553	11.8193474	2.0259292	3.566919	39.47327	18.92158	0.30758961
0.0212432	12.5847269	19.3470505	14.6989129	4.1335546	8.7813524	42.49859	15.47121	0.10799479
2.3557678	10.489096	16.6331024	15.1570854	3.5240968	7.5934878	35.84286	13.64388	0.20532873
0.2950022	9.18861459	16.0917764	13.3207968	3.1790984	5.3926741	37.35353	10.76963	0.20091884
1.6781098	12.2819119	18.6714302	14.148258	4.6000428	6.8442232	47.31785	22.55913	0.17140413
0.0802087	8.24747083	18.6946242	15.2643671	2.9812586	5.0375404	40.61899	19.41352	0.54910785
0.2611239	7.83294434	12.5794033	14.3342108	1.7593123	4.082012	34.21122	6.126569	0.16697949
0.0516856	14.5611343	20.5275561	17.2466231	4.2344906	10.133814	43.5639	18.9609	0.01985744
0.0853752	6.40075619	13.746094	10.8123488	2.3083908	3.8106281	31.88849	12.40606	0.04488542
3.5024254	11.7408792	16.5469883	16.0871981	3.5616199	8.435993	38.30487	15.81076	0.04291478
2.6369014	14.9096917	16.0840936	13.2211214	4.3137179	5.9510324	47.89623	14.55779	0.2425424
0.4985662	16.9258696	14.2020009	16.8268112	4.7495643	11.099534	46.94699	21.77154	0.03621696
0.5482532	12.1831404	14.4062025	22.0597291	3.2103673	7.640944	39.99225	8.942378	0.05212465
0.4410058	13.520512	14.719451	17.5049206	3.5849553	4.7254181	53.76182	10.27826	0.01537432
0.0131475	10.8592557	12.5455248	14.1673655	2.5022823	4.61425	36.43691	8.370409	0.01583017
0.2528124	16.4884422	16.5564131	16.7041199	4.0458974	11.808723	43.32473	17.9635	0.03523774
0.337587	12.219193	11.2618092	13.1119166	2.2505734	6.3373519	30.23636	9.610468	0.04955975
1.2178844	13.1643854	16.511591	15.3706726	4.3253736	8.2233843	44.44438	18.75652	0.06754269
0.2135392	14.8716152	11.4495713	12.738609	3.260693	7.4119833	39.16758	12.51744	0.02770217
0.3594063	14.9169884	20.3260823	20.0243063	3.1384475	8.9517795	44.33169	14.80515	0.01570037
0.2399848	14.4020547	13.7464247	12.7652727	4.7133062	7.9098958	55.99179	18.22318	0.04525392
1.854826	14.8122351	17.9845329	14.950018	4.7394057	9.0570667	49.12033	22.20691	0.02639827
3.1843164	20.5232869	14.4612605	12.8656038	3.3716543	7.2100127	44.88005	15.81438	2.04512961
0.8061153	10.3140189	11.1084751	15.1128623	1.6620954	3.8250505	42.91575	7.140424	0.00798847
0.0679633	18.5511966	16.3600739	15.2884578	4.6823263	10.40581	44.62034	18.62556	0.01600247
0.2774422	12.2988509	18.0764552	16.1992417	3.0007442	8.5343924	52.83183	21.68676	4.16722451
0.6074602	10.5573357	19.104787	15.1861754	3.256225	4.6692058	48.93675	15.82327	1.11917237
2.1495445	16.4083892	12.5446657	16.7173946	4.3551274	10.919421	43.90357	18.07096	0.03979705
0.5355328	15.7824163	16.5233472	17.4922391	4.3181132	8.9361746	45.53107	17.48636	0.02211005
1.6210446	15.2836164	11.1408485	15.8182777	3.6701134	7.4279106	41.88765	11.44094	0.05222781
0.4315427	19.0299558	21.2458716	17.2796726	6.0506595	11.032054	43.75137	18.71797	0.01965378
0.0706715	8.86912374	10.2494283	23.5624742	1.4872159	4.2663125	20.30149	7.978399	0.12235397
0.3099032	12.168572	16.9802414	15.5212905	3.481326	8.0799382	51.65257	10.55499	0.01451089
0.1685574	14.3279901	16.0882496	23.1600759	5.8607152	7.5749139	37.49569	15.41404	0.20359491
0.2200279	14.8781625	16.0887425	20.6773445	5.3650828	8.2419187	44.09338	16.49639	0.16444654
0.2103012	16.2876437	14.1541219	15.6495264	3.4344698	9.3100263	44.18154	14.80319	0.1006925
0.0037725	18.0484272	16.2661302	16.9719054	5.0796022	10.442454	43.95016	16.63077	0.00942098
0.8904975	8.37960653	13.2659155	14.4299989	2.2004843	4.5352004	33.6753	9.951744	0.03085464
0.146117	13.0873457	13.655422	16.0061728	2.8099224	4.4818657	55.00421	8.658407	0.02057677
0.5402845	14.7138313	13.0507528	16.8755363	5.0819003	6.4610248	37.73437	13.48976	0.04405684
0.7446263	11.6074101	23.339733	20.1752371	4.4401862	6.4115721	55.19052	18.02783	0.00158754
0.2341951	16.7070436	16.0314078	13.7782939	4.1207309	7.5888633	54.57791	11.96118	0.01474412
0.7651244	17.6338089	18.6491348	17.7266626	4.0252649	9.7980214	53.18472	18.07717	0.05278255
0.3486585	11.5448072	15.2933891	16.8250268	2.7559345	5.5192329	33.14407	9.501519	0.08635014
0.6676864	7.34273773	14.5352306	15.629347	2.5132748	5.387235	29.75964	7.543317	0.01713081
1.1714105	14.2781472	17.5341733	13.0491533	4.9719687	6.735597	62.22873	13.56978	0.00462626
0.4341603	7.8681831	9.77967601	11.7237846	1.4984541	3.7315866	31.50642	5.219119	0.09957113
0.8361868	10.6784608	17.9403575	11.388282	2.9003246	5.3690509	46.74782	9.421022	0.03553107

1.9008467	12.5461531	14.4783575	17.8933585	3.1201488	7.3992095	56.21969	12.74308	0.02154709
0.0139257	10.6677182	17.2367203	12.7819407	2.8814639	5.5500349	45.71575	7.700377	0.02794526
0.0964624	4.88001935	10.6023205	13.120492	1.406217	2.4496944	24.92455	1.692728	0.11825679
0.0648926	9.75472105	19.7875578	16.1853827	3.3947773	6.7478724	52.54956	8.74544	0.02209838
0.0972703	12.8470077	14.6118906	12.4061045	3.3804144	7.9600754	58.5324	7.447124	0.03539561
0.4549011	6.62479868	15.9425126	12.8781346	1.8938365	5.3657544	40.57037	7.366814	0.3881967
0.0615444	12.2976846	16.5461958	12.8547967	3.5339366	6.3449688	55.30535	9.810056	0.03208983
0.9029282	14.2502297	13.4558339	14.0743101	4.3757167	8.1595781	64.62691	13.29213	0.01157329
0.0177066	10.9808212	18.4422895	16.2054538	3.9135278	8.2569311	47.35066	14.1539	0.11844162
0.7987674	9.41168893	19.4373299	16.116219	2.6604598	8.5296975	30.95041	9.601104	0.05995335
1.0500537	11.949927	19.2224901	14.6744812	4.618414	8.4501967	48.44821	15.70978	0.04099818
0.0240667	5.28591922	6.98743357	11.3120543	1.586725	2.458361	28.09072	5.08269	0.00286196
1.0554836	15.0487052	19.6697816	17.7181459	4.9062116	9.3490244	44.68041	17.19661	0.01694466
0.0461947	13.5236137	17.4028865	13.1636951	4.7094879	10.808351	42.86751	13.86012	0.03955238
3.3786117	11.7347828	16.8748498	11.8089375	3.7768831	9.5826149	36.37753	15.70713	0.03079463
0.7509981	8.62996916	12.5019642	11.4103145	2.7361674	5.0260138	35.87874	6.675809	0.01150976
1.0691045	6.13935614	8.88036189	9.51588014	1.8101029	2.6567571	32.77531	4.298895	0.27406513
0.0380444	20.7125502	19.8986663	15.8698262	5.8435341	9.781966	62.58014	12.77362	0.00169656
1.853458	15.8569266	17.3064129	12.8064683	4.9681826	11.17383	58.98603	16.06706	0.04247721
0.5278051	10.9209919	18.3893485	14.969659	3.857654	8.412574	50.25466	12.11237	0.04512433
0.3851905	11.4027475	18.3360425	16.3502167	3.2885104	4.8490389	48.84529	9.820717	0.00808343
1.1846224	9.32267434	20.5994262	16.1440282	2.4585917	10.413923	41.85907	13.69698	1.47474511
0.0201763	15.6773519	16.6102081	18.274498	3.0470936	8.3350518	49.97496	11.57048	0.05315422
0.0414275	13.7086265	21.2140123	22.2434618	4.2661339	13.525108	45.60797	14.53943	0.07255351
0.1335202	13.5969408	19.4493168	19.1324814	3.6110412	6.6750589	48.3349	8.340786	0.14806671
3.4769592	13.32649	24.9225039	21.0181302	4.5064663	11.387699	44.07369	17.14394	0.02122102
0.5991437	18.2822371	20.0951155	17.0913645	5.5545693	9.9513025	49.90137	17.39479	0.04742295
0.8072844	13.1859658	15.9352397	15.1899551	4.788865	9.5904233	44.08586	15.51877	0.04320029
0.3187039	11.3545859	16.6917124	13.7340124	2.2743735	6.4472826	36.41621	12.61207	0.68475803
0.2604677	13.7532179	21.0047568	15.0230939	4.3900092	7.8036461	56.371	16.01432	1.41897824
0.3164732	9.5654539	13.7478098	11.0464452	2.2516107	2.9955559	29.39058	6.794711	0.02971134
0.2577859	18.4722081	19.3472854	13.507595	5.8873827	7.1096052	46.93845	20.52264	0.02508167
0.6260121	13.3098561	17.9632347	18.8814246	4.8699579	8.15115	63.68502	9.921935	0.0205865
0.4094703	12.4567516	18.3110367	13.5816347	1.8888488	7.5215006	42.26143	14.99995	0.07888329
6.5277028	4.86946006	14.7488542	14.5085087	3.0345182	6.1081303	44.2218	4.12951	1.54950228
0.1059755	16.1117249	18.8071474	16.1570805	4.5040327	10.762991	49.24168	16.77281	0.01323251
0.2349525	8.87034177	20.7522798	14.5627929	3.8279703	6.0231348	53.76527	17.00698	0.12680496
0.3420202	9.83254852	18.0517556	13.9178055	2.8603044	5.5338775	39.59579	12.00419	0.7443041
0.0774527	9.80278222	13.0916179	14.3617019	2.3546668	4.7491324	37.29166	9.988613	0.11865324
0.2406609	8.73405599	16.4835609	13.8902036	2.9100993	5.3712702	52.83032	12.29706	0.00865782
1.3352492	8.38120888	18.7513535	13.1086794	3.5104839	5.9658899	39.20632	17.67206	0.07043389
1.0361221	10.2639441	15.3367937	13.039063	3.3904813	6.4360695	41.89762	12.18861	0.44961408
0.0770653	8.0619397	14.3896474	14.5708155	1.9825179	3.7211704	29.03187	8.90019	0.17349252
0.2285485	10.639275	19.6761006	13.2192796	4.0372092	6.3078797	57.85484	17.78093	0.00577811
1.3014406	9.80315619	13.8444505	13.7186983	2.4791126	4.2730486	32.63631	10.52822	0.00232147
0.3245654	13.4354919	16.0647774	21.161285	4.4877577	6.7245066	46.75077	10.02032	0.0194182
0.0308029	14.427526	25.66624	22.5248898	3.5096806	7.4394068	61.32288	14.2424	0.00549453
0.7465764	10.2691875	16.9975928	16.3164102	2.3282997	4.5668346	39.31159	16.949	0.55399577
0.4550891	10.0836555	11.3075553	15.9435597	1.9960376	5.0042961	34.70318	10.05795	0.46997456
0.0395642	11.0003081	17.325629	17.9223466	3.1574851	6.3560695	37.29835	12.63559	0.17445751
1.3632056	14.7341971	18.4212373	14.9405932	3.7414596	6.1955605	57.62777	11.31923	0.00896184
0.3952488	14.5868223	19.4891505	15.5106698	4.4773964	8.363742	49.48609	17.98891	0.01626999

0.0038743	9.24176226	15.4506	13.208672	2.7257567	5.3598063	41.8116	8.317368	0.03939172
0.02645	9.44633805	15.0759877	15.6838088	3.1723997	7.1905054	44.6331	10.1436	1.06073338
0.3083915	13.9979386	20.6433312	19.2433348	4.2168097	9.9941919	45.40762	13.37912	0.04578863
0.4032161	10.4848463	16.4986471	14.0446436	2.9345106	7.2382948	44.0631	14.44679	0.32240075
0.1421096	5.89289563	19.4961374	15.5819277	1.6841608	3.4033383	37.18611	10.41596	0.04947653
0.0487628	14.8907008	17.0266368	14.2632895	3.6656615	5.6092485	46.30128	14.43578	0.00579877
0.4011682	11.662798	21.4854922	14.2234052	3.8669153	7.5337808	42.84924	18.25737	0.01951614
1.0283508	19.0339208	15.7178666	18.3186178	5.7486959	10.846605	45.69364	12.90926	0.03813977
0.2512447	12.3068625	22.1055406	16.2534472	3.3647482	7.1506022	54.63678	13.05691	0.05638177
0.4536971	12.6559313	16.0639801	13.8181977	3.6536202	8.7823278	36.15894	14.87561	0.0232456
0.2960957	18.6201842	20.3405604	18.8536157	4.723994	10.690516	47.0133	17.28448	0.04590608
0.1594045	12.6480458	17.3665898	18.6542638	2.6971851	5.2536854	53.89877	9.14866	0.26912269
0.9515261	10.706932	14.410251	16.643006	2.6215	5.1266446	39.29624	13.42443	1.44779676
0.527161	12.0525743	18.57178	15.0850655	3.2434932	7.096441	45.95123	9.714069	0.05783054
0.7317721	12.5719063	11.5594697	16.5701461	3.2684585	9.1712992	43.85405	8.779799	0.04146289
0.0117208	12.2321526	22.3052113	18.8955225	5.082536	10.549894	61.15405	15.70466	0.02508856
0.0464203	13.4900674	16.5900628	15.3194074	4.0592792	15.36487	55.67204	15.58303	0.02018328
0.0880221	13.568459	16.016075	13.210967	3.7766818	9.1467468	41.42735	13.88519	0.01916069
0.1916706	16.0132889	18.7990019	16.6793635	3.9747831	11.019396	46.73836	15.21076	0.02099835
1.800047	11.0267034	11.3304848	9.10647815	2.3043608	5.9889843	32.90814	4.220912	0.34314639
0.1225318	12.8742231	17.5073993	14.614404	3.8173847	6.7105591	46.41001	10.53532	0.01457124
0.1926532	12.4535101	18.0451032	13.6198269	2.9647572	8.6779818	44.45005	14.84701	0.16239698
1.8520244	12.3079256	19.0371076	17.4502966	3.4933532	8.136519	41.83231	13.30348	0.34094698
0.9355886	7.76375853	13.4893649	10.9749692	2.1417341	7.3957949	32.48374	9.476503	1.34054938
0.7144933	9.1784528	16.8971215	15.0066362	2.5733636	6.4575371	32.9938	7.041535	0.05771283
0.0310708	11.6406274	19.3784106	17.1811292	3.5264602	6.1867987	49.25743	14.93267	0.02955905
0.0480129	8.10301782	15.9259003	14.0565656	2.3824758	6.1109056	37.18736	8.434551	0.19820472
0.8399615	8.27099433	13.0406343	15.9987901	2.3025442	5.7569125	40.49691	13.85472	0.85999878
1.0172145	10.6605502	12.8420296	17.5467356	3.8951164	4.8073803	63.11094	12.32699	0.05911417
2.131267	4.97461645	14.5992824	20.252511	2.3163952	3.3181624	28.5178	9.347228	1.05037215
0.4221252	11.777537	19.6618781	17.5931077	3.7461492	7.796905	40.22038	13.84159	0.03115756
3.14231	10.576855	29.9726932	22.2023366	3.0777393	9.1327087	56.73572	32.65664	0.75799295
0.2776512	10.9774314	15.0453268	14.0109062	3.0767556	8.2142068	39.87456	14.04187	0.61240712
1.9043185	10.1788208	19.6695286	16.2413976	3.3823604	7.0988817	53.5453	11.70829	0.50842943
1.107313	12.2105643	22.5275164	15.210066	4.4951572	6.9975428	55.30667	11.43399	0.01012919
1.1833458	12.6461245	22.6144662	15.3097183	4.1425842	7.8702336	40.02678	14.69834	0.02922669
0.1503137	8.99035699	14.8318858	17.205922	3.0989468	8.4329596	45.42447	10.99309	0.10923613
0.0452483	11.0102405	13.8068226	17.086788	3.8395499	7.2012266	62.3866	9.103081	1.91475202
1.1010564	10.8249393	19.0610842	13.9387235	4.3124834	6.6742273	52.31677	17.70988	0.79695127
0.5410731	15.6517413	13.9460363	18.1263564	4.1872934	10.644771	46.30493	11.42278	1.14089378
0.3198462	8.54130859	15.7423592	11.7861694	2.9851349	5.3883362	41.9057	11.24387	0.06495287
2.5629627	11.9338416	29.9933781	19.6955796	5.0000224	9.0249845	45.23719	24.96111	0.01427841
0.0349302	8.20416785	15.774424	15.0497948	2.3963989	4.9735343	43.1922	12.11365	0.12914642
0.1174728	13.7722593	24.8317352	16.9272284	4.2229235	9.7257978	40.40386	19.15079	1.14310082
0.3500043	10.9047158	19.4500621	13.2892082	3.3859923	7.6152738	43.55214	17.2128	0.76509525
0.7387054	18.07598	18.3564794	14.2174251	5.3829482	10.036026	43.00147	19.48225	0.02168335
2.8796643	13.2473606	20.2638682	15.0086765	3.729675	7.0711487	34.44854	17.08942	0.01686198
0.8709207	17.7547995	20.6255121	16.151188	4.9154683	8.5696836	42.89041	19.20122	0.00647301
0.8515137	11.6345975	20.9216856	14.0707537	4.1475537	8.399063	39.23805	18.71076	0.0214014
1.0874564	7.58716996	18.8497652	12.7667486	2.8742572	6.2175004	33.87442	18.56947	2.47476274
0.1980197	14.2845721	19.2611036	15.7330282	2.6831803	7.4382442	44.52865	15.08109	0.01999368
0.7469858	10.8259183	18.2559985	17.2287698	3.8741731	7.7739909	47.04345	10.61996	0.14321581

0.7684054	10.5245927	23.395093	18.0932706	4.5849303	9.1914122	46.88314	20.4342	0.01461108
1.0501245	10.3621327	20.3457927	26.154932	4.196389	10.09399	53.44193	17.37789	3.86252749
0.0059297	17.7318114	20.2917931	19.6394028	5.9153488	13.66254	47.04471	13.15499	0.01110605
0.2297026	12.8694655	17.6592979	14.6962413	4.2769574	5.665519	45.74094	12.73251	0.0439585
0.0017133	15.1245113	27.2732652	22.3761484	5.5424166	12.878642	59.53335	22.5292	0.00550092
0.3298264	10.0138914	16.421444	13.669031	2.8350839	9.3495895	44.19077	17.77827	2.19862782
0.2829017	15.0592984	13.5499176	11.9664225	4.3618995	6.5954651	45.81786	13.9888	0.01137198
1.5217432	9.59222784	14.2781338	23.8061273	2.6662854	5.1972386	33.88533	13.37502	1.99728425
1.3085717	9.45310847	17.8519302	13.1760432	3.5703431	7.0703736	49.62547	16.38012	0.30293263
2.7294874	9.66381519	16.1997166	15.0879238	3.3628143	5.345267	36.79632	12.70844	4.16257175
0.7390751	8.11393157	12.7815311	11.8383427	2.0851725	5.093783	53.87696	13.27715	4.501574
1.8810949	16.9450476	28.740533	20.871263	5.667668	8.473201	71.56843	21.99696	0.00433271
0.3510337	9.10920357	16.3551645	14.5571482	2.7545179	5.8661326	66.22354	11.12339	10.2755179
0.0379275	11.3938647	18.4579591	10.4523123	3.5987196	5.3074451	52.89489	19.18136	0.88067742
0.867244	16.4154767	21.6957186	16.9468636	5.2581363	11.859295	54.38415	14.03138	0.11993315
1.3683731	12.9401777	13.4525622	14.5835556	4.6834143	5.454931	45.54469	17.63293	0.02587487
0.1116802	19.9231701	11.8221696	14.9624233	4.8941658	10.536706	46.54272	15.34468	0.08274949
0.2068204	12.7135784	24.3162793	19.0387681	3.9839866	8.4681208	57.38947	9.850565	0.01492261
0.0175752	12.7348719	16.7238146	14.9525753	3.7474239	7.0347418	40.45977	21.2584	0.01763442
0.0732052	12.8993897	14.4992854	14.501668	4.2669356	7.3051716	43.46586	12.03951	0.00949681
0.0107046	12.3572085	19.5024447	14.1836102	2.9011783	6.0635767	48.73622	11.20691	0.02673231
0.7325455	13.914104	18.8659758	17.1969063	4.5649229	7.8668138	62.05787	9.123147	0.00488991
0.0038362	15.3253868	10.6006967	9.2627185	3.3236605	6.3193578	78.41623	11.95238	0.05474369
0.1723109	15.4042495	18.6199271	14.2744259	4.5352647	9.1585427	50.31134	19.69627	0.31796178
3.0255407	8.29196176	11.5101342	17.316194	2.3009885	7.094257	33.19827	16.29564	2.69093876
0.0099432	10.8413581	15.0848512	13.2878277	3.4600723	7.6285111	60.03195	11.32029	0.02128371
0.3402595	14.3764929	17.1033998	13.1797032	3.6384128	8.1371003	49.41521	14.10924	0.01342551
0.6121962	12.1776597	14.9028543	12.9738507	3.0898743	5.6064524	51.66466	9.028817	0.0164981
0.0405493	9.84758245	12.9980599	14.6044459	2.542803	6.8165759	51.61078	10.0111	0.01215153
0.5525831	10.5549331	18.203471	14.3498515	3.7913972	9.7651103	52.71094	17.86213	0.97170336
1.0118818	12.9127906	19.4700231	18.1434301	5.670299	9.3315141	39.61065	26.29591	0.04666624
1.1482566	16.7362373	18.9728646	14.0108557	6.095567	11.714916	48.21886	11.20505	0.03795939
1.0086494	10.1479353	15.7235346	15.2022311	3.0234068	8.3888879	44.6714	15.32768	1.18398877
1.232101	14.5775777	21.6234434	17.1309686	4.1412608	8.5614637	59.57924	17.86425	0.01365855
1.9989971	9.83105239	17.8185049	13.8309297	2.8574707	5.9910998	38.79662	18.78891	1.81551124
0.7357634	15.7876363	21.1576564	16.6173439	3.9196521	8.5135924	63.11514	16.66575	0.00346136
0.4169067	6.24509081	13.9268397	20.7493377	2.0100398	4.0236462	37.44294	11.8112	1.56348257
0.1461842	8.65290897	11.504852	14.08915	1.8056398	4.2254429	45.05974	11.4434	0.2881069
0.1441587	10.8536698	17.1216538	14.9337801	3.1145511	5.930199	52.33816	13.32983	0.81237135
0	11.0872371	29.4415071	22.5641515	4.8146946	13.248532	61.09963	23.34813	0.00964675
1.3912956	10.4710818	16.8130838	16.4636649	3.2728006	5.3729844	37.84235	12.87576	0.1780206
1.1521957	14.9879275	20.4569292	13.7344925	4.4490313	9.9160944	39.691	16.743	0.07436957
0	22.5196057	18.4849749	16.8332005	6.5782204	14.790272	58.37021	11.51258	0.00597599
0.0983975	9.77334635	17.0151321	16.317751	3.1390521	7.2866925	38.62054	13.02328	0.97157966
0.5851719	12.7967931	20.8168204	13.6797802	4.547151	10.04988	45.87948	10.63165	0.02658767
0.1023616	21.8484338	23.7210541	13.417016	10.296345	15.546256	42.33742	41.61662	0.05203803
0.1886377	8.15115011	20.2996673	20.9476007	2.7017718	6.3380695	47.98388	9.913865	0.00212518
1.4605968	9.29521398	26.338071	19.4501695	3.9858453	6.4647471	45.97686	13.45666	0.00544676
0.0080808	15.9609574	19.680932	15.1452717	5.071678	11.024346	42.83543	18.92811	0.01729714
0.1321225	8.043894	18.5695866	14.9897562	2.9515074	4.3815532	50.71072	9.953641	0.0101004
0.2581934	11.2320972	22.3127986	15.3231871	3.71064	8.8814587	56.35158	14.66032	0.02686587
0.9567272	15.0236354	21.3398389	17.742318	4.3043875	8.6012191	55.49647	12.82686	0.29947749

0.177168	12.4168838	17.0146553	14.7644546	3.0932118	8.5944805	43.60264	12.20253	0.08668168
0.1648499	15.8547304	20.1189909	16.4661756	4.1875045	10.413338	52.23953	9.860322	0.02395998
0.0664269	14.906516	18.8116103	17.5247178	4.1072743	9.8455664	42.28839	14.16633	0.0056127
1.1456826	9.22272	12.0654881	18.2171604	1.772544	2.130485	26.78266	11.4231	0.74313976
0.0044437	10.8045166	18.5607585	18.804344	2.5365164	4.329755	54.83565	9.901137	0.01426792
1.1268034	17.9778117	23.3211712	22.1261437	4.4594726	9.1813468	56.20411	13.70964	0.02496842
0.1836714	19.4408678	17.9140332	17.0651888	4.4113026	10.832761	46.44636	13.8729	0.00607969
0.1397276	15.7078864	14.5204676	12.5091813	3.7659925	9.4547884	44.02399	13.35317	0.00946488
2.1572938	9.26256307	11.915878	12.9180479	3.270075	6.0172567	33.86092	10.10335	0.00618171
1.0853396	13.4987129	19.3899221	14.9888375	4.6650927	10.091802	45.18697	19.40021	0.02042669
0.918479	8.58030249	11.834889	14.536173	3.360082	10.578789	41.99272	18.646	0.04336823
0.0134005	6.43999801	12.5892245	16.9877614	1.25587	2.2070375	31.93828	6.175536	0
0.5228733	13.3830182	14.1414786	15.9495939	2.6760138	4.0668966	50.20786	8.389222	0.00337115
0.0077154	6.42128846	12.2201251	16.1380699	1.2648649	1.5404637	30.49062	5.87277	0.08257476
0.1762592	16.633863	9.60248377	22.4643394	3.7779371	5.2345103	52.31294	8.968615	0.01006099
0.0950573	17.6609153	14.9652576	21.3827534	5.2546791	8.6340707	53.0268	15.97139	0.01090031
0.4980948	14.2800551	14.5628883	15.8682891	3.3164886	6.5434929	44.19559	7.833228	0.04795007
0.0053279	11.4117238	12.915808	15.4813476	2.6734188	4.4205731	49.03639	10.41822	0.00570224
0.0740723	9.24004713	13.7894031	17.2650004	2.3467553	4.6011442	33.55316	8.178152	0.19290691
0.2571829	15.5422028	23.4248373	20.956318	4.8529433	5.839417	52.54936	11.79943	0.02274819
0.0715919	12.1053507	12.8357006	27.5655877	5.4866939	9.3021396	45.47796	19.06766	0.00170271

RBMX	FTO	WTAP	RBM15
29.2852	8.927291	17.092162	2.63468237
21.41035	4.307182	14.909452	2.14979926
34.34271	7.854573	16.575344	2.04841609
41.01977	14.29238	24.50153	2.724534
29.9218	9.968618	18.724803	2.71063193
25.71845	16.08871	16.284741	3.09869755
29.15226	5.509174	7.1797147	2.07825651
30.92681	5.337254	14.392473	1.98849553
32.53826	6.650798	11.243027	3.94958845
16.8075	7.638653	10.823735	2.43863359
42.78228	13.06933	12.158672	3.99461582
41.90186	8.85785	13.228429	2.99722343
33.17444	9.283298	15.590357	2.20088463
27.8326	18.19879	13.986263	2.26912218
43.9642	11.94292	16.521027	2.11314109
34.872	10.69575	17.399244	2.06522665
29.81693	3.862034	15.125033	0.99213418
43.6714	14.30401	20.835417	2.49097012
32.26326	10.87328	17.287201	3.80789656
25.13821	10.61985	13.192605	2.26846251
37.91361	10.58266	18.599332	2.04273242
27.1838	9.716988	12.879361	1.82952641
26.33907	11.91087	12.55467	3.11131055
32.80375	12.18078	16.354625	1.7663364
39.49437	13.36013	19.068578	2.98773794
42.56578	18.22713	13.081955	2.70832412
26.68628	11.50047	14.376524	2.55375892
35.94001	17.38017	16.962167	2.37518961
36.84635	10.40024	19.054	2.84459018
37.83001	9.455829	17.825345	2.07447657
32.08947	7.085089	19.699383	1.70374208
47.50988	9.828146	29.494301	2.56510809
44.95703	11.8422	13.565181	2.7632621
32.35722	12.67476	17.028496	2.96499028
46.87248	13.46893	14.542393	2.90907165
23.73658	9.391943	21.64843	2.67757554
17.71368	3.305981	10.150481	1.44028463
32.02139	10.39325	14.011314	2.57152692
21.54406	11.86295	28.297688	2.90505258
44.48478	8.570007	18.110241	1.58000393
41.83174	17.2336	13.185377	2.00159616
35.20825	8.505982	16.159617	2.82488412
36.96593	10.14426	12.674298	2.55346414
30.01437	16.36952	17.340211	2.7518171
28.29805	8.496191	14.598006	2.09186031
29.77493	9.170068	20.384739	3.79277762
31.63244	9.540948	13.480217	2.65877263
36.69461	9.307657	15.001708	2.75561406
30.80728	9.20912	9.5896419	3.69357236
24.50287	11.13931	15.669331	2.51753617

27.66563	6.321759	12.755225	2.21709157
36.05772	8.630711	13.235995	2.71578249
21.37119	7.430609	16.779628	2.89200494
25.21974	6.574372	17.178886	2.06120308
21.03501	2.088491	10.443497	3.07874359
40.46401	2.112169	19.992073	0.4250212
48.52062	9.274806	31.456346	2.6321234
25.84362	8.948753	15.095342	2.72356729
39.99892	11.54489	15.733777	2.09375968
23.00878	2.485756	7.6158224	1.64221267
30.60469	6.741837	9.6980085	2.2732869
40.52723	5.382771	8.63859	1.20469801
21.15146	2.810565	15.426164	1.46575571
27.22197	3.860484	14.105742	2.25821244
20.91563	9.886515	11.998438	1.59415391
13.61066	0.702754	5.3900837	0.73888312
24.09707	10.6143	20.169869	2.14987538
24.18069	2.922496	15.880984	1.90261457
21.69878	7.570153	12.883134	2.31969631
18.99754	2.878396	9.1808246	1.759408
31.68133	11.99894	23.528654	2.4434279
26.07955	5.554022	13.255462	1.72788961
37.24548	4.727108	22.37014	1.93011141
39.61369	8.515511	20.284116	2.18442864
35.42647	9.339238	16.844453	2.75602431
23.38824	3.074745	7.5492093	1.15554319
10.99185	2.450718	4.9014285	0.82515269
31.62088	7.460109	14.961697	2.11668676
34.25435	4.075902	15.058355	2.19426779
29.98482	7.199689	18.006181	2.52147278
22.35452	6.416914	17.232264	1.49966223
31.98864	11.15727	18.812562	3.47161038
23.25603	5.658322	10.123705	2.15335323
29.88624	5.938587	8.1848355	2.59587249
25.63976	3.47364	17.335421	3.77103825
42.21032	3.422753	15.588278	2.20122871
29.33658	4.995592	14.961503	3.24557346
20.98588	4.413836	12.569031	2.55987784
41.35002	11.42038	21.164437	3.41068592
28.57009	9.83333	12.327854	2.03775248
31.90088	6.234626	12.206061	2.30490355
18.15578	12.87594	8.8086931	2.27087653
44.22354	15.63001	15.679189	4.10740939
27.23877	10.19346	11.570666	2.73522348
17.38358	6.455334	14.471568	1.97685075
36.50484	4.974872	14.164181	2.65459874
36.16754	5.306192	17.055918	2.18531358
26.38803	6.960102	11.544891	2.1628736
33.65609	6.585396	16.273761	1.66920068
27.80151	2.695672	12.421341	2.87305968
39.44435	10.94585	14.267904	2.55736502
27.16691	6.319016	16.658666	2.83652375

27.01574	7.180562	28.764484	1.84802638
15.74116	5.117945	16.105965	1.39581315
27.18411	9.989717	16.834374	3.07842746
23.33693	4.110709	15.008383	2.63417537
18.83947	3.932751	13.953764	2.2929164
19.62769	2.712682	10.218037	1.78373788
35.53375	8.606075	16.076766	2.30775257
29.03872	7.228266	10.096934	2.71164531
16.55331	8.389143	18.609498	2.46203748
34.78138	10.10952	15.712357	2.35484056
25.63346	6.823873	17.355671	2.33957162
25.64226	5.366138	11.085894	1.45065739
21.39851	7.32144	11.06859	2.84951617
19.67732	3.811459	8.4902161	2.00432358
30.20948	17.68416	19.177786	3.38693323
19.96002	8.362244	9.1173015	1.69472392
13.31039	2.238364	7.9400064	2.19254036
9.329035	6.22009	21.284419	1.32929477
24.21525	4.017929	9.2875131	1.46909526
20.7712	5.771258	12.340742	2.18754604
45.86567	9.84439	15.646321	2.62858983
37.00173	15.80284	21.14447	4.10816586
22.59908	7.581061	13.256554	2.30028427
22.98947	13.99484	10.471441	2.5303092
43.52089	9.31245	17.841011	3.39851641
44.36669	13.78582	21.114876	2.84886776
31.32575	14.19338	19.831589	1.98980824
23.90925	9.66577	16.178589	2.71310351
34.77822	10.78769	13.460918	2.42469018
23.84532	10.44378	17.101126	1.99499063
19.93224	9.579246	12.335152	2.20191306
18.81283	3.201852	7.3811683	1.18624817
34.62287	4.595389	12.95873	1.40784099
19.08198	3.315281	11.524773	1.42071079
23.62727	5.681961	14.234385	2.37987159
28.45985	8.256219	16.260411	2.40057874
26.11508	14.43674	16.660888	2.04104246
30.32295	8.866115	15.727243	2.09458773
20.21524	9.607196	17.727379	3.18428107
30.15709	8.749928	19.751106	4.29755293
36.69186	5.923517	17.111519	2.18467271
36.38329	7.863554	16.380444	5.13183545
23.90319	4.795392	9.4874069	1.80201634
29.76588	10.1753	5.4509351	2.08496445
29.52524	15.68048	27.292002	3.05678562
28.98398	12.38723	14.036399	1.99721522
26.90606	2.845515	15.715318	2.07451868
27.77043	6.23096	15.662692	2.23881381
31.05093	3.644591	12.57985	2.73969433
38.38965	15.30679	17.211847	3.79003853
38.65363	21.6084	16.353239	2.72486621
46.84612	11.60908	15.222471	2.7000274

37.86174	12.50173	11.944425	2.23445403
14.65011	2.075598	4.2984423	0.91874985
29.90645	14.15463	8.8628776	3.44089931
37.67502	10.14116	17.056907	2.44342615
29.88864	7.323481	9.8879976	1.82459771
34.45653	13.94723	13.718697	2.55819049
11.12327	6.580626	17.971403	1.42027771
39.59204	5.989289	9.7803863	2.41886715
26.23743	8.938329	11.226224	2.76697564
29.59931	9.950426	12.415637	2.64153701
22.17124	10.93202	11.963558	2.40469158
43.60847	9.503233	16.609344	3.39707968
25.15458	11.20534	10.062728	1.74009293
36.58475	11.37036	15.598597	2.50712571
23.55774	13.05214	14.177275	1.69191839
20.44901	11.60253	13.208667	2.08496279
28.51306	9.20903	22.23988	3.68322441
33.57936	11.89211	13.195829	3.24438089
31.61345	8.573044	14.599961	2.61833259
21.85563	9.200654	11.864996	2.43965482
13.09116	1.28358	5.1235587	2.73981386
21.57744	6.582471	12.822339	1.53105881
31.86381	11.81394	13.815159	2.41460448
23.45326	8.895896	15.122423	1.93162931
20.05855	7.841745	11.232721	1.7205418
25.05343	8.834594	15.661349	2.70624264
26.92428	11.34304	15.288893	1.611853
32.44794	14.01899	13.387002	2.1800098
23.19415	5.951737	11.258312	2.34189678
31.90603	12.66569	16.241859	2.51774496
37.78759	9.273453	16.830002	3.23411325
23.43622	3.494851	8.0949028	1.27546763
30.84907	3.572113	10.853342	5.25336818
36.3012	5.53013	13.977436	6.34724204
41.32719	9.671732	19.787373	3.34032653
43.59976	14.53958	23.032539	9.96280781
52.77114	11.55143	29.915239	15.8857151
28.62528	5.456927	19.095339	1.63788021
45.78002	12.64883	25.292792	1.74934992
43.04419	10.96155	17.287392	2.54065511
31.3537	15.95226	15.311201	2.5754927
24.05961	22.14568	14.200261	2.66054319
32.88161	18.94784	11.228922	7.05598027
34.85926	20.76961	22.346187	13.5711896
25.37095	10.97851	12.501257	2.16798106
33.26159	12.46327	13.611858	7.87715225
38.90465	12.9222	25.12106	12.0968591
25.11503	9.409322	13.356582	3.01267709
22.5442	16.42023	11.035617	2.51791862
24.76338	9.777068	8.2331688	3.08414865
39.8677	7.550828	14.691798	2.61091226
34.86218	15.63745	12.566214	3.10266179

39.0147	8.161971	14.919609	3.01019003
24.57716	8.957977	16.440884	2.1060897
38.57495	14.95667	14.881226	1.74080758
23.91782	3.211576	10.008479	2.1472034
23.37686	15.34473	19.260626	2.14155187
28.41668	10.71267	14.725699	1.6268924
34.63207	12.05046	12.670974	2.07412369
25.80727	9.74705	15.745469	2.04355851
36.71306	6.956487	24.975684	2.3786325
46.46379	8.326209	25.774719	4.21820289
38.50375	12.81443	15.658605	4.28563659
40.14185	9.936963	21.470034	2.14565775
26.68681	7.469973	12.707467	2.4999052
40.21375	7.25082	24.03857	1.41649077
27.36381	5.518036	13.013137	1.18163116
37.20519	13.80556	12.166108	3.6000087
15.64141	8.127641	9.7994809	1.86328637
32.77521	13.29106	15.000252	2.25059043
22.00219	14.84391	16.690933	1.90106831
32.52936	11.35606	13.543086	2.14641016
26.58926	7.576413	18.309554	2.54051822
23.60269	7.686677	13.132639	1.93348664
24.8381	15.11115	21.430031	3.3434265
37.10906	9.320479	13.430911	2.60122981
24.69643	9.760404	14.04525	2.27318894
25.36954	6.698846	12.656941	2.21173844
29.50003	13.89069	12.812575	2.83536933
23.45078	5.913751	16.417263	1.80172501
22.40707	11.80837	14.64847	3.40272489
28.28202	9.538821	14.540308	3.14694458
26.48732	18.82532	16.595435	2.72981149
32.89165	5.941965	15.236109	2.49451369
12.47337	5.582846	11.838843	2.72759485
45.86979	8.535719	17.834818	1.71530883
29.85606	8.805096	11.740312	3.28658771
27.63454	10.08814	14.019393	2.10173571
16.29538	8.123824	13.304497	1.27086698
32.59777	8.810612	16.983663	2.18384834
41.84789	6.712598	12.386002	1.99380707
40.18466	8.563803	26.775354	3.48468719
25.31779	6.595614	13.480633	2.8776406
40.31998	7.083835	15.268589	2.10222122
31.08412	3.934741	11.422992	1.67427929
32.69153	9.612544	15.057722	3.22805675
33.55206	10.47419	16.76947	3.22975423
33.38746	10.87952	17.823822	3.08872439
34.68162	13.89054	23.481882	2.24076838
44.45443	10.86076	16.28804	2.31658948
29.76535	5.457572	10.963193	2.38215159
42.17432	7.331576	12.738722	1.93506611
15.77746	3.579933	9.200738	2.17705576
38.56327	10.17211	21.753621	2.5667573

31.48032	10.82957	18.719992	2.6746013
26.4123	8.780848	18.515469	3.40650343
31.40556	24.31171	15.050079	2.12953489
39.63101	10.74047	14.064128	2.0463534
37.54155	7.879396	12.864113	1.73667598
34.09421	8.43925	9.8125189	3.48998661
29.1522	10.79589	17.550899	2.56614436
30.50595	8.713841	8.7756605	1.86763049
35.54184	9.666029	15.430961	2.19478451
38.12687	9.526396	14.733106	2.76050201
32.29668	11.4884	10.612379	3.24674255
35.71148	6.713077	17.455891	2.23357189
10.61235	0.990922	4.5867475	1.61063575
31.50264	7.131583	16.298539	2.61831485
25.77998	5.384314	12.689782	2.05137978
21.71586	11.115	11.345611	1.40033114
32.9051	13.46311	17.861508	3.27040265
28.67143	6.252175	15.510977	2.47457288
32.13234	6.692955	9.3680993	1.90921243
34.36253	7.567197	16.38146	1.90688342
38.28254	9.810921	17.164173	3.02440361
24.50479	5.480823	13.59103	1.93285874
30.17416	9.297075	11.457055	3.42043198
34.42097	12.45938	18.757854	2.69432946
31.35932	8.7384	17.31749	2.14398224
35.17466	7.329684	14.533464	2.63606207
21.48875	10.22639	11.322489	2.38232751
39.74579	13.10042	17.406528	1.38648521
30.97272	5.854641	25.325891	3.20604514
40.37022	6.603444	15.343667	1.8503003
29.18126	10.00555	10.063154	2.9089562
25.78611	7.672447	11.699868	2.38398204
38.71846	7.295565	11.240468	1.79370659
29.19266	7.596776	14.384043	2.80959133
8.651923	4.153733	6.4820289	1.47062718
34.15733	8.159851	13.416502	2.64477549
33.28681	14.95001	21.685134	2.16889576
40.26288	9.016186	25.991327	2.67774387
27.35738	9.805888	13.131587	4.04374333
34.00053	17.89844	15.486486	1.97628633
23.30686	8.171939	16.04188	3.05588024
16.50676	2.760036	15.569004	1.67190619
25.76904	6.41535	20.424439	2.49590853
38.89827	8.448942	20.495536	4.79493525
39.19998	8.449476	13.644839	5.10748517
27.06858	5.516454	17.809451	2.82893602
27.22162	6.235395	12.001489	2.3228817
35.49165	12.53831	9.2235048	4.30210649
19.73995	15.10194	12.726928	1.97375734
29.39729	6.529928	15.477358	2.53591889
19.35624	10.71563	11.946715	1.71045144
35.31383	9.300537	14.546088	3.18355343

34.75485	10.59666	15.230639	4.16076688
24.5497	7.085039	12.024259	1.4064993
19.46131	7.102409	16.598479	3.12860805
23.92307	20.85322	15.97074	3.14825953
17.50502	3.153108	15.310669	2.30029926
27.02541	13.53473	13.937483	2.9833583
28.83493	10.38692	13.010847	2.0800588
24.31257	7.935588	12.989617	2.07671475
26.24639	10.05021	13.385305	3.14644904
19.27016	10.94941	9.7058936	2.87004749
18.83049	4.658489	12.968412	1.44470701
36.62618	9.609703	15.507117	3.07096849
14.9657	5.703853	9.9108908	2.09181837
32.28174	9.775586	9.0678621	3.18626854
35.27898	11.59871	15.494218	2.58396329
29.72027	15.40284	13.915855	2.50818332
33.21159	15.20469	11.109845	2.11977526
30.11216	6.192353	15.390127	2.05943014
31.32973	5.563032	9.9167031	2.14773907
24.45578	14.3748	14.574695	1.9770651
25.51517	5.383587	13.410504	2.51153027
29.42743	11.61029	13.290654	3.15081201
34.54626	10.00781	18.352294	2.62278741
40.83137	8.288378	18.395515	2.80175462
38.09548	13.62259	13.72535	2.84425748
40.43551	13.93738	11.251125	2.75672709
34.23033	14.3245	14.682323	2.95082568
21.64706	7.884857	15.764201	1.96908529
33.44408	12.83699	16.471922	2.91112255
34.45039	3.186045	7.8864819	5.0487842
22.89774	4.755012	15.090969	1.77448788
35.90401	10.03448	12.477714	2.52458422
33.42209	10.86641	9.427552	3.24221416
27.57647	8.461446	20.166916	3.27791062
36.17335	9.28764	16.751924	3.36184049
24.81148	2.39592	21.619517	2.65153186
29.22432	9.655697	10.49209	2.17233953
27.86515	6.919172	9.4978922	2.78051499
30.42677	8.929836	10.733983	3.29911066
34.33538	12.76233	15.821658	3.11697923
40.04423	13.02199	16.904811	3.42410443
16.19386	7.363926	9.6393191	1.81192304
25.04563	3.827976	20.856255	1.97629354
37.237	14.33151	17.311306	2.52671653
34.97568	14.3399	20.531539	3.26649814
35.3838	10.80878	13.383391	2.11384622
37.27491	8.938139	17.749133	4.18862033
19.57842	9.063725	15.169893	2.12451863
18.4738	4.130362	11.186001	1.55211078
30.87606	12.76726	13.858599	2.46615021
13.88564	5.324176	6.118049	2.03242807
37.78777	9.431587	13.560892	1.90217264

36.06481	10.57375	12.089508	2.16307943
19.82691	9.910243	14.210691	1.54826541
12.18158	4.428759	13.983734	1.18707654
17.80573	8.265596	13.272931	1.63397106
32.67575	7.415314	10.182567	1.42676346
16.32218	7.975503	8.854905	1.6065743
28.60737	12.35785	8.550764	1.98227257
27.24862	10.40383	7.0988908	2.15842864
26.38108	8.82	12.080541	2.65073553
23.9494	16.54725	13.478644	2.2985912
30.15682	8.337373	14.797458	2.80414247
16.32851	4.30571	5.5610186	1.96638847
30.47884	10.80357	15.051016	3.01264922
35.51157	12.92273	21.643267	2.46886227
24.58943	12.84723	22.311973	2.45428934
19.56582	8.420585	9.7511482	1.24459675
15.94503	4.011976	7.5651766	1.54422351
39.46666	15.25317	12.663614	3.23727964
36.39206	12.15403	13.859436	2.43338109
25.34818	11.53207	16.061382	2.06509689
26.73205	22.59878	18.36778	2.70698941
22.87154	15.71342	17.440245	2.93262151
33.39417	5.996961	14.811492	3.44503564
30.45833	12.68185	17.600068	2.93839108
28.88835	13.40957	12.509462	2.9903056
37.72878	9.405676	18.550248	3.3426222
32.5761	11.95006	10.182976	2.78822816
35.67308	8.438409	12.920748	2.74085199
25.33073	8.457925	13.860354	2.04721612
30.61946	15.21045	15.688129	4.40172426
16.20762	4.678641	10.869644	1.69484609
42.72487	8.810697	15.319136	3.53688447
25.10568	7.671842	16.85453	3.93944952
28.03423	10.76041	18.582149	2.71952677
29.42216	6.273755	14.762922	1.7340001
32.97365	11.03318	15.934304	2.89392509
24.01428	12.90788	14.699612	2.44025944
20.73797	6.663688	16.170476	2.33937599
17.8483	11.37964	14.282134	1.77734485
42.772	8.942254	11.928275	2.0867385
24.99058	9.347659	16.156868	2.43217195
30.82017	9.637199	13.756293	2.15182363
24.86081	6.00453	10.720603	1.90749081
27.97483	15.03838	16.344703	1.96569624
23.12718	5.171286	15.226906	1.85310934
31.78741	8.895077	12.221098	2.6974555
25.45303	7.376815	21.240293	3.32955232
21.17787	5.347946	14.336787	2.38432351
20.17843	8.143592	14.832199	2.6889571
23.92227	10.45112	18.287797	2.36144372
28.31819	20.5604	18.099096	1.98280256
29.87771	11.58847	15.697072	2.22388475

22.82925	5.898448	9.440147	2.13860947
20.62992	7.963485	26.225258	2.4885385
24.75145	10.4002	14.30207	2.6008286
22.75645	9.445615	16.857993	2.34109996
12.0484	3.847847	18.792901	1.8568378
30.26481	8.689111	10.031518	2.30386199
23.26659	12.18163	10.755309	2.24077462
32.75982	13.36054	17.782759	3.00544197
23.33873	8.829981	15.388739	2.1855172
34.70237	11.15198	10.864979	1.69929644
34.50411	12.13885	16.481754	2.420832
27.8184	10.78564	20.065055	3.12237839
29.64781	10.99607	12.005736	2.80731227
29.74457	7.538956	13.378219	1.96652074
35.19707	16.42305	12.991559	1.92831838
34.31755	12.45011	17.551925	2.24553191
33.75797	10.06383	13.182108	2.05988762
33.85415	10.3501	13.601372	1.83912232
34.11156	10.36975	16.445075	2.4727766
19.22943	9.799992	15.651005	1.43390423
30.23439	9.70405	17.998482	2.7765613
29.66838	9.921166	13.521398	2.36440171
30.3907	12.18714	14.187016	3.7089252
20.15118	12.06655	10.787466	1.72806735
16.87279	11.66117	11.260389	2.14042743
25.41647	8.844893	10.194697	3.14471347
22.91517	13.231	12.631452	1.45381881
17.2345	11.94425	13.436839	2.14897914
29.63454	11.17947	16.411114	2.11429216
18.44331	12.36136	6.8002922	1.71716307
21.46826	10.42657	7.78375	2.38138508
20.709	4.846391	14.493099	3.32633796
22.68036	16.30372	10.437899	1.89784457
29.63721	9.053453	14.317788	2.21823495
38.42641	15.56149	13.21978	2.48106216
25.87664	13.15896	18.491387	1.70152654
18.66544	8.111111	14.360976	2.01614848
24.73024	11.10042	11.898954	2.69527842
36.32084	10.07722	15.188561	3.58679579
25.97547	14.59208	21.016192	2.1359677
25.48236	5.776497	14.03626	1.74683942
24.9966	10.2307	16.548235	3.09663907
21.57357	10.91381	13.45552	1.93558612
30.7802	23.91173	18.03158	2.93134494
23.59597	12.79637	12.397194	2.61163316
33.98864	13.89578	12.070187	3.3833109
21.33518	16.2253	7.9482904	2.79307818
27.36793	10.44852	7.885779	2.39773117
24.58514	16.47099	12.395701	2.94477946
30.10032	11.50249	12.743105	2.63064443
32.47391	9.928034	14.097818	2.40856235
34.44167	16.72562	13.044719	2.21361651

19.08181	7.151793	13.3405	3.15737456
23.57638	5.845655	30.502301	3.18095939
29.62155	11.98141	14.937087	2.89151343
47.75401	11.11651	19.392244	1.79810658
41.44557	10.31452	21.350642	3.69934719
24.52359	12.43954	20.661019	2.32713662
33.82277	13.77101	8.3609453	2.57511231
34.45415	7.671467	11.497464	2.01864379
24.80696	12.07552	12.260946	1.96565325
34.48743	8.355168	12.592976	2.54291461
24.03552	9.553813	9.5174899	2.65622811
33.86225	17.11087	7.4257348	3.51121633
34.51726	10.86321	22.535869	2.02211562
38.50997	7.938439	16.917229	3.05886665
26.31743	10.2937	16.564653	3.08382574
28.55161	12.14853	16.300637	2.95199924
35.40828	12.97663	11.612738	3.11952485
26.47405	7.933529	15.256737	2.38646827
30.47659	7.291793	13.699149	1.95401713
28.65098	11.82266	12.945549	2.00770325
26.98794	8.89653	11.264433	2.03576685
31.46706	8.40342	12.685289	3.43400701
42.38423	6.956994	15.326328	1.6437025
30.69081	16.01711	17.863525	2.61925071
23.35661	13.96451	7.9799072	2.45551152
35.60307	6.654068	20.578072	1.82309302
30.60586	12.73385	13.533383	1.59346343
33.09347	7.479485	14.50259	2.54285367
27.1578	9.793944	9.3690257	2.49933082
29.04504	13.44626	14.090543	2.50088863
19.91028	14.22105	14.23605	1.75962374
46.17355	13.04158	13.384577	2.67209246
28.2629	6.539497	11.500737	2.0366406
54.01858	19.03617	13.359538	4.04842364
24.74805	4.534486	11.356829	2.66549633
41.93817	12.48614	20.101278	2.32775325
15.97292	3.912519	12.209651	1.68167935
30.34101	7.49214	11.099849	1.54043774
28.39116	7.483783	13.059248	2.36139393
30.44333	9.576479	16.220716	2.70396425
26.87342	16.08331	10.728177	1.77987004
25.03299	14.06515	8.8597657	2.24109437
50.13966	15.44679	16.937902	4.53072001
19.46048	9.514948	12.467796	2.45172229
23.45889	8.896809	13.149438	2.35278838
36.62991	11.97669	20.999262	3.6537772
21.68517	4.670048	11.317794	2.50202216
32.69529	12.14591	15.545237	3.73737986
30.65706	11.68656	13.534778	2.91279555
28.53403	5.751239	16.311143	2.5881399
35.40843	8.789501	16.088801	2.87830212
32.59223	12.34413	13.000153	3.46337707

23.42044	7.05851	15.498926	1.83647072
34.01034	12.88791	13.027819	2.98918226
26.74085	9.161207	13.904831	2.51975733
19.48345	8.290438	15.263456	1.86484992
32.66049	2.981055	18.291511	1.91122424
37.46136	14.86358	20.988182	3.81328144
38.61902	6.264507	15.280663	1.96856958
29.5681	9.513705	15.204699	2.23209772
28.31436	9.719863	11.343736	1.37446076
30.63831	12.94561	16.09096	2.965776
29.96615	14.02005	9.4249958	2.10240805
18.40312	3.900933	11.641178	1.9760394
39.69646	7.146474	13.804586	2.19336254
12.3664	3.427521	9.8728663	2.92705333
33.61764	4.354477	9.6020603	1.74650321
31.38159	9.479238	18.453733	2.98315817
25.43042	7.590692	10.825374	2.21040807
36.78248	2.09034	11.113187	1.93295605
17.24858	6.494641	12.124988	1.70968845
35.77602	8.876258	12.108089	3.18813679
23.02378	8.384354	13.584508	2.07291684
